# Consistent probabilistic outputs for protein function prediction

**DOI:** 10.1186/gb-2008-9-s1-s6

**Published:** 2008-06-27

**Authors:** Guillaume Obozinski, Gert Lanckriet, Charles Grant, Michael I Jordan, William Stafford Noble

**Affiliations:** 1 Department of Statistics University of California, Berkeley, Berkeley, CA 94720, USA; 2 Department of Electrical and Computer Engineering, University of California, San Diego, San Diego, CA 92093, USA; 3 Department of Genome Sciences, University of Washington, Seattle, WA 98195, USA; 4 Department of Statistics, Department of Electrical Engineering and Computer Science, University of California, Berkeley, Berkeley, CA 94720, USA; 5 Department of Genome Sciences, Department of Computer Science and Engineering, University of Washington, Seattle, WA 98195, USA

## Abstract

In predicting hierarchical protein function annotations, such as terms in the Gene Ontology (GO), the simplest approach makes predictions for each term independently. However, this approach has the unfortunate consequence that the predictor may assign to a single protein a set of terms that are inconsistent with one another; for example, the predictor may assign a specific GO term to a given protein ('purine nucleotide binding') but not assign the parent term ('nucleotide binding'). Such predictions are difficult to interpret. In this work, we focus on methods for calibrating and combining independent predictions to obtain a set of probabilistic predictions that are consistent with the topology of the ontology. We call this procedure 'reconciliation'. We begin with a baseline method for predicting GO terms from a collection of data types using an ensemble of discriminative classifiers. We apply the method to a previously described benchmark data set, and we demonstrate that the resulting predictions are frequently inconsistent with the topology of the GO. We then consider 11 distinct reconciliation methods: three heuristic methods; four variants of a Bayesian network; an extension of logistic regression to the structured case; and three novel projection methods - isotonic regression and two variants of a Kullback-Leibler projection method. We evaluate each method in three different modes - per term, per protein and joint - corresponding to three types of prediction tasks. Although the principal goal of reconciliation is interpretability, it is important to assess whether interpretability comes at a cost in terms of precision and recall. Indeed, we find that many apparently reasonable reconciliation methods yield reconciled probabilities with significantly lower precision than the original, unreconciled estimates. On the other hand, we find that isotonic regression usually performs better than the underlying, unreconciled method, and almost never performs worse; isotonic regression appears to be able to use the constraints from the GO network to its advantage. An exception to this rule is the high precision regime for joint evaluation, where Kullback-Leibler projection yields the best performance.

## Introduction

The computational prediction of protein function can provide an essential tool for the biologist, because many biological questions are directly answered when we understand the role of a protein in a biological process, how it interacts with other proteins and DNA, and where in the cell it operates. Given the limitations of current predictive methods, however, the purpose of such technology cannot be to replace experimentation, but rather to assist the biologist either by directly generating hypotheses to be verified experimentally or by suggesting a restricted set of candidate functions that can guide the exploration of promising hypotheses.

Our general strategy, which is similar to that of several other groups that participated in the MouseFunc assessment [1], involves first predicting protein function (that is, Gene Ontology [GO] terms) on a per-term and per-data set basis and then combining the predictions in a post-processing stage. For the individual predictions, we employed the support vector machine (SVM) [2], using kernel methods to convert the different data sources (sequence motifs, experimental phenotypes, protein-protein interactions, differential gene expression levels, and orthology relationships) into a numerical format appropriate for the SVM. Our focus in the current paper, however, is not the SVM methodology *per se*, but rather the methodology for combining per-term predictions. Indeed, very little in our presentation hinges on the choice of the SVM and kernel methods for the individual prediction. Any method that can return a probabilistic estimate could be substituted in place of the SVM.

Let us consider some of the general desiderata for any method that yields predictions of protein function. First, we aim for any such method to be consistent with the GO. Specifically, a set of predictions is consistent with the GO if the predictions increase in confidence (for example, in posterior probability) as we ascend from more specific to more general terms in the GO. For example, a protein that is predicted to be in the nucleolus should also be predicted to be in the nucleus, and as a result the confidence in the latter prediction should always be higher. Second, we aim for such methods to be well calibrated, in the sense that the confidence assigned to a prediction provides a good estimate of the prediction being correct; in other words, we wish to construct confidence values that can be interpreted as probabilities that a protein has a certain function given the information provided by the data. Third, and most importantly, we desire a method whose predictions are accurate. To measure accuracy, we use two complementary metrics: precision (or positive predictive value), which measures the fraction of predictions made that are correct, and recall (or sensitivity), which measures the fraction of the correct answers that are predicted. In this work, we fix four specified recall values (R = 1%, 10%, 50%, 80%) and measure the corresponding precisions.

In addition to these general aims, the quality of a prediction method's output depends upon the particular prediction task at hand. Therefore, in this work, we distinguish three prediction tasks and define three corresponding modes of evaluation: per protein, per term and joint annotation. In the per-protein mode, for example, a developmental biologist has determined a few genes that are activated by a particular regulator, and the biologist wants to understand which biological process is regulated and how it relates to the phenotype observed. Given a certain protein, a prediction for its function is needed. In the per-term mode, for example, a drug designer has determined which biological process is involved in a pathological condition and is now looking for a drug target in that pathway. Given a function, a prediction for which proteins have that function is needed. In the joint annotation mode, for example, a bioinformatician is annotating a new genome and wants to guarantee a high level of accuracy in the predictions made. To achieve this goal, some proteins that are harder to classify or some functions that are harder to predict should be subject to a smaller number of predictions. In particular, if the confidence for all predictions can be estimated on the same scale, then only the most confident predictions should be considered, assigning proteins to functions. Given protein-function pairs, correct associations have to be predicted.

To match these three different types of tasks, we propose three performance evaluation modes: per protein, the average, across proteins, of the precision at a fixed term recall; per term, the average, across terms, of the precision at a fixed protein recall; and joint annotation, the precision at fixed recall for assignments of proteins to functions.

In addition to these three evaluation modes, our analyses follow the distinctions used by Peña-Castillo and coworkers [1]. In particular, we consider separately the three ontologies that comprise GO: biological process, cellular component and molecular function. We also subdivide predictions into four groups on the basis of the number of proteins assigned to a GO term (3 to 10, 11 to 30, 31 to 100, and 101 to 300 proteins).

Overall, we consider 12 different protein function prediction methods. These include the baseline, unreconciled predictions, and three heuristic methods that return consistent probabilistic predictions (that is, predictions that increase numerically as we ascend the GO hierarchy). We also consider four variants on the Bayesian approach first applied to GO term prediction by Barutcuoglu and colleagues [3]. We consider one discriminative method that extends logistic regression to the case of interrelated outputs. Finally, inspired by the work of Wu and coworkers [4] for multi-class classification, we also propose three methods, based on Kullback-Leibler projections, that transform probabilistic values obtained separately for each GO term into probabilistic values that yield predictions consistent with the GO network topology.

Given the large number of prediction methods considered and the resulting multiple testing problem, we employ a two-pass strategy to identify statistically significant trends. Before testing the methods on the test set, we perform a preliminary evaluation on a held-out portion of the training set. When we draw conclusions about the various methods in our experiments, we retain only conclusions confirmed to generalize to the test set, which should be expected to be a more difficult set than the held-out set, because the latter has exactly the same distribution as the training set.

Following this strategy, we reach the following primary conclusions. Isotonic regression generally performs well across evaluation modes, term sizes, ontologies and recall levels. In particular, isotonic regression usually performs better than the underlying, unreconciled logistic regression method. This implies that reconciliation need not yield a decrease in performance; indeed, the structure of the GO network can yield valuable information that improves classification. Isotonic regression also typically performs better than many other reconciliation methods, which frequently yield reconciled probabilities with significantly lower precision than the original, unreconciled estimates. If the high precision regime of the joint annotation mode is of interest, then the Kullback-Leibler projection should be preferred, because it performs significantly better than isotonic regression, and thus better than logistic regression as well. This evaluation regime is of particular interest because it yields predictions with the highest precision of all evaluation modes. Overall, the Kullback-Leibler projection is a competitive reconciliation method. For 'small' GO terms - to which few proteins have been assigned - this method also yields better performance in comparison with all other methods.

## Results

### Independent predictions are frequently inconsistent

**Figure 1:**
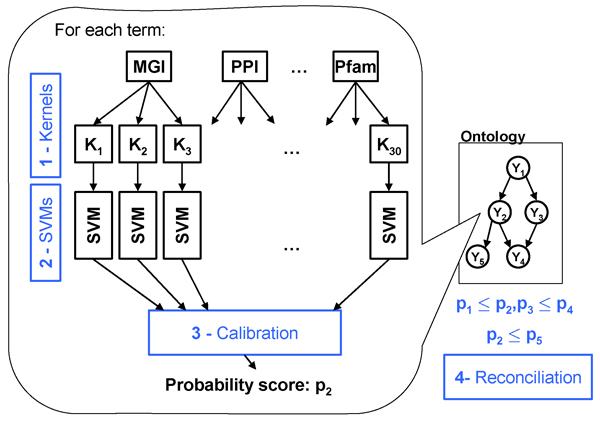
Overall approach. (1) A kernel matrix is computed for each available data type, and (2) these kernels are used to train one support vector machine (SVM) for each term and each data type. (3) The SVM predictions are combined and calibrated via a collection of logistic regressions. (4) Finally, the calibrated predictions are reconciled with respect to the Gene Ontology (GO) topology.

Our approach consists of four steps, schematized in Figure 1. Initially, we consider only the first three steps, omitting the reconciliation in step four. In the first step, each data set is transformed into several 'kernel matrices'. The SVM algorithm uses a generalized notion of similarity, known as a kernel function, to implicitly map data objects (vectors, strings, nodes in a graph, and so on) into a high-dimensional vector space [5]. A kernel matrix is a sufficient representation of the data for the SVM and is computed using the kernel function. The ten data sets in the benchmark are summarized briefiy in Table 1, and the corresponding collection of 31 kernel functions is listed in Table 2. In most cases, we compute three kernels: a linear kernel, a Gaussian kernel, and a kernel specifically tailored to the given type of data. In addition, we build four kernels that are linear combinations of the previously described ones. Details of the various kernel transformations are given in the Kernels section of Materials and methods.

**Table 1:** Summary of data types

Data type	Description	BP	CC	MF
**Phenotype**				
MGI	Mammalian phenotype ontology terms (33)	1,994	2,157	1,898
OMIM	Diseases (2,488) associated with human homologs	998	1,166	978
				
**Phylogenetic profile**				
Inparanoid	Orthologs across 21 species	6,131	7,092	6,556
Biomart	Orthologs across 18 species	6,269	7,242	6,695
				
**Protein domain**				
Interpro	Functional sites and domains	7,131	8,027	7,603
PfamA	Protein domains	6,790	7,648	7,239
				
**Protein-protein interaction**				
PPI	Transferred via orthology from human (OPHID)	3,273	3,690	3,509
				
**Gene expression data**				
Su *et al*. [9]	Oligonucleotide arrays (55 tissues)	6,555	7,587	7,029
Zhang *et al*. [7]	Affymetrix arrays (61 tissues)	5,097	5,716	5,447
SAGE	Tag counts from SAGE library (99% cutoff)	6,323	7,231	6,753
				
**Total**		7,968	9,005	8,427

The table lists the ten data types from [1], along with the number of proteins that are annotated with at least one term of each ontology and for which that data type is available. BP, biological process; CC, cellular component; MF, molecular function.

**Table 2:** Kernel transformations

Name	Linear	Normalized linear	Linear^2^	Normalized linear^2^	RBF	Diffusion	Parameters
MGI	✓	✓					
OMIM	✓	✓			✓		*σ *= 1
Inpar	✓	✓			✓		*σ *= 1
Biomart	✓	✓			✓		*σ *= 1
Inter	✓	✓			✓		*σ *= 1
PfamA	✓	✓			✓		*σ *= 1
PPI	✓	✓				✓✓✓	*τ *∈ {0.1, 1, 10}
Su		✓					
Zhang	✓	✓	✓	✓	✓		*σ *= 1
SAGE	✓	✓			✓		*σ *= 1

Each row in the table corresponds to one of the ten data types listed in Table 1. Check marks indicate which kernels were computed for each data set. The kernels are described in the Kernels section in Materials and methods. 'Linear^2^' and 'Normalized linear^2^' refer to the squared version of the original kernel matrix, as described in Materials and methods. The three check marks for the PPI data indicate that three diffusion kernels were computed using *τ *∈ {0.1, 1, 10}. RBF, radial basis function.

In step two, SVMs are trained for each GO term and kernel. However, in order to use SVM outputs for a further learning step, we need to simulate with training data the distribution of SVM outputs on new data. This prevents us from using the whole training set to learn just one SVM per term and per kernel, because the distribution of scores that the SVM assigns to the training and testing points differ. We therefore proceed as in cross-validation, repeatedly holding out data on which the SVM is tested and using the remaining training points to train the SVM. Details of this procedure are given in the SVM training section in Materials and methods. We consider all terms with 3 to 300 annotated proteins, leading to a total of approximately 780,000 trained SVMs.

We use a logistic regression in the third step to produce individual probabilistic outputs from the set of SVM outputs corresponding to one GO term. To handle missing data, we cluster the held-out (or test) proteins into groups of proteins with similar patterns of missing data, and we train a logistic regression for each of these groups, following the scheme described in the Missing data section in Materials and methods.

The assessment described in [1] was conducted in two stages: a training phase where only labeled training data were available and a subsequent test phase in which unlabeled test data were distributed to the participants. Although we performed many of our analyses after the official training phase had ended, we restricted our initial analyses to a held-out portion of the training set, composed of a fixed set of 2,000 randomly selected proteins.

**Figure 2:**
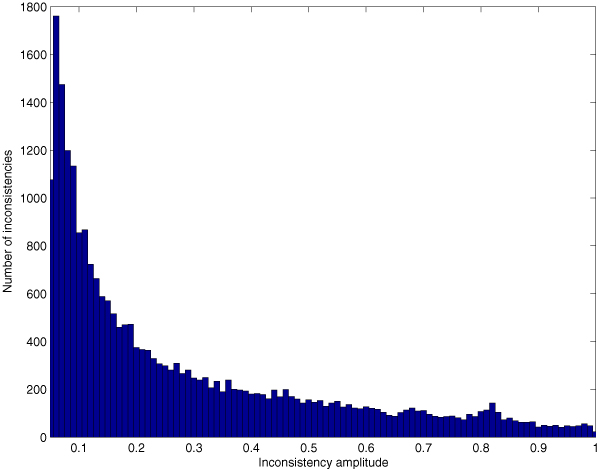
Distribution of large positive differences between child and parent GO term estimated probabilities. The figure shows a histogram of the top 5% of the distribution of differences between the probability assigned to the child term and the probability assigned to the parent term by the logistic regression, corresponding to differences that are larger than 0.05. GO, Gene Ontology.

**Figure 3:**
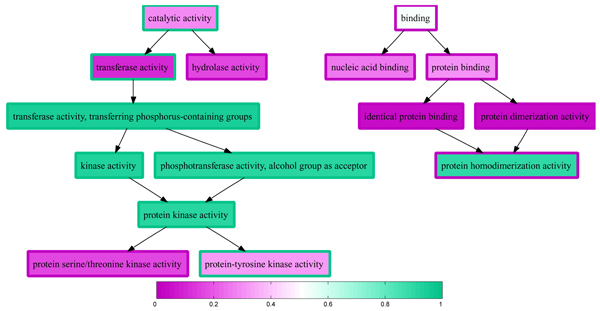
Inconsistent predictions for the s domain of casein kinase 1. The graph shows a portion of the molecular function Gene Ontology (GO), corresponding to positive labels and labels predicted with probability larger than 0.1. Each term's outline is colored according to the label with respect to the target term 'protein-tyrosine kinase activity'. A green outline corresponds to a positive label, and a purple outline corresponds to a negative label. For a given protein from the held-out data set, the interior of each node is colored following a similar color scheme (green = 1 and purple = 0), according to the probability produced by several data specific support vector machines (SVMs) combined into a logistic regression.

We applied our three-step procedure to the held-out data set, generating predictions across all three ontologies (2,931 terms) for each of the 2,000 proteins. Among the resulting set of 8.83 × 10^6 ^parent-child term relationships, 10.96% are inconsistent, and a significant number (4,645) of these inconsistencies - more than two on average per protein - are large, with a difference in parent and child probabilities greater than 0.5. Figure 2 plots the distribution of large differences in probability between child and parent terms. An example of this type of inconsistency is shown in Figure 3. For this particular protein, we observe three false negative annotations in the left half of the plot: 'catalytic activity,' 'transferase activity', and 'protein-tyrosine kinase activity.' However, these false negatives are offset by a block of strongly confident, correct predictions for four intermediate terms, colored in dark green. Ideally, a good reconciliation scheme would propagate the high-confidence predictions from these four terms to overturn the two parental false negative predictions. Conversely, on the right side of the plot, we observe a single false positive prediction for 'protein homodimerization activity,' which is a child of two very confident true negative predictions. Again, a good reconciliation scheme should fix this false negative annotation, using the two high-confidence parents to modify the prediction on the child term.

Previous work has shown that reconciling independent GO term predictions can yield improved accuracy [3]. However, note that, in addition to being inaccurate, the predictions shown in Figure 3 are difficult to interpret because they are inconsistent with one another. A method that claims, for example, that a protein has homodimerization activity but does not have dimerization activity is clearly incorrect, and a biologist attempting to interpret these results would likely not trust either prediction. Thus, even if reconciliation fails to improve the accuracy of our independent predictors, reconciled predictions are more desirable than unreconciled predictions.

### Reconciliation methods

**Figure 4:**
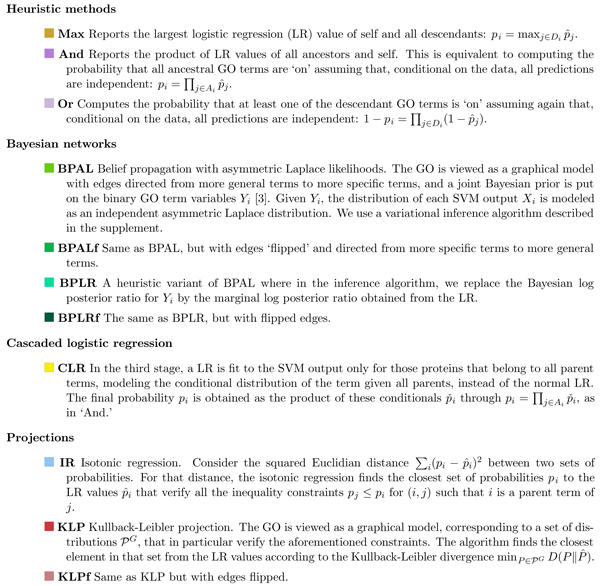
Summary of reconciliation methods. Given a set of probabilistic values obtained from logistic regression (${\hat{p}}_{i}$, *i *∈ *I*), and *A*_*i *_and *D*_*i *_denoting the set of ancestors and descendants, respectively, of Gene Ontology (GO) term *i*, we compute reconciled probabilistic predictions *p*_*i *_using the 11 strategies described in the text. Detailed descriptions of each method are given Additional data file 1. Colored boxes indicate the color that is used to represent this method on all subsequent plots.

Motivated by the inconsistencies produced by our independent GO term predictors, we proceed to the final step of the pipeline shown in Figure 1. In step four, the outputs of step three are processed by a 'reconciliation method'. The goal of this stage is to combine predictions for each term to produce predictions that are consistent with the ontology, meaning that all probabilities assigned to the ancestors of a GO term are larger than the probability assigned to that term. This fourth step is the core of our experiment, in which we consider 11 different methods, summarized briefiy in Figure 4 and described in detail in Additional data file 1. We can distinguish four types of methods: heuristic methods; Bayesian networks; cascaded logistic regression - a discriminative method that extends logistic regression to the structured output case; and projection methods.

We selected the 11 reconciliation methods to provide a variety of complementary approaches. In general, the problem of reconciliation arises for any structured prediction problem, in which a set of interdependent labels have to be predicted. Depending upon the structure being predicted, the interdependence relations among terms may be more or less complicated. In the case of GO terms, although the GO is a complex directed acyclic graph, the term relationships consist of simple deterministic implications, making the problem very intuitive. Several naïve heuristics are therefore natural. Beyond these heuristics, methods using Bayesian networks have been proposed by Barutcuoglu and coworkers [3]. However, discriminative methods usually perform better in classification problems; we therefore considered the cascaded logistic regression, which is the simplest of our structured discriminative models. Cascaded logistic regression itself has shortcomings, which motivated us to propose new methods based on projections. These projection methods are close in spirit to some of the naive heuristics, compatible with a discriminative framework, and related to a variational formulation of belief propagation. The remaining methods considered result from reversing the direction of the relationship between parent and children GO terms or were hybrid methods that we included in the evaluation in order to provide insights into the properties of the other methods.

All methods except the Bayesian networks and the cascaded logistic regression take as input the logistic regression estimates of the posterior probability for GO term *i*:

$${\hat{p}}_{i}=P(Y_{i}|X_{i}=x_{i})$$

where *Y*_*i *_is a binary variable indicating whether the protein has the function corresponding to GO term *i*, and *x*_*i *_contains the outputs of the 35 SVMs trained for term *i*; the Bayesian networks explicitly model the likelihood *P*(*X*_*i *_= *x*_*i*_|*Y*_*i *_= *y*_*i*_). The cascaded logistic regression uses individual regressions that estimate $P(Y_{i}|Y_{\pi_{i}},X_{i}=x_{i})$ or $P(Y_{i}|Y_{c_{i}},X_{i}=x_{i})$, where *π*_*i *_and *c*_*i *_are the set of parents or children of term *i*.

In the experiments described below, we performed analyses in the three modes described in the Introduction: per term, per protein, and jointly. For each of these evaluation modes, we considered the three different ontologies - biological process, molecular function and cellular component - and four different ranges of term sizes: 3 to 10 proteins, 11 to 30 proteins, 31 to 100 proteins, and 101 to 300 proteins. Furthermore, we compare methods at four specified recall levels: 1%, 10%, 50%, and 80%. In the following, we first describe systematically the three evaluation modes, and then proceed to more general conclusions.

### Per-term evaluation

**Figure 5:**
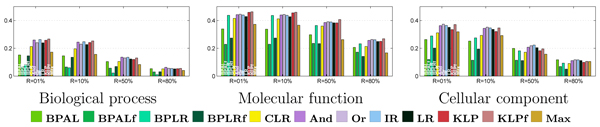
Per-term evaluation, irrespective of term size. Each panel plots the average precision across all Gene Ontology (GO) terms (y-axis), using four fixed recall levels (x-axis). The bars are colored according to the 12 methods, as described in Figure 4.

The primary results from the per-term evaluation are shown in Figure 5. The bar charts in this figure show average precision (y-axis) as a function of four fixed recall values (x-axis) for all 11 reconciliation methods, plus the unreconciled logistic regression. Some general trends are evident. In all three ontologies and at all four recall levels, at least one reconciliation method performs better than the baseline logistic regression. Top performers include the three projection methods (isotonic regression and the two Kullback-Leibler projections), as well as two of the heuristic methods ('And' and 'Or'). The belief propagation methods work consistently poorly, and belief propagation with logistic regression (BPLR) as well as the 'flipped' versions of both the belief propagation method with asymmetric Laplace (BPAL) and BPLR perform worst among all the methods.

**Figure 6:**
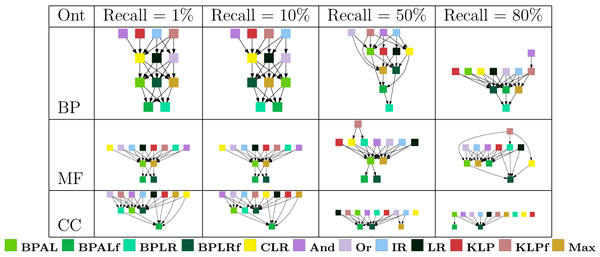
Statistical significance testing of per-term evaluation, irrespective of term size. Each panel shows a directed graph in which nodes are methods and a directed edge from node A to node B indicates that method A performs significantly better than method B according to the Z-test described in the Statistical testing section of Materials and methods. Because Z-tests are transitive - unlike other tests such as the Wilcoxon signed-rank test - we represent the graphs as transitive reductions, that is, removing edges that are already implied transitively by other edges. BP, biological process; CC, cellular component; MF, molecular function; Ont, ontology.

Making qualitative observations about the results in Figure 5 immediately begs the question, which of the apparent differences in the figure are statistically significant? To address this question, we use a Z-test procedure, described in the Statistical testing section in Materials and methods, which measures the average amount of improvement from one method to the next. The directed graphs in Figure 6 summarize these statistical tests. In each graph, an edge from node A to node B indicates that method A performs significantly better than method B according to the Z-test. These graphs confirm that the three projection methods perform well across ontologies and term sizes. For example, isotonic regression never performs significantly worse than any other method, and the two Kullback-Leibler projections (KLP and KLP with edges flipped [KLPf]) are each bested by a single method in one case (KLP by 'And' for the biological process ontology at 50% recall, and KLPf by 'And' in the same ontology at 80% recall). 'And' also performs very well overall; like isotonic regression, it never performs significantly worse than any other method.

**Figure 7:**
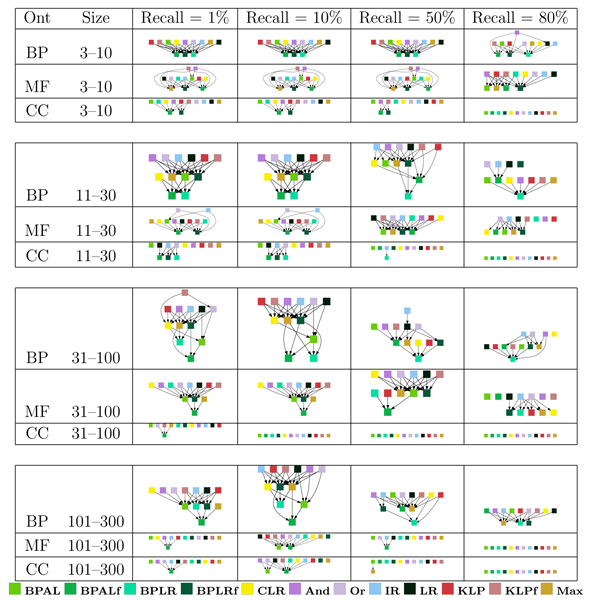
Statistical significance testing of per-term evaluation. Each panel shows a directed graph in which nodes are methods and a directed edge from node A to node B indicates that method A performs significantly better than method B according to the Z-test described in the Statistical testing section of Materials and methods. Because Z-tests are transitive - unlike other tests such as the Wilcoxon signed-rank test - we represent the graphs as transitive reductions, that is, removing edges that are already implied transitively by other edges. BP, biological process; CC, cellular component; MF, molecular function; Ont, ontology.

Thus far, we have ignored one dimension of our analysis: the subdivision of terms according to their specificity. Intuitively, some methods may be good at predicting very specific terms, for which few training examples are available, whereas other methods may excel at making predictions for very broad terms. When we subdivide the GO terms into four groups, based on the number of proteins in the training set that are annotated with that term, we obtain 48 directed graphs like the ones shown in Figure 6 (three ontologies, four recall levels, and four term sizes). These plots are shown in Figure 7.

**Figure 8:**
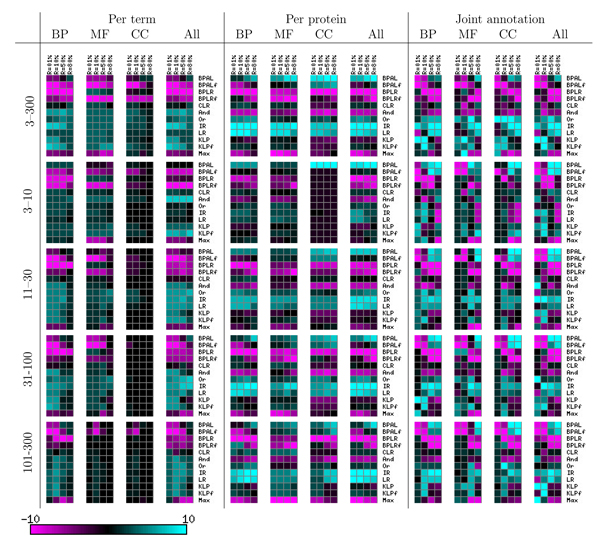
Summary of the entire experiment on the held-out data set by win-loss score. Each heatmap entry represents the win-loss score for a given reconciliation method (row) and recall level (column). BP, biological process; CC, cellular component; MF, molecular function.

The 48 separate directed graphs in Figures 6 and 7 are difficult to summarize concisely. We therefore performed an additional processing step, in which we count how many times each method wins and loses with respect to each of the other methods. Specifically, for a given directed graph from the Z-testing procedure, and for a given method A, we count (in the original, transitively closed graph) the number of outgoing edges (wins) and the number of incoming edges (losses), and we subtract losses from wins. The resulting win-loss score ranges from 11 (all wins and no losses) to -11 (*vice versa*), with a score of zero indicating that the method is approximately in the middle of the pack. This win-loss counting procedure has the advantage that it handles ties gracefully; when all methods tie, each method receives a win-loss score of zero. The win-loss score allows us to summarize the results for all three ontologies, all four term sizes, and all four recall levels in the left-most three columns of Figure 8. In addition to scores for the three separate ontologies, we ran the Z-testing procedure across all terms for all three ontologies and computed corresponding win-loss scores. These scores are shown in the column labeled 'All'.

Qualitatively, the most obvious trend in these heatmaps is the division of methods into winners (cyan) and losers (magenta). For five methods - the four belief propagation methods and the 'Max' heuristic - nearly all of the corresponding win-loss scores are either zero or negative. In the few cases where one of these methods achieves a positive win-loss score (for example, BPLR for the molecular function category and small GO terms), it does so by outperforming the other loser methods and tying with the winners. The second most obvious trend is that the biological process ontology provides more discrimination among methods than the other two ontologies. Indeed, for the cellular component ontology, so many methods tie with one another that most of the win-loss scores are zero. Among the six winning reconciliation methods, no clear trend emerges.

**Figure 9:**
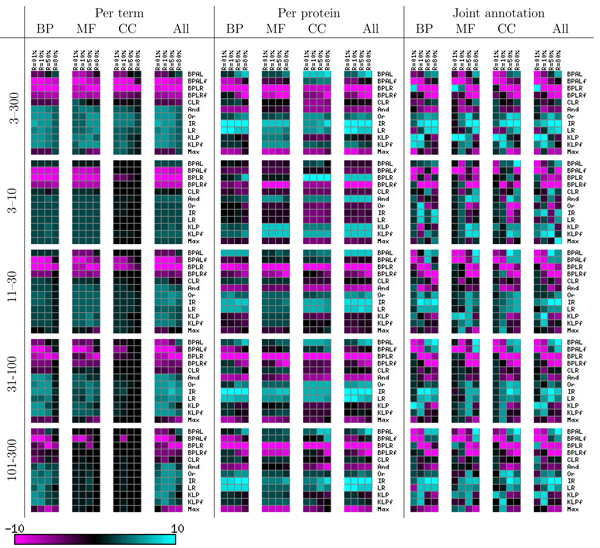
Summary of the entire experiment on the test data set by win-loss score. Each heatmap entry represents the win-loss score for a given reconciliation method (row) and recall level (column). BP, biological process; CC, cellular component; MF, molecular function.

Thus far, we have described only results on the held-out data set. We can gain additional confidence in our conclusions if they are upheld by the results from the test set. Figure 9 shows the win-loss scores for the test set. Again, we can immediately divide the methods into the same categories of winners and losers. However, it is also clear that the test set yields far fewer significant differences among methods. Once again, among the five winning reconciliation methods, no clear best method emerges.

### Per-protein evaluation

**Figure 10:**
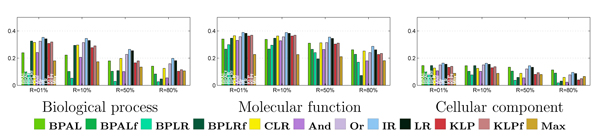
Per protein evaluation, irrespective of term size. Each panel plots the average precision across all protein in the held-out set (y-axis), using four fixed recall levels (x-axis). The bars in the top row and the nodes in the second row of panels are colored according to the 12 methods, as described in Figure 4.

The per-protein evaluation proceeds in a similar fashion to the per-term evaluation. Figure 10 shows bar plots similar to those in Figure 5, but with precision averaged across proteins rather than across terms. Statistical tests performed on these results yield directed graphs (Additional data file 2), which are then summarized using win-loss scores. These statistics are summarized in the middle four columns of Figure 8.

For the per-protein evaluation, the story is quite different from in the per-term evaluation. The two methods that clearly dominate in terms of precision are the unreconciled logistic regression and the isotonic regression. Other reasonable contenders are the BPAL and the heuristic 'Or' methods. However, an inspection of the individual tests (Additional data file 2) reveals that most of the time in the biological process and in the molecular function ontologies, the isotonic regression performs significantly better than BPAL. Isotonic regression also performs systematically significantly better than 'Or', which is also significantly outperformed most of the time by the logistic regression. On the other hand, the 'And' heuristic, which performed quite well in the per-term evaluation, is now a losing method, and even the Kullback-Leibler projections are borderline. The hybrid BPLR method and its flipped variant (BPLRf) are still losing methods. The only consistent winner among the reconciliation methods is the isotonic regression. Except for small term sizes, where many methods tie, isotonic regression nearly always performs as well or better than 'Or' and BPLR. These observations are confirmed in the test set (Figure 9).

### Joint evaluation

**Figure 11:**
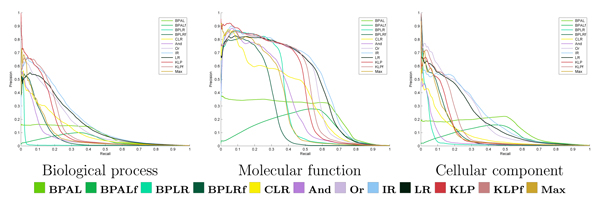
Joint evaluation, irrespective of term size. Each panel plots expected precision as a function of recall produced by ranking all (protein, term) pairs according to their predicted probability. The curves have been bootstrapped as described in the Bootstrap procedure for joint evaluation section in Materials and methods.

Finally, we performed a joint evaluation across terms and proteins simultaneously. In this assessment, we rank all (term, protein) pairs according to their predicted probability and then plot the resulting precision-recall curve. Figure 11 shows the resulting curves for all twelve methods for each of the three ontologies.

These plots are difficult to interpret, both because they contain many series and because the series cross one another so frequently. We therefore used a bootstrap analysis (Boot strap procedure for joint evaluation section in Materials and methods) to evaluate the statistical significance of differences between these curves. The resulting tests give rise to win-loss scores, which are summarized in the right-most four columns of Figures 8 and 9.

Perhaps the most striking difference between the win-loss scores for the joint evaluation, as compared with the win-loss scores for the other two evaluations, is the variation in performance as we examine different recall values. In the previous two evaluation modes, a method generally performed well across all recall levels or poorly across all recall levels. This is not the case for joint evaluation. Here, some methods, such as BPAL, perform very poorly at low recall but very well at high recall. This change in performance arises because well calibrated methods can concentrate their best predictions on the left-most part of the precision-recall curve, leading to higher precision at low recall; however, the performance of these high precision methods at high recall can be quite different, and in that regime they can be significantly outperformed by other methods. An example is KLP, which obtains very high precision at low recall (R = 1%, 10%) and outperforms significantly, in that regime, both logistic regression and isotonic regression, according to our bootstrap tests (Additional data file 2). KLP also has higher win-loss scores at 1% recall than isotonic regression. For intermediate recall values, isotonic regression is again typically the best performing method, with higher win-loss scores than all other methods, both on average and individually for each group of terms, with the exception of the group of smaller molecular function and cellular components terms, for which KLP is better.

### Intermediate methods

In an attempt to understand what factors influence the performance of two important methods, belief propagation (BPAL) and the logistic regression, we considered several methods that are variants or intermediates between these two. In doing so, we wanted to separate the gain or loss in performance due to different aspects of Bayesian modeling from the gain or loss due to exploiting the constraints of the ontology.

First we considered independent naïve Bayes classifiers that use the same asymmetric Laplace likelihoods as BPAL but with independent priors for each term, in contrast to the general directed acyclic graph that BPAL uses as a structured prior. We did not describe these naïve Bayes results above because this is not a reconciliation method. However, its performance in these experiments (not shown) is informative. Naïve Bayes typically performs worse than unreconciled logistic regression but better than BPAL. This observation indicates that both of these Bayesian methods are worse than a discriminative method, and that BPAL suffers most from the arbitrary directed acyclic graph prior.

The other two intermediate methods that we considered have already been described: BPLR and BPLRf. These methods arise from the following considerations. The algorithm that we use to perform the Bayesian inference in BPAL is a variational algorithm, which is described in Additional data file 1. This variational formulation minimizes a Kullback-Leibler divergence that is slightly different from that used in KLP. However, the bigger difference between these two methods is that the former uses a structured prior and models the evidence with a likelihood term, both of which are subjective, whereas KLP uses the discriminative predictions of the logistic regression. In KLP, the log-odds for each node is the ratio of the logistic regression conditional probabilities, but in BPAL this log-odds is a ratio of likelihoods weighted by the prior. In the hybrid BPLR method, the latter is substituted by the log-odds ratio of the KLP. BPLR and BPLRf typically perform much better than BPAL, which demonstrates the advantage of using discriminatively estimated likelihood ratios. However, the hybrid methods typically perform worse than KLP, probably because the formulation of BPLR uses another term that contains the Bayesian prior.

### Isotonic regression performs well overall

Among the 11 reconciliation methods that we considered, the only one that is consistently among the winning methods for all three evaluation modes is isotonic regression. Furthermore, considering both the held-out set and the test set, all modes of evaluation, all three ontologies, all four term sizes, and all recall levels, isotonic regression consistently achieves a higher win-loss score than the unreconciled logistic regression estimates, except in 4 cases out of 360. It should be noted that, even if the improvement is not significant for individual tests (that heavily correct for multiple testing errors on the complete graph of twelve nodes), all the projections (isotonic regression, KLP, and KLPf) systematically improve over the logistic regression in the per-protein evaluation for each term size group and each recall level and in most of the configurations for the two other modes of evaluation. Furthermore, per protein, the improvement of isotonic regression is significant at all recall levels for the held-out set and at 1% recall on the test set. In addition, at high recall per protein (80%), isotonic regression improves significantly over logistic regression in both the held-out and test sets. In the per-protein evaluation, isotonic regression is significantly better than all other reconciliation methods on the biological process ontology, and better than the non-projection methods for the molecular function ontology at recall levels up to 50%.

**Figure 12:**
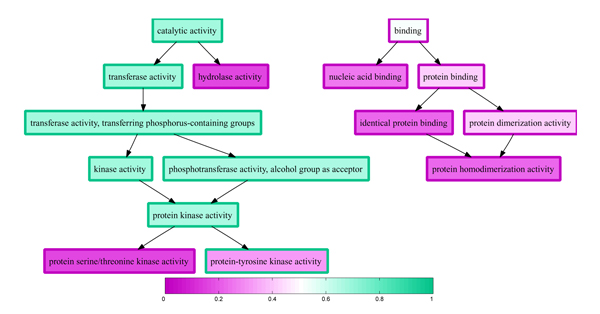
Predictions for the *ε *domain of casein kinase 1 reconciled by isotonic regression. The figure is the same as Figure 3, but the predicted probabilities have been reconciled by isotonic regression. The graph shows a portion of the molecular function GO, corresponding to positive labels and labels predicted with probability larger than 0.1. Each term's outline is colored according to the label with respect to the target term 'protein-tyrosine kinase activity'. A green outline corresponds to a positive label, and a purple outline corresponds to a negative label. The interior of each node is colored following a similar color scheme (green = 1 and purple = 0), according to the predicted probability.

Figure 12 illustrates the effect of applying isotonic regression to the logistic regression estimates shown in Figure 3. The single false positive prediction ('protein homodimerization activity') has been corrected, and two of the three false negative annotations ('catalytic activity' and 'transferase activity') have also been corrected. The remaining false negative ('protein-tyrosine kinase activity') has no children in the term set and, hence, would not be corrected by any reconciliation method.

### Small terms

The group of smallest terms deserves a specific analysis, because it is an important one; these terms represent 50% of all terms 'of interest' selected by Peña-Castillo and coworkers [1], and they also are the most specific and, hence, frequently the hardest to predict. However, for these smaller terms, the relative performance of the methods in our study is more variable, and as a consequence, we observe fewer statistically significant differences in performance. Furthermore, many of the differences apparent in the hold-out set are not supported by the results on the test set. Considering only the smallest terms, the per term evaluation does not discriminate among five reconciliation methods ('And,' 'Or,' isotonic regression, and the two Kullback-Leibler projections). In the per protein evaluation, 'And' does poorly on the hold-out set and well on the test set, and conversely isotonic regression and 'Or' do well on the hold-out set and poorly on the test set. Thus, for the per-term evaluation, only the two Kullback-Leibler projection methods perform well in both of these evaluation modes. It should be noted also that in the per-protein evaluation, KLP does not perform well on the held-out set but performs systematically well on small terms on the test set, with significant improvement over logistic regression. Finally, KLP also performs well for small terms under the joint evaluation mode.

### Joint evaluation at very high precision

**Figure 13:**
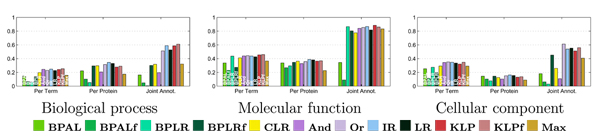
All three evaluation modes at fixed recall. Each panel plots the precision achieved at recall = 10% across the three different evaluation modes. The bars are colored according to the 12 methods, as described in Figure 4.

One of the motivations for considering the joint annotation regime is that the precision of the prediction almost doubles at low recall compared with the two other modes of evaluation. This effect is illustrated in Figure 13, which compares the recall achieved at 10% precision across all 12 methods and all 3 evaluation modes. The figure shows that, for the held-out set at recall = 10%, the precision levels jump from approximately 20% to 35% for biological processes, approximately 40% for molecular function and approximately 15% to 35% for cellular component in the per-term and per-protein evaluations to 60%, 80% and 55%, respectively, in the joint evaluation. Thus, a labeling that is adaptively parsimonious can improve the precision considerably. It is important to note that, in this regime, which corresponds to the high precision/low recall regime for the joint annotation evaluation, isotonic regression does not perform particularly well. In spite of the fact that the isotonic regression improves, on average, over logistic regression at low recall, it is often outperformed by the Kullback-Leibler projection. The latter is indeed significantly better than isotonic regression, on average, over the biological process ontology and the molecular function ontology at precision and recall less than 10% both on the held-out and test sets.

## Discussion

Overall, our experiments suggest that, among the reconciliation methods that we considered, isotonic regression is the most generally useful. Across a range of evaluation modes, term sizes, ontologies and recall levels, isotonic regression yields consistently high precision. On the other hand, isotonic regression is not always best, and a biologist with a particular goal in mind may wish to consult our experiments to select the most appropriate method. For small terms, we suggest using Kullback-Leibler projections rather than isotonic regression. Several other specific cases might be of interest; for example, for joint annotation, if precision will be evaluated at high recall values, then BPAL yields very good performance.

One striking overall observation is that reconciliation can yield a decrease in performance; thus, beating unreconciled logistic regression is nontrivial. Intuitively, the structure of the GO seems to be quite informative, and a biologist examining graphs like the one in Figure 3 might expect that any reasonable reconciliation will lead to a performance improvement. This assumption turns out to be incorrect. In many cases, the reconciled probabilities have lower precision than the original, unreconciled probabilities. This can be explained by the fact that estimating well the degree of confidence associated with a prediction is much harder than deciding whether a prediction is roughly correct or not. Because reconciliation methods essentially combine all of the confidences (or the strength of the evidence) obtained at each node, those individual confidence values must be carefully estimated. We have argued that, for equal levels of precision, reconciled predictions are clearly preferable to unreconciled predictions, because they are not self-contradictory. Our results show that although some methods do quite poorly compared with unreconciled logistic regression, we can essentially always do as well or better using one of the projection methods. Hence, it is never necessary to weigh the advantage of reconciled predictions against the disadvantage of a loss in precision due to reconciliation.

In terms of complexity and ease of implementation several of these methods are fairly comparable. The fastest methods are the naïve methods 'Max,' 'And,' and 'Or,' whose complexity is at worst O(*hn*), where *n *is the number of nodes and *h *is the height of the graph. The complexity of the cascaded logistic regression is the same as that of the logistic regression. Also, the complexity of performing the inference in the Bayesian network or the Kullback-Leibler projection is the same; both are iterative methods, and each iteration has a complexity equal to that of the naïve methods. An exact algorithm for isotonic regression is O(*n*^4^); however, the approximation that we employ is an iterative algorithm with iterations of complexity O(*hm*), where *h *is the height of the graph and *m *is the number of edges. For the GO, the height of the ontology graphs is small, *h *≤ 12, and, moreover, the number of parents is at most 6 so that *m *≤ 6*n*. This reduces significantly the complexity of most of the algorithms: the complexity of each iteration for the Bayesian inference or the projection is O(*n*). In practice, the iterative algorithm for the isotonic regression seemed to require slightly more iterations to converge than the projection algorithm. The projection algorithms as well as the inference in the Bayesian network are typically executed in a few minutes on the entire training set.

One of the advantages of logistic regression is that it is trained discriminatively, in a way that directly optimizes the decision function, which models a probability for the binary term variable *Y*_*i *_given the evidence *X*_*i*_. In comparison, a Bayesian network models the same conditional probability only indirectly, through the likelihood of the evidence *X*_*i *_given *Y*_*i*_. Moreover, the logistic regression explicitly optimizes the 'calibration' of the decision function, that is, how well the probabilistic values returned by the algorithm match the actual empirical success of correct prediction. In contrast, the probabilistic values returned by a Bayesian algorithm depend not only on the prior but crucially on the accuracy of the likelihood, which typically is very far from being a reasonable approximation of the actual underlying distribution. Because the logistic regressions are trained per term, it is not surprising that they perform better than many other methods in that regime.

A question often asked when 'probabilizing' GO, that is, treating it as a graphical model that encodes some conditional independencies between functions rather than just deterministic relations between terms, is which orientation of the edges of the graph is most reasonable. Whereas we see a big difference in performance when changing the orientation of the edges for the Bayesian network, the difference is much smaller in the case of Kullback-Leibler projection. It still seems, however, that generally a top-down parameterization leads to the best results.

Isotonic regression uses a similar notion of projection, but does not make probabilistic assumptions on the GO, only taking into account marginal probabilities for each term and deterministic implications between terms. The method's good performance might be explained by the fact that it makes fewer assumptions than other methods and corresponds to the smallest distortion of the logistic regression probabilities satisfying the deterministic constraints. We did not mention cascaded logistic regression, which has two weak points: that a prediction mistake somewhere in the cascade might be difficult to compensate for and that the conditional logistic regression might be difficult to estimate for lack of positive examples.

Finally, it is worth mentioning that in spite of being fairly simple, both the 'And' and 'Or' methods perform well in some situations. In particular, 'And' tends to perform better for smaller terms, whereas 'Or' performs better for larger terms. This observation is consistent with the semantics associated with these terms: for small terms, checking that all ancestral terms agree with the prediction is a good guarantee, and for larger terms, considering the evidence coming from the descendant term is relevant. It should be noted that 'And' and 'Or' are, in principle, very similar to cascaded logistic regression and its flipped variant, respectively.

Overall, this work aims to address three essential points. The first point concerns the goals of protein function prediction; we propose three distinct modes of evaluation, corresponding to three different uses of prediction methods. The second point concerns calibration - the estimation of a confidence level for prediction that is common to all classifiers. Although we have emphasized the need for calibration only in the joint annotation mode of evaluation, it should be clear that calibration is more generally desirable, regardless of the evaluation mode, because calibration leads to higher levels of precision. Finally, the third point concerns reconciliation. The main goal of a reconciliation is to obtain interpretable output. It is important, however, to assess the extent to which interpretability is obtained at a cost in terms of accuracy; indeed, as we have seen, several apparently reasonable reconciliation algorithms lead to a decrease in accuracy. Our results allay this concern - we have identified a class of projection methods that maintains accuracy while producing predictions that are consistent and, therefore, interpretable to the biologist.

## Materials and methods

### Kernels

The representation of the data used by a SVM is determined by the choice of a kernel function. For each data type, we train SVMs using three or more different kernel functions. Three are common to all data types.

The first kernel function common to all data types is the linear kernel:

*K*(*x*, *y*) = *x*·*y*

where *x *and *y *are standardized data vectors; that is, each variable in the data matrix is centered to have mean 0 and scaled to have variance 1.

The second is the normalized version $\tilde{K}$ of the linear kernel:

$$K:\tilde{K}(x,y)=K(x,y)/\sqrt{K(x,x)K(y,y)}$$

The third is the Gaussian kernel or radial basis function kernel:

$$K(x,y)=e^{-{\left\|x-y\right\|}^{2}/\lambda }$$

In addition, for two data types we compute a data-specific kernel. These kernels are described below.

For the protein-protein interaction data, we compute a diffusion kernel [6] on the graph of proteins connected by interactions. Diffusion kernels correspond to embeddings of the vertices in a Hilbert space, where the inner product between two vertices approximates the probability of traveling from one vertex to the other in time *τ *by random walk on the graph. Nodes connected by shorter paths therefore contribute more to the similarity. We compute three diffusion kernels with *τ *∈ {0.1, 1, 10}.

For the Zhang expression data, Zhang and coworkers [7] conclude from their study that patterns of co-expression across tissues are 'more predictive of function than tissue-specific expression levels.' To use the large amount of unlabeled data, we use a representation of genes in terms of co-expressed genes; consider the columns of the linear kernel matrix constructed from gene expression data as a new vector representation of the genes, and then compute a linear kernel from those vectors. This corresponds to squaring the original kernel matrix. Note that this is different from a Hadamard product of the matrix with itself corresponding to a quadratic kernel, which would not use unlabeled data.

We finally compute four kernels that are linear combinations of the previously described ones. More specifically, we consider two combinations of the data types with the largest coverage: PfamA, Interpro, Inparanoid, Biomart, Zhang, Su, SAGE; and PfamA, Interpro, Inparanoid, Biomart, PPI, Zhang, Su, SAGE (see Table 1 for a summary of these data types).

For each of these combinations, we renormalize the corresponding linear kernels (restricted to the set of genes they have in common) either by their trace or by the ratio of their trace to their Frobenius norm. This yields four additional kernels.

### SVM training

In the second stage of the pipeline shown in Figure 1, we train a collection of SVM classifiers for each GO term, building one SVM for each combination of kernel and GO term. Thus, the output of this stage is a matrix of SVM discriminant scores with three dimensions: gene, GO term, and kernel. Note that, typically, not all data types are available for a given gene, so that some of the entries in this matrix are missing. We handle these missing values in the subsequent stages.

For each SVM, proteins annotated with the target GO term or a descendant term are labeled as positive examples. Proteins annotated with an ancestor term are ignored. All remaining proteins are labeled as negative examples. Let the numbers of positive and negative examples be *n*^+ ^and *n*^-^, respectively. Each SVM is trained from a randomly selected 60% of the training points. The SVMs are trained using two soft-margin parameters *C*^+ ^and *C*^-^, penalizing, respectively, false positives and false negatives, and chosen, respectively, proportional to 1/*n*^+ ^and 1/*n*^-^. For the third stage, we need samples of the distribution of SVM outputs on new data. To simulate these samples from the training data, we use fivefold cross-validation, and we store all evaluations on held-out data for use during the third stage. The discriminant function used on any new gene is the average of the five discriminants from the cross-validation.

### Missing data

In the third step of Figure 1, SVM outputs are either mapped to probabilistic outputs using a logistic regression or, if the fourth step is Bayesian inference, to their likelihood in a mixture of fitted asymmetric Laplace distributions. For the Bayesian network, we use the approach of Barutcuoglu and coworkers [3], except that we use asymmetric Laplace distributions and a variational inference algorithm described in Additional data file 1, rather than exact inference. In the Bayesian network framework, data types are considered conditionally independent given the GO term assignments, and missing values are, therefore, accommodated naturally. We refer the reader to Bennett [8] for details of the likelihood parameter estimation.

Missing data are more problematic for the logistic regression. Each protein exhibits a certain pattern of missing data across the ten data types. This pattern can be represented as a bit string of length 10. In practice, most patterns occur very rarely; therefore, we consider only the 15 most frequent patterns. Each protein is associated with the pattern most similar to its own, provided there is at most one data type difference between their data type patterns. Otherwise, it is considered an orphan. When one data type is actually missing in the protein pattern compared with its group pattern, we fill in the corresponding value with the average value of the SVM output for that type. For each of the 15 groups, we then fit an unregularized logistic regression, whose output on the test set gives a confidence measure for the prediction. A few examples, the orphans, are not matched to any clusters; for these, first, individual logistic regressions are learned from the output of each kernel specific SVM using the training data; then those logistic regressions are evaluated on the orphan points if they have the corresponding data type. Finally, for each orphan point we average the output of the logistic regressions corresponding to the data types available and use the obtained probability as a confidence measure for the prediction.

### Logistic regression and naive Bayes

To investigate the loss of precision incurred specifically by modeling SVM outputs in a generative fashion as in the Bayesian network, we consider the unstructured counterpart of the Bayesian network that treats each term independently. For each term, this is a naïve Bayes model based on asymmetric Laplace likelihoods (we use the NBAL acronym for this method). The SVM outputs are modeled as in the Bayesian network and the marginal frequency of terms is used as a prior for each of them. The probabilistic value returned is the posterior probability obtained by Bayes rule.

### Statistical testing

From the point of view of testing whether a method is significantly more precise at fixed recall than another, we use a Z-test on the difference of average precisions, which measures the average amount of improvement from one method to the next. This test is appropriate if the number of observations is large enough that the average can be assumed approximately distributed as a Gaussian variable. This is the case both in per-term and per-protein evaluations, where there are typically several hundreds of proteins or terms and in any case no less than 35.

To perform simultaneous comparisons of different methods with Z-tests, we determine first a 95% ellipsoidal confidence region for the vector of means of the methods we are comparing, and then we ask for which pairs of methods (*k*, *l*) the ellipsoid is entirely on one side of the hyperplane with equation *x*_*k *_= *x*_*l*_. The latter question matches the Gaussian rejection region for the one dimensional statistic *T *defined as the difference of average precisions for methods *k *and *l *renormalized by the empirical standard deviation of the difference. In the tests reported in the Results section, we report the Gaussian *p*-value for *T*.

### Bootstrap procedure for joint evaluation

The two sample Z-tests used for the per-protein and per-term evaluations are based on observations corresponding to the value of the precision for different terms at a fixed recall value. In the case of the joint evaluation, we *a priori *get a single precision value for a fixed recall instead of a whole sample of such precision values. To obtain a whole sample, we should be allowed to draw several test (or held-out) sets from a larger distribution of known proteins. The theory of the bootstrap is based on the idea that the empirical distribution of the sample is in itself a good approximation of the distribution that it is sampled from, and that, therefore, an estimator of a function of the distribution can be obtained by plugging the sample empirical distribution into the function. Applying the concept of the bootstrap to our situation, we can estimate the distribution of precision values at a fixed recall value by constructing repeatedly precision-recall curves from bootstrap samples and computing the precision at that fixed recall. In particular, the mean of that distribution approximates the mean precision-recall curve of a sample. If the precision-recall curve for several methods are bootstrapped based on the same bootstrap sample, then the probability that one method beats another can be estimated or tested. We perform multiple tests to find pairs of methods (A, B) such that A outperforms B with probability 0.9. The result of the tests based on 315 bootstrap samples can be shown to guarantee a confidence level higher than 95%, after correction for multiple testing.

## Abbreviations

BPAL, belief propagation with asymmetric Laplace; BPLR, belief propagation with logistic regression; BPLRf, flipped variant of BPLR; GO, Gene Ontology; KLP, Kullback-Leibler projection; KLPf, KLP with edges flipped; SVM, support vector machine.

## Competing interests

The authors declare that they have no competing interests.

## Authors' contributions

GO, GL, MIJ, and WSN conceived and designed the experiments. GO implemented the methods. GO and CEG ran the experiments. GO, GL, MIJ, and WSN wrote the manuscript.

## Additional data files

The following additional data are available with the online version of this paper. Additional data file 1 provides a detailed description of the 11 reconciliation methods used in this study. Additional data file 2 includes a systematic set of figures summarizing the performance of all 12 methods across all three evaluation modes, four term sizes and four recall levels.

## Supplementary Material

Additional data file 1 A detailed description of the 11 reconciliation methods used in this study. Click here for file

Additional data file 2 A systematic set of figures summarizing the performance of all 12 methods across all three evaluation modes, four term sizes, and four recall levels. Click here for file
