# Supplementary material for: Consistent probabilistic outputs for protein function prediction
Source: Genome Biol. 2008 Jun 27;9(Suppl 1):S6. doi: 10.1186/gb-2008-9-s1-s6 (PMC2447540; doi:10.1186/gb-2008-9-s1-s6)
Supplement: Additional data file 1 — A detailed description of the 11 reconciliation methods used in this study. [file gb-2008-9-s1-s6-S1.pdf]

Technical supplement to "Consistent probabilistic outputs for protein function prediction"

Guillaume Obozinski
Department of Statistics
UC Berkeley
Berkeley, CA, USA

Gert Lanckriet
Department of Electrical and Computer Engineering
UC San Diego
San Diego, CA, USA

Charles Grant
Department of Genome Sciences
University of Washington
Seattle, WA, USA

Michael I. Jordan
Department of Statistics
UC Berkeley
Berkeley, CA, USA

William Stafford Noble
Department of Genome Sciences
Department of Computer Science and Engineering
University of Washington
Seattle, WA, USA

Protein function prediction, in the context of the Gene Ontology, is a task that consists of answering, for a fixed protein X, a large number of binary questions of the form: "Does protein X belong to GO term Y?" Those binary classification problems are strongly related because the ontology consists of nested classes. Two natural requirements for this prediction problem are

- that the set of predictions be *consistent*, i.e., that if a protein is assigned a GO term, then it is all also assigned all the ancester GO terms, and
- that high-confidence predictions can be produced with a quantified confidence level.

Methods of structured classification proposed in machine learning Taskar et al. [2003] could in theory be used to tackle this problem. However, two practical difficulties that need to be surmounted are the large amount of missing data and the large scale of the

problem. To address both issues, our approach consists of first making partial predictions for each term given each data type and then integrating all partial predictions to form a set of consistent predictions whose confidence level can be estimated. We use *calibration* methods to assign a confidence level to partial predictions and subsequently consider several *reconciliation* methods to produce, for the whole ontology, a set of confidence levels that are *consistent* in the sense that they naturally yield consistent predictions.

The presentation of the methods is organized as follows: in Sec. 1 the notions of calibration as it applies to our problem, the algorithm we use to obtain calibrated partial predictions and generally relevant notations are introduced. In Sec. 2 the Bayesian formulations as well as an efficient variational formulation to perform inference are presented. In Sec. 4, we present algorithms based on projections of probability distributions.

1 Calibration

In statistics, the calibration of a prediction for the binary variable Y is a procedure which, given some evidence X, returns a probability value in $[0,1]$ that reflects the confidence that the predicted value is $Y{=}1$. From a Bayesian point of view a natural candidate is $\mathbb{P}\left(Y|X\right)$, as obtained by Bayes' rule, where X is some input variable that the prediction is based on. Frequentists, on the other hand, define *well-calibrated* predictions as those that are good estimates of their own success probability. Formally, S is a well-calibrated prediction if $\mathbb{P}\left(Y=1|S=p\right)=p$. We refer the reader to the relevant literature Cohen and Goldszmidt [2004]. The simplest frequentist method for calibration is the logistic regression, which as suggested by Platt [1999] , can be used in combination with support vector machines (SVMs) [Boser et al., 1992].

In the present work we are interested in calibrating jointly several related GO term predictions. Extending the Bayesian approach to this structured problem is fairly natural and has been explored for function prediction by Barutcuoglu et al. [2006]. Similarly, logistic regression can be extended to the structured case by conditional random fields (CRFs). We pursue yet another direction, in which the calibrated values obtained from individual logistic regressions are combined to yield a set of consistent calibrated values. This type of problem has been studied in the multiclass classification setting, where several class specific binary classifiers are combined to predict a set of mutually exclusive classes Wu et al. [2003]. An advantage of this approach is that the missing data are dealt with on a per term basis, which is easier.

1.1 Constrained calibration and notations

Whereas the structure that is generally exploited in the multiclass setting is that classes are mutually exclusive, here we wish to exploit the *inclusion* relations between GO terms. The consequence of such structure on calibrated values is that confidence should decrease along any lineage of the ontology. More formally, if in the ontology G, there is an edge $i \rightarrow j$ corresponding to GO term i being a parent of GO term j, by which we mean that GO term j is included in GO term i, and if $(p_i)_{i\in I}$ are confidence values, then we should have $p_i \geq p_j$. Notice that the ontology graph then defines a partial order on $(p_i)_{i\in I}$. This

can be interpreted simply as the fact that confidence should decrease as one makes more precise predictions; but because ideal calibrated values p_i can be interpreted as probabilities, one can further consider them as originating from a joint probability distribution P on a set of binary random variables $\mathbf{Y} = (Y_i)_{i\in I}$ that are indicators for each term, such that $p_i = P(Y_i = 1)$ and, finally, such that for all edges $i \to j$ the implication $(Y_j = 1) \Rightarrow (Y_i = 1)$ translates in probabilistic terms as $P(Y_i = 0, Y_j = 1) = 0$. The set of distributions satisfying the implication (or inclusion) constraints of the graph is then

$$\mathcal{P}^{\Rightarrow} = \{\, P \in \mathcal{P} \,|\, \forall (i,j) \in E, \quad P(Y_i = 0, Y_j = 1) = 0 \,\},$$

which is a subset[1] of $\mathcal{P}^{\leq} = \{\, P \in \mathcal{P} \,|\, \forall (i,j) \in E,\ p_j \leq p_i \,\}$.

A subset that is easier to parameterize is the set of distributions in $\mathcal{P}^{\Rightarrow}$ that factorize according to the graph G. To formally define this set, we introduce some notation that we will use throughout this appendix. Denote by π_i, c_i, A_i and D_i respectively the set of parents, children, ancesters and descendants of a node i in G and denote by $Y_{\pi_i} = \prod_{j\in\pi_i} Y_j$ as well as $y_{\pi_i} = \prod_{j\in\pi_i} y_j$ where $y_j \in \{0,1\}$ is the value of a realization of Y_j. The distributions that factorize according to G can be defined formally as

$$\mathcal{P}^G = \left\{ P \in \mathcal{P} \,|\, P(\mathbf{Y} = \mathbf{y}) = \prod_{i\in I} P(Y_i = y_i | Y_{\pi_i} = y_{\pi_i});\ \forall i, P(Y_i = 1 | Y_{\pi_i} = 0) = 0 \right\}.$$

An element of $\mathcal{P}^G$ is completely characterized by its set of conditional distributions: $q_i = \mathbb{P}\left(Y_i = 1 | Y_{\pi_i} = 1\right)$. The marginal probabilities (our calibrated values) can then be computed immediately from the the conditionals:

$$p_i = P(Y_i = 1) = q_i p_{\pi_i} \quad \text{where} \quad p_{\pi_i} = P(Y_{\pi_i} = 1) = \prod_{j\in A_i} q_j$$

and vice versa: the conditionals $q_i = \frac{p_i}{p_{\pi_i}}$ are easy to obtain from the marginals. Several of the methods we consider require computing the entropy of the distribution. For distributions in $\mathcal{P}^G$, the entropy has a simple analytical expression:

$$H(P) = H(\mathbf{Y}) = \sum_{i\in I} H(Y_i | Y_{\pi_i}) = \sum_{i\in I} H(Y_i | y_{\pi_i} = 1) \mathbb{P}(Y_{\pi_i} = 1) = \sum_{i\in I} h(q_i) p_{\pi_i}$$

with h the binary entropy function defined by $h(x) = x\log x + (1-x)\log(1-x)$. Finally, we can consider the graph G^{-1} that inverts the parent-children relationships in G i.e. $(i \to j \in G) \Leftrightarrow (i \leftarrow j \in G^{-1})$. Another set of distributions factorizes according to G^{-1}. If we define $Y_{c_i} = \max_{j\in c_i} Y_j$, and similarly for y_{c_i}, then that set can be written as

$$\mathcal{P}^{G^{-1}} = \left\{ P \in \mathcal{P} \,|\, P(\mathbf{Y} = \mathbf{y}) = \prod_{i\in I} P(Y_i = y_i | Y_{c_i} = y_{c_i});\ \forall i, P(Y_i = 0 | Y_{c_i} = 1) = 0 \right\}$$

We omit further details on $\mathcal{P}^{G^{-1}}$ here.

[1] Although both sets $\mathcal{P}^{\Rightarrow}$ and $\mathcal{P}^{\leq}$ have the same marginals.

2 The Bayesian approach

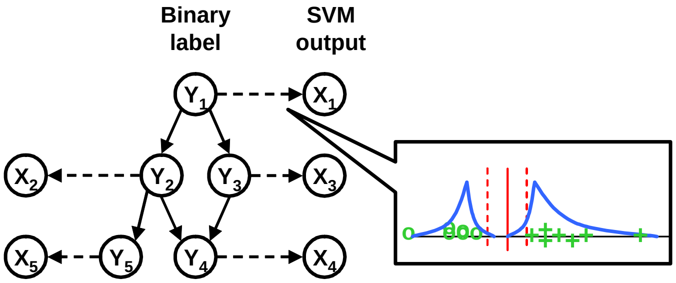

The principle of the Bayesian approach is to turn the decision problem "Does protein j have function i?" into an inference problem. The answer to the question is encoded as a binary number which is treated as a random variable. Initially, a prior joint distribution for the binary Y_i variables is chosen, and one assumes that given $Y_i = y_i$ some evidence x_i is observed independently for each GO term according to $p(x_i|Y_i = y_i) = L_i(y_i)$, $y_i \in \{0, 1\}$. Subsequently, using Bayes' rule and appropriate computational methods the quantity $P(\mathbf{Y}|\mathbf{X} = \mathbf{x})$ is computed and used as the calibrated value. In the approach of Barutcuoglu et al. [2006], the evidence is the output of an SVM classifier. It is natural to choose the prior P_0 in $\mathcal{P}^G$ or $\mathcal{P}^{G^{-1}}$. If $P_0 \in \mathcal{P}^G$ then $P_0(\mathbf{Y} = \mathbf{y}) = \prod_{i\in I} q_{i0}^{y_i y_{\pi_i}} (1 - q_{i0})^{y_{\pi_i} - y_i} \mathbf{1}_{\{y_i \le y_{\pi_i}\}}$, and the posterior distribution is

$$P(\mathbf{Y} = y|\mathbf{X} = \mathbf{x}) = \frac{1}{Z} \prod_{i\in I} q_{i0}^{y_i y_{\pi_i}} (1 - q_{i0})^{y_{\pi_i} - y_i} L_i(1)^{y_i} L_i(0)^{1-y_i} \tag{1}$$

Unless G is a tree, the posterior distribution is not in $\mathcal{P}^G$ (or respectively in $\mathcal{P}^{G^{-1}}$ if the prior was a tree). The Bayesian formulation takes us naturally outside of the class of models considered. A computational consequence is that, except in the tree case, calculating the marginal probabilities, i.e., performing exact inference, becomes expensive, and approximate inference is necessary. In Barutcuoglu et al. [2006], the authors use exact inference because they limit their analysis to small graphs (personal communication). Approximate inference can typically be performed using loopy belief propagation or some other variational method [Wainwright and Jordan, 2003]. We present next an efficient variational inference algorithm that fits well within our framework.

2.1 Variational inference in $\mathcal{P}^G$

The principle of variational inference is to write an optimization problem whose minimizer is the set of marginal probabilities of the distribution, when the unnormalized exponential form of that distribution is available, and use optimization algorithms to solve for the minimum [Wainwright and Jordan, 2003]. Typically, if the inference involves finding the marginals, say $\mathbf{m}$, of a distribution $Q \in \mathcal{P}$, where the latter is some exponential family, then the unnormalized log-likelihood is a dot product $\langle\theta, \phi(\mathbf{y})\rangle$ between the vector of parameters θ and some vector of sufficient statistics $\phi(\mathbf{y})$ such that $\mathbf{m} = \mathbb{E}\,\phi(\mathbf{Y})$, and the entropy of the distribution can be written as a function of $\mathbf{m}$: $H(\mathbf{m})$. To a given $\mathcal{P}$ corresponds a set of

possible marginals $\mathcal{M}$. The variational inference problem can then be formulated as the optimization problem:

$$\max_{\mathbf{m}\in\mathcal{M}} H(\mathbf{m}) + \langle \mathbf{m}, \theta(\mathbf{x})\rangle$$

In the Bayesian inference situation presented above, taking the log of (1) we get the log of the unnormalized log-likelihood and identify $\langle \phi(\mathbf{y}), \theta\rangle =$

$$\sum_i \langle \phi_i(y_i, y_{\pi_i}),\, \theta_i(x_i)\rangle = y_i y_{\pi_i}\eta_{i1} + (1-y_i) y_{\pi_i}\eta_{i0} + y_i\ell_i(1) + (1-y_i)\ell_i(0) \tag{2}$$

with, to match (1), $\eta_{i1} = \log q_{i0}$, $\eta_{i0} = \log(1-q_{i0})$ and $\ell_i(k) = \log L_i(k)$.

Taking expectations of (2) with respect to any distribution in $\mathcal{P}^{\Rightarrow}$ we get

$$\langle \mathbf{m}, \theta\rangle = \sum_i \langle m_i,\, \theta_i\rangle = p_i\eta_{i1} + (p_{\pi_i} - p_i)\eta_{i0} + p_i\ell_i(1) + (1-p_i)\ell_i(0) \tag{3}$$

At this point, we still have to define the set of possible marginals. One of the difficulties here is that the posterior distribution whose marginal we are after is in $\mathcal{P}^{\Rightarrow}$, but it is in general not in $\mathcal{P}^{G}$. If we denote by $\mathcal{M}^{\Rightarrow}$ (resp. $\mathcal{M}^{G}$) the set of marginals obtainable from joint distributions in $\mathcal{P}^{\Rightarrow}$ (resp. $\mathcal{P}^{G}$), then we can write our optimization problem as

$$\max_{\mathbf{m}\in\mathcal{M}^{\Rightarrow}} H(\mathbf{m}) + \langle \mathbf{m}, \theta\rangle$$

One typically appeals to variational inference in cases where the optimization problem is intractable. An approximate variational inference method, instead of finding the exact set of marginals, finds the closest set of marginals in a simpler distribution class. It turns out that $\mathcal{M}^{\Rightarrow}$ is not easy to deal with: in particular the expression of $H(\mathbf{m})$ for $\mathbf{m} \in \mathcal{M}^{\Rightarrow}$ is in general intractable. By contrast, $\mathcal{P}^{G}$ is an easier distribution class for which entropy is computed easily as we argued in Sec. 1.1. Therefore, we consider the approximate inference problem

$$\max_{\mathbf{m}\in\mathcal{M}^{G}} H(\mathbf{m}) + \langle \mathbf{m}, \theta\rangle$$

Parametrizing this optimization problem with the conditionals q_i and setting the gradient to 0 we get the following fixed point equations:

$$\log\frac{q_i}{1-q_i} \;=\; f_i + \sum_{k\in D_i}[\, f_k q_k + \eta_{i0} + h(q_k)\,]\frac{p_{\pi_k}}{p_i} \tag{4}$$

with $f_i = \eta_{i1} - \eta_{i0} + \ell_i(1) - \ell_i(0)$. Note that, with the notations of (1), if we define $\tilde{q}_i$ through a simple Bayes rule, we obtain $\frac{\tilde{q}_i}{1-\tilde{q}_i} = \frac{q_{i0}L_i(1)}{(1-q_{i0})L_i(0)}$ then $f_i = \log\frac{\tilde{q}_i}{1-\tilde{q}_i}$. This suggest a modification of the BPAL algorithm where this log-odds ratio is set with the output of a logistic regression using $\tilde{q}_i = \hat{p}_i$; we call that algorithm BPLR. Notice also that the equation for q_i given the other variables (4) is in closed form because q_i cancels in the numerator and denominator of $\frac{p_{\pi_k}}{p_i} = \prod_{j\in A_k\setminus(A_i\cup\{i\})} q_j$. Because the function considered is strictly concave with respect to each q_i, enforcing the fixed point equation iteratively on the coordinates performs coordinate ascent on the function and therefore converges to a local maximum.

3 Cascaded logistic regression

A major weakness of the Bayesian approach is that it requires the models for conditional densities $p(x_i|Y_i=y_i)$ to be nearly correct, which is in general unlikely to be the case. In other words, the method is not robust to model specification. The advantage of logistic regression is precisely that it models $P(Y_i=y_i|X_i=x_i)$ directly. A way to construct a model that approximates $P(\mathbf{Y}=\mathbf{y}|\mathbf{X}=\mathbf{x})$ in the case where the dependencies between terms are taken into account is as follows. Assume that $P(\mathbf{Y}=\mathbf{y}|\mathbf{X}=\mathbf{x})$ is in $\mathcal{P}^G$ so that it factorizes according to the graph. This implies that

$$P(\mathbf{Y}=\mathbf{y}|\mathbf{X}=\mathbf{x}) = \prod_i P(Y_i=y_i|Y_{\pi_i}=y_{\pi_i}, X_i=x_i)$$

with $P(Y_i=1|Y_{\pi_i}=0, X_i=x_i) = 0$. A natural candidate for $P(Y_i=1|Y_{\pi_i}=1, X_i=x_i)$ is then a logistic regression. Fitting this model is very similar to fitting independent logistic regressions except that only examples of proteins having all parents GO terms are used to fit the model. That is also a weakness of that model: some training sets have very few negative examples.

4 Projection methods

The methods presented so far to reconcile the predictions for different GO terms either appeal to a generative model (e.g., the model of Barutcuoglu et al. [2006]) and model the distribution of SVM outputs by some mixture of densities, which is quite far from optimal from a calibration point of view (naive Bayes from the same densities is inferior to logistic regression) or appeal to the more sophisticated machinery of CRFs, which are difficult to implement and require a further step of learning.

In contrast, in this section, we propose methods that make direct use of the calibrated values obtained from the logistic regressions and try to find the closest set of values that are consistent with the ontology.

4.1 Isotonic regression

Denote by $(\hat{p}_i)_{i\in I}$ the set of calibrated values obtained from the logistic regressions. We propose to find a set of marginal probabilities $(p_i)_{i\in I}$ in $\mathcal{M}^{\Rightarrow}$ such that $\hat{p}_i$ and p_i are close together. A first formulation could consist in choosing the ℓ_2 distance as a measure of closeness. This approach yields the quadratic program (QP)

$$\begin{aligned} \min_{p_i,\, i\in I} \quad & \sum_{i\in I}(p_i-\hat{p}_i)^2 \\ \text{s.t.} \quad & p_j \le p_i, \quad (i,j)\in E \end{aligned} \tag{IR}$$

This QP is known in the statistical literature as the isotonic regression problem. When the inequality constraints correspond to a total order, then the problem is simpler, and an efficient algorithm, PAVA [Barlow et al., 1972], is known to solve it. More generally, isotonic

regression can be solved using an interior-point solver, provided the number of edges in the graph is not too large. An approximate algorithm with complexity $\mathcal{O}(n^2)$ was recently proposed by Burdakov et al. [2006]. We use a simple algorithm based on iterations of PAVA which approximates the solution. Instead of using the Euclidian distance, we consider the Kullback-Leibler divergence $D(p_i \| q_i) = p_i \log \frac{p_i}{q_i} + (1-p_i)\log\frac{1-p_i}{1-q_i}$, which is more natural for probability distributions. There are two ways to minimize simultaneously all term specific KL-divergences, because the latter is not symmetric.

$$\begin{array}{ll} \min\limits_{p_i,\, i\in I} & \sum\limits_i D(\hat{p}_i \| p_i) \\ \text{s.t.} & p_j \le p_i, \quad (i,j)\in E \end{array} \quad \text{(IR.2)} \quad \text{or} \quad \begin{array}{ll} \min\limits_{p_i,\, i\in I} & \sum\limits_i D(p_i \| \hat{p}_i) \\ \text{s.t.} & p_j \le p_i, \quad (i,j)\in E \end{array} \quad \text{(LO-IR)}$$

These two problems turn out to be closely related to the previous one: the solutions to (IR) and (IR.2) are actually the same. The KKT conditions of (LO-IR) show that its solution can be obtained by solving an isotonic regression on the log-odds ratios $\log\frac{\hat{p}_i}{1-\hat{p}_i}$ with the same inequalities and then mapping the obtained log-odds ratios back to probabilities.

4.2 Projections on $\mathcal{P}^G$

Note that in the previous section, even though we minimize a sum of KL-divergences between pairs of marginals, we are actually not minimizing a KL-divergence between joint distributions. Another way of formulating a projection is as follows: define a joint distribution $\hat{P}$ on the GO terms, where each individual term is independently Bernoulli distributed $Ber(\hat{p}_i)$ and find a joint distribution P which is close and satisfies the constraints, i.e., $P \in \mathcal{P}^{\Rightarrow}$. For instance, the problem can be stated[2] as $\min D(P\|\hat{P}) \quad \text{s.t.} \quad P\in\mathcal{P}^{\Rightarrow}$.

However, a generic distribution from $\mathcal{P}^{\Rightarrow}$ is not tractable for the reasons outlined previously. On the other hand, if we consider $\mathcal{P}^G$, then the problem becomes tractable. Using a parameterization with conditional distributions q_i the problem can be written as:

$$\begin{aligned} \min_{P\in\mathcal{P}^G} D(P\|\hat{P}) &= \min_{P\in\mathcal{P}^G} \sum_{i\in I} \mathbb{E}_P[X_i \log\hat{p}_i + (1-X_i)\log(1-\hat{p}_i)] + H(P) \\ &= \min_{q_i\in[0,1]^n} \sum_{i\in I} [p_i\log\hat{p}_i + (1-p_i)\log(1-\hat{p}_i) + h(q_i)p_i] \end{aligned}$$

with $p_i = \prod_{j\in A_i} q_j$. We differentiate the above expression to find a stationary point:

$$\frac{\partial}{\partial q_i} = p_{\pi_i}\log\frac{\hat{p}_i}{1-\hat{p}_i} - p_{\pi_i}\log\frac{q_i}{1-q_i} + \sum_{k\in D_i}\frac{\partial p_{\pi_k}}{\partial q_i}h(q_k) + \frac{\partial p_k}{\partial q_i}\log\frac{\hat{p}_k}{1-\hat{p}_k}$$

Because $\frac{\partial p_k}{\partial q_i} = \frac{p_k}{q_i} = \prod_{j\in A_k\setminus\{i\}} q_j$ does not depend on q_i, coordinate descent has the closed form updates

$$\log\frac{p_i}{1-p_i} = f_i + \sum_{k\in D_i}[H(q_k) + q_k f_k]\frac{p_{\pi_k}}{p_i} \tag{5}$$

with $f_i = \log\frac{\hat{p}_i}{1-\hat{p}_i}$. Notice that the update rules for (5) and (4) are quite similar.

[2] Notice that the symmetric formulation with $D(\hat{P}\|P)$ is excluded because it would require $P \ll \hat{P}$ which is not true for most $\hat{P}$

References

R.E. Barlow, D.J. Bartholomew, J.M. Bremner, and H.D. Brunk. *Statistical inference under order restrictions; the theory and application of isotonic regression*. Wiley, New-York, 1972. ISBN 0-471-04970-0.

Z. Barutcuoglu, R. E. Schapire, and O. G. Troyanskaya. Hierarchical multi-label prediction of gene function. *Bioinformatics*, 22(7):830–836, 2006.

B. E. Boser, I. M. Guyon, and V. N. Vapnik. A training algorithm for optimal margin classifiers. In D. Haussler, editor, *5th Annual ACM Workshop on COLT*, pages 144–152, Pittsburgh, PA, 1992. ACM Press.

O. Burdakov, O. Sysoev, A. Grimvall, and M. Hussian. An $\mathcal{O}(n^2)$ algorithm for isotonic regression. In G. Di Pillo and M. Roma, editors, *Large-Scale Nonlinear Optimization*, volume 83 of *Nonconvex Optimization and Its Applications, Springer-Verlag*, pages 25–33. Springer-Verlag, Berlin, 2006.

I. Cohen and M. Goldszmidt. Properties and benefits of calibrated classifiers, 2004. URL `citeseer.ist.psu.edu/cohen04properties.html`.

J. C. Platt. Probabilities for support vector machines. In A. Smola, P. Bartlett, B. Schölkopf, and D. Schuurmans, editors, *Advances in Large Margin Classifiers*, pages 61–74. MIT Press, 1999.

B. Taskar, C. Guestrin, and D. Koller. Max margin Markov networks. In S. Becker, S. Thrun, and K. Obermayer, editors, *Advances in Neural Information Processing Systems*, Cambridge, MA, 2003. MIT Press.

M. J. Wainwright and M. I. Jordan. Graphical models, exponential families, and variational inference. Technical Report 649, UC Berkeley, Dept. of Statistics, 2003.

T. Wu, C. Lin, and R. C. Weng. Probability estimates for multi-class classification by pairwise coupling. In S. Thrun, L. K. Saul, and B. Schölkopf, editors, *Advances in Neural Information Processing Systems*, volume 16, pages 529–532, Cambridge, MA, 2003. MIT Press.
