# Supplementary material for: Consistent probabilistic outputs for protein function prediction
Source: Genome Biol. 2008 Jun 27;9(Suppl 1):S6. doi: 10.1186/gb-2008-9-s1-s6 (PMC2447540; doi:10.1186/gb-2008-9-s1-s6)
Supplement: Additional data file 2 — A systematic set of figures summarizing the performance of all 12 methods across all three evaluation modes, four term sizes, and four recall levels. [file gb-2008-9-s1-s6-S2.pdf]

Supplementary figures for "Consistent probabilistic outputs for protein function prediction"

Guillaume Obozinski
Department of Statistics
UC Berkeley
Berkeley, CA, USA

Gert Lanckriet
Department of Electrical and Computer Engineering
UC San Diego
San Diego, CA, USA

Charles Grant
Department of Genome Sciences
University of Washington
Seattle, WA, USA

Michael I. Jordan
Department of Statistics
UC Berkeley
Berkeley, CA, USA

William Stafford Noble
Department of Genome Sciences
Department of Computer Science and Engineering
University of Washington
Seattle, WA, USA

Contents

1 Per term evaluation **3**
1.1 Bar plots by ontology . . . 3
1.2 Directed graphs by ontology . . . 5
1.3 Bar plots by ontology and term size . . . 6
1.4 Directed graphs by ontology and term size . . . 13

2 Per protein evaluation **15**
2.1 Bar plots by ontology . . . 15
2.2 Directed graphs by ontology . . . 17
2.3 Bar plots by ontology and term size . . . 18
2.4 Directed graphs by ontology and term size . . . 25

3 Joint annotation evaluation **28**
3.1 Precision-recall curves by ontology . . . 28
3.2 Directed graphs by ontology . . . 31
3.3 Precision-recall curves by ontology and term size . . . 32
3.4 Zoomed precision-recall curves (recall $< 10\%$) by ontology and term size . . . 38
3.5 Directed graphs by ontology and term size . . . 44

1 Per term evaluation

1.1 Bar plots by ontology

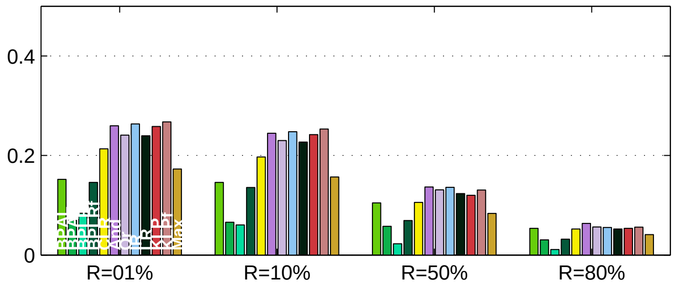

Figure S.1: **Proteins retrieved for a fixed GO term for the Biological Process ontology (hold-out set)** In average over GO terms with the same recall levels: precisions obtained by different methods for values of the recall respectively fixed at $R = 1\%$, $R = 10\%$, $R = 50\%$, $R = 80\%$.

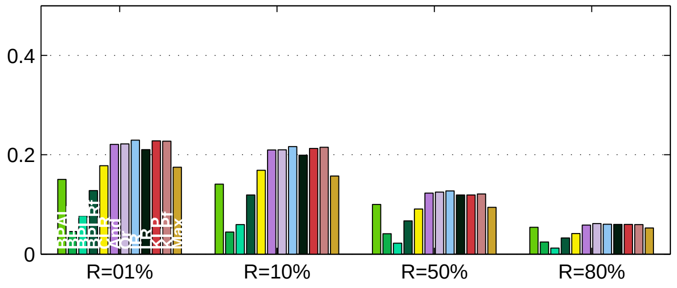

Figure S.2: **Proteins retrieved for a fixed GO term for the Biological Process ontology (test set)** In average over GO terms with the same recall levels: precisions obtained by different methods for values of the recall respectively fixed at $R = 1\%$, $R = 10\%$, $R = 50\%$, $R = 80\%$.

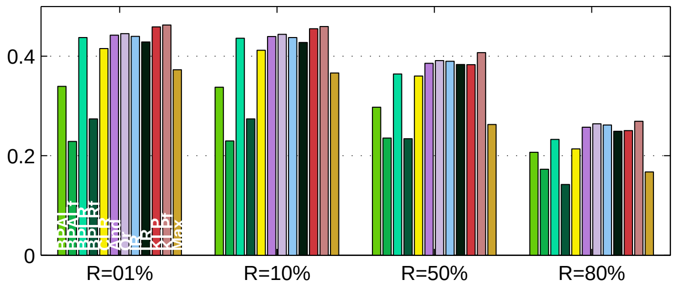

Figure S.3: **Proteins retrieved for a fixed GO term for the Molecular Function ontology (hold-out set)** In average over GO terms with the same recall levels: precisions obtained by different methods for values of the recall respectively fixed at $R = 1\%$, $R = 10\%$, $R = 50\%$, $R = 80\%$.

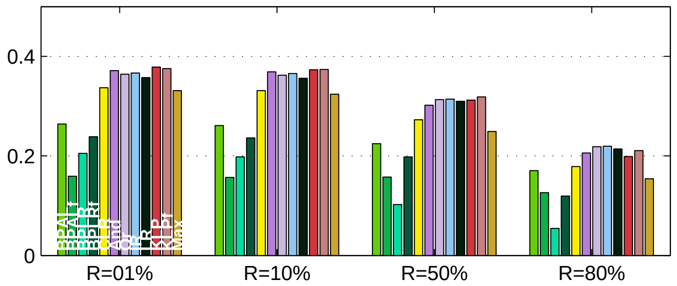

Figure S.4: **Proteins retrieved for a fixed GO term for the Molecular Function ontology (test set)** In average over GO terms with the same recall levels: precisions obtained by different methods for values of the recall respectively fixed at $R = 1\%$, $R = 10\%$, $R = 50\%$, $R = 80\%$.

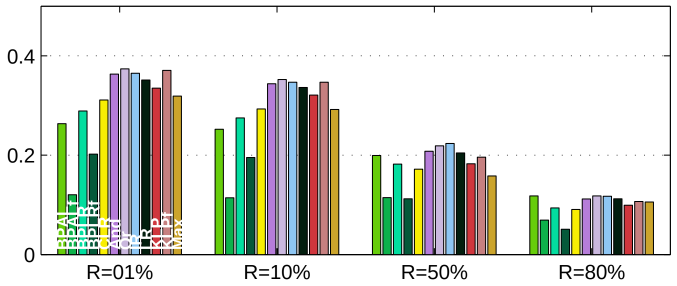

Figure S.5: **Proteins retrieved for a fixed GO term for the Cellular Component ontology (hold-out set)** In average over GO terms with the same recall levels: precisions obtained by different methods for values of the recall respectively fixed at $R = 1\%$, $R = 10\%$, $R = 50\%$, $R = 80\%$.

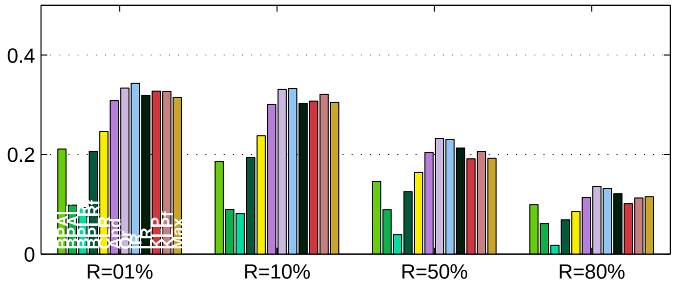

Figure S.6: **Proteins retrieved for a fixed GO term for the Cellular Component ontology (test set)** In average over GO terms with the same recall levels: precisions obtained by different methods for values of the recall respectively fixed at $R = 1\%$, $R = 10\%$, $R = 50\%$, $R = 80\%$.

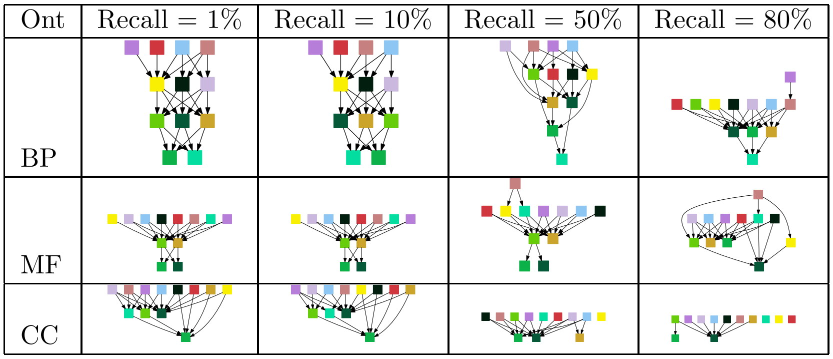

Figure S.7: **Statistical significance testing of per term evaluation, irrespective of term size.** Each panel shows a directed graph in which nodes are methods and a directed edge from node A to node B indicates that method A performs significantly better than method B according to the Z-test.

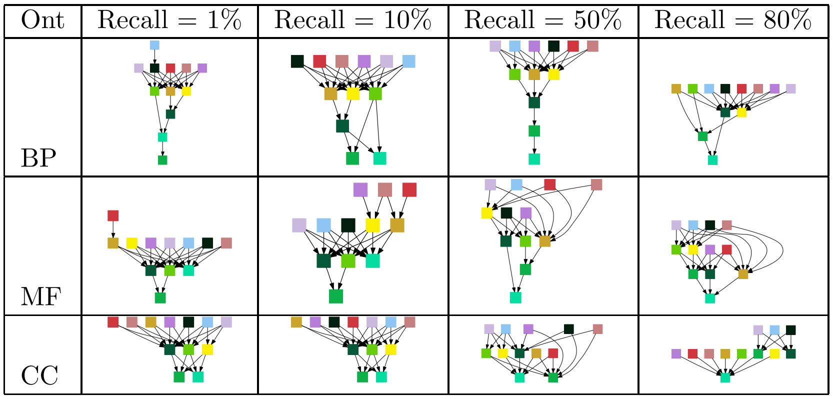

Figure S.8: **Statistical significance testing of per term evaluation, irrespective of term size.** Each panel shows a directed graph in which nodes are methods and a directed edge from node A to node B indicates that method A performs significantly better than method B according to the Z-test.

1.2 Directed graphs by ontology

1.3 Bar plots by ontology and term size

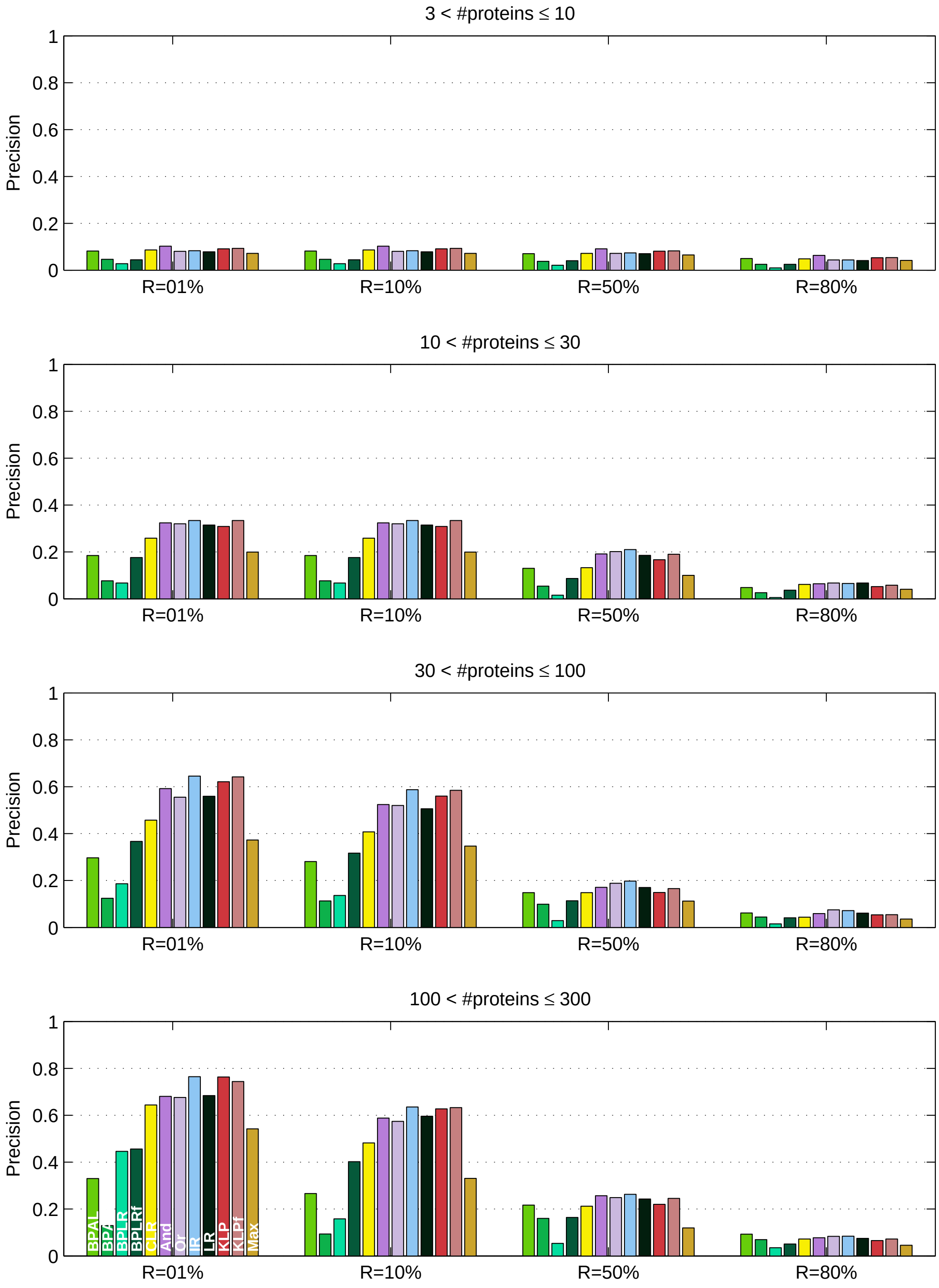

Figure S.9: **Proteins retrieved for a fixed GO term for the Biological Process ontology (hold-out set)** In average over GO terms with the same recall levels: precisions obtained by different methods for values of the recall respectively fixed at $R = 1\%$, $R = 10\%$, $R = 50\%$, $R = 80\%$. GO terms are grouped by size.

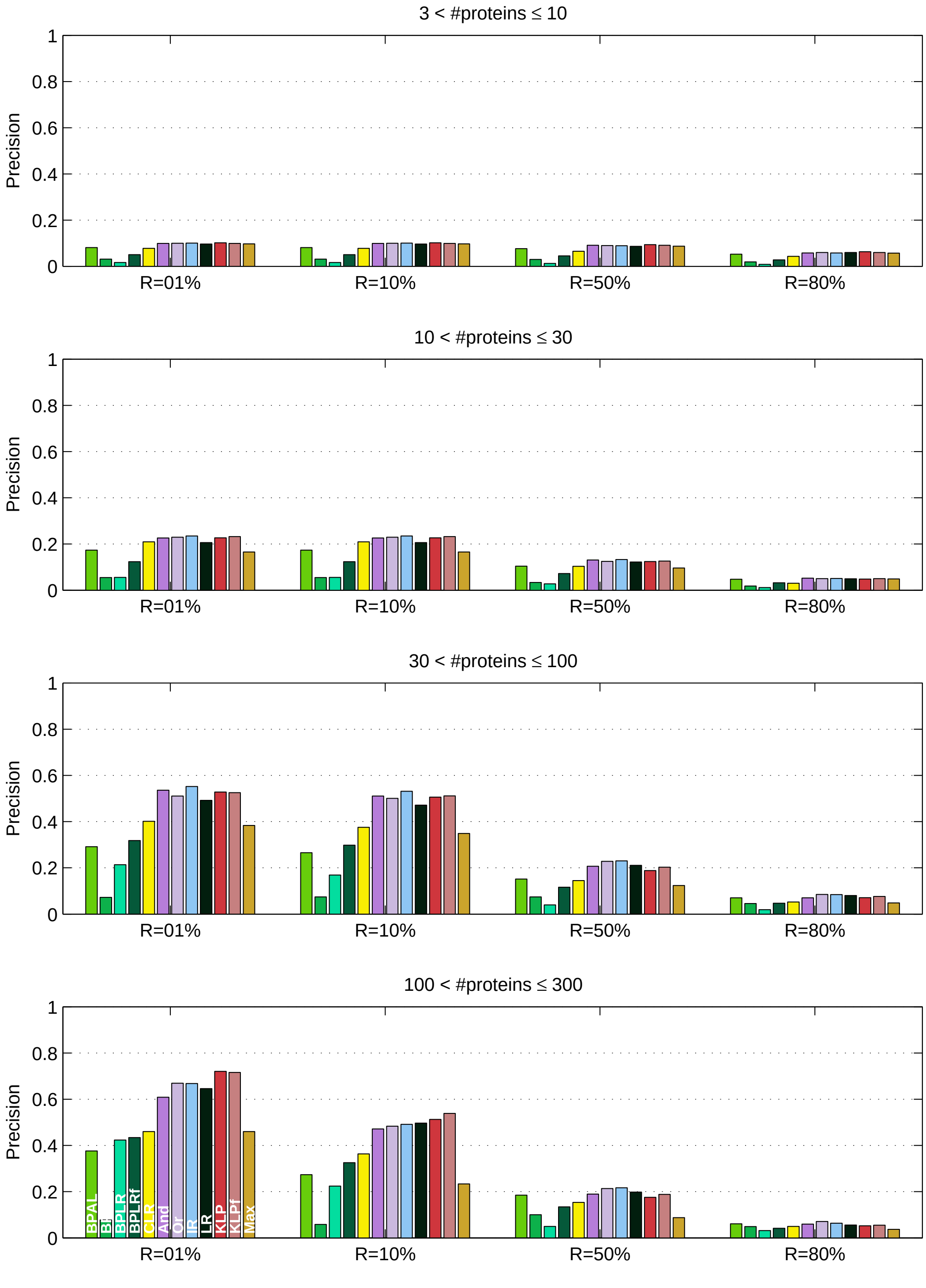

Figure S.10: **Proteins retrieved for a fixed GO term for the Biological Process ontology (test set)** In average over GO terms with the same recall levels: precisions obtained by different methods for values of the recall respectively fixed at $R = 1\%$, $R = 10\%$, $R = 50\%$, $R = 80\%$. GO terms are grouped by size.

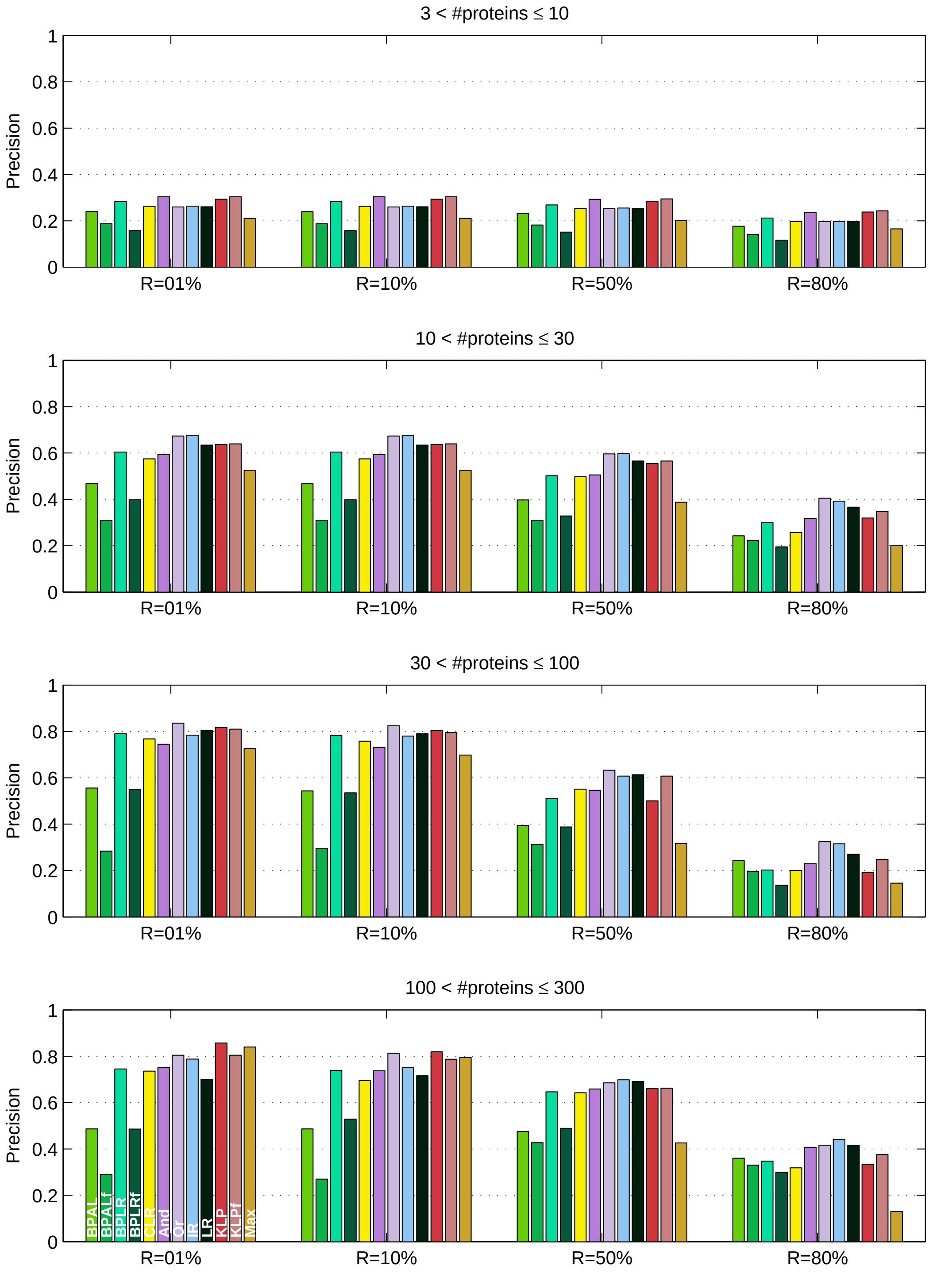

Figure S.11: **Proteins retrieved for a fixed GO term for the Molecular Function ontology (hold-out set)** In average over GO terms with the same recall levels: precisions obtained by different methods for values of the recall respectively fixed at $R = 1\%$, $R = 10\%$, $R = 50\%$, $R = 80\%$. GO terms are grouped by size.

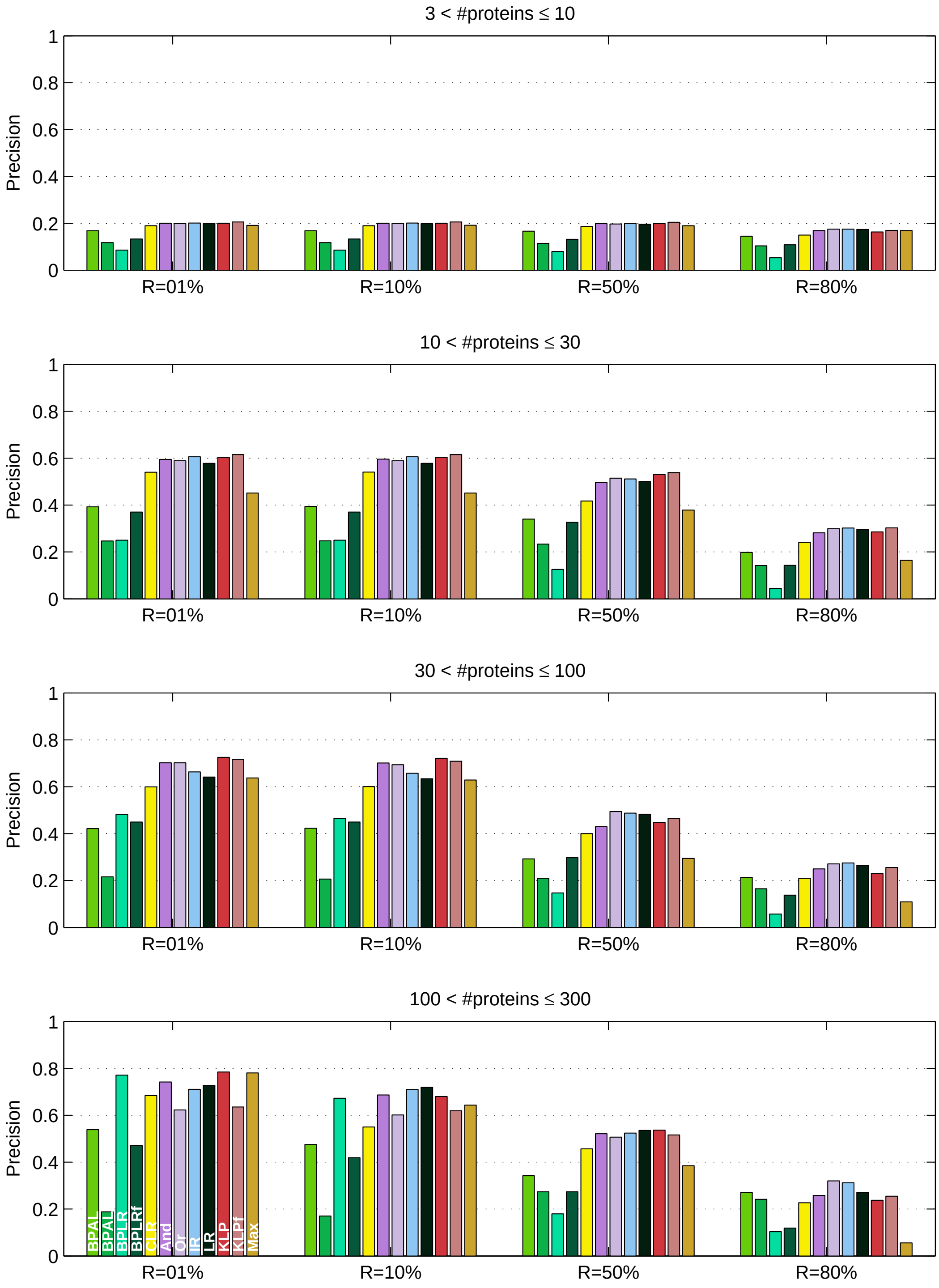

Figure S.12: **Proteins retrieved for a fixed GO term for the Molecular Function ontology (test set)** In average over GO terms with the same recall levels: precisions obtained by different methods for values of the recall respectively fixed at $R = 1\%$, $R = 10\%$, $R = 50\%$, $R = 80\%$. GO terms are grouped by size.

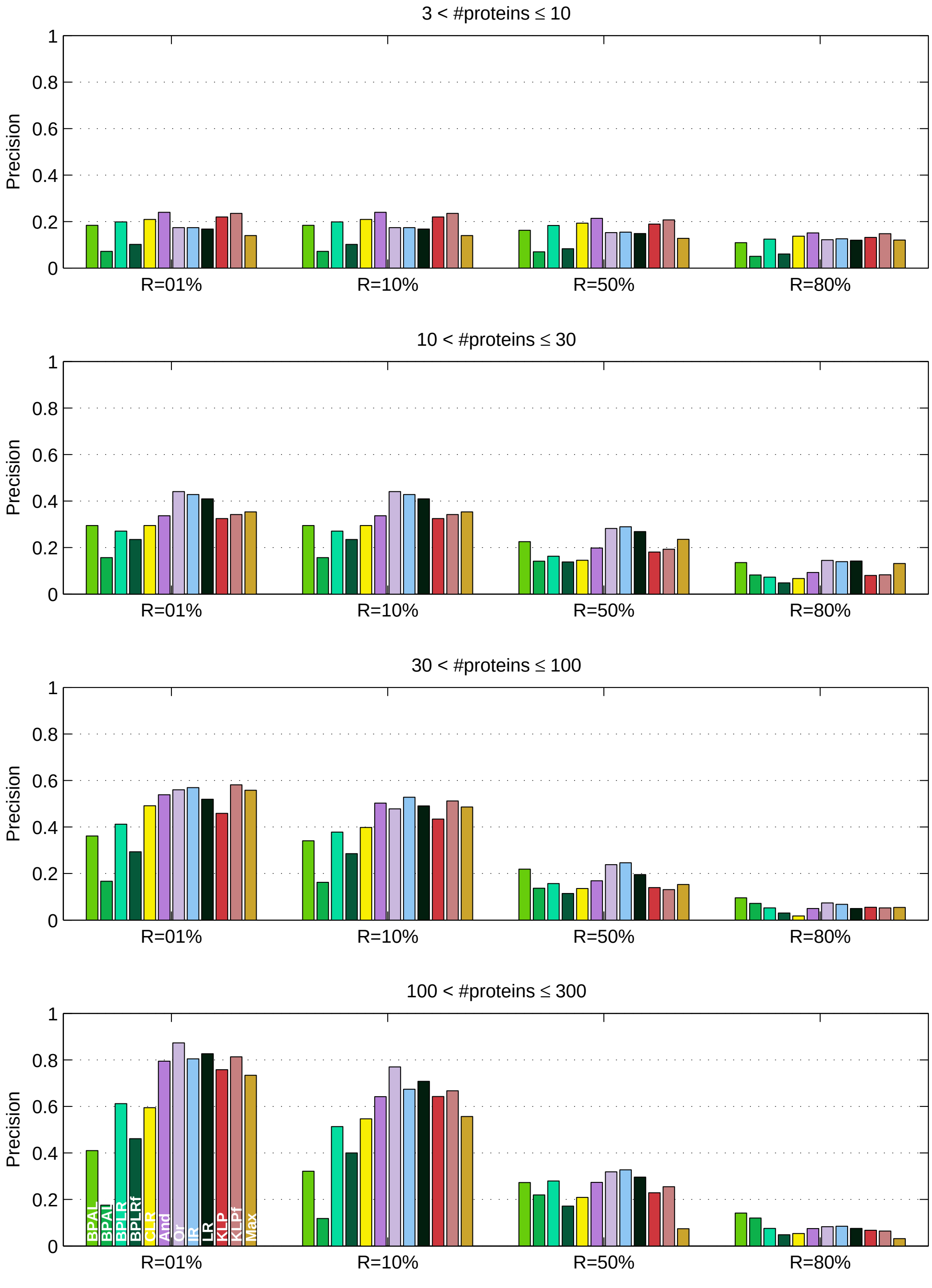

Figure S.13: **Proteins retrieved for a fixed GO term for the Cellular Component ontology (hold-out set)** In average over GO terms with the same recall levels: precisions obtained by different methods for values of the recall respectively fixed at $R = 1\%$, $R = 10\%$, $R = 50\%$, $R = 80\%$. GO terms are grouped by size.

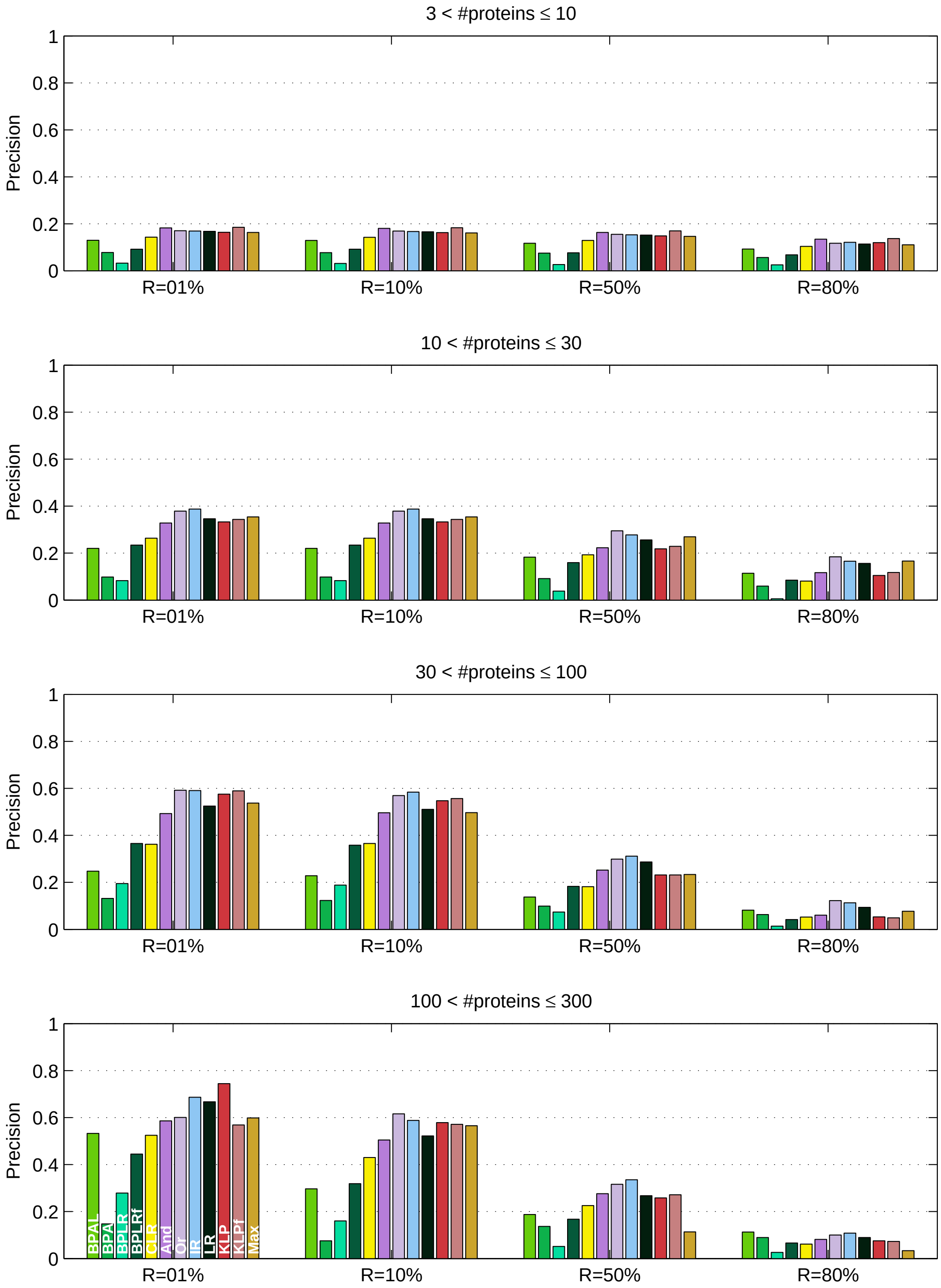

Figure S.14: **Proteins retrieved for a fixed GO term for the Cellular Component ontology (test set)** In average over GO terms with the same recall levels: precisions obtained by different methods for values of the recall respectively fixed at $R = 1\%$, $R = 10\%$, $R = 50\%$, $R = 80\%$. GO terms are grouped by size.

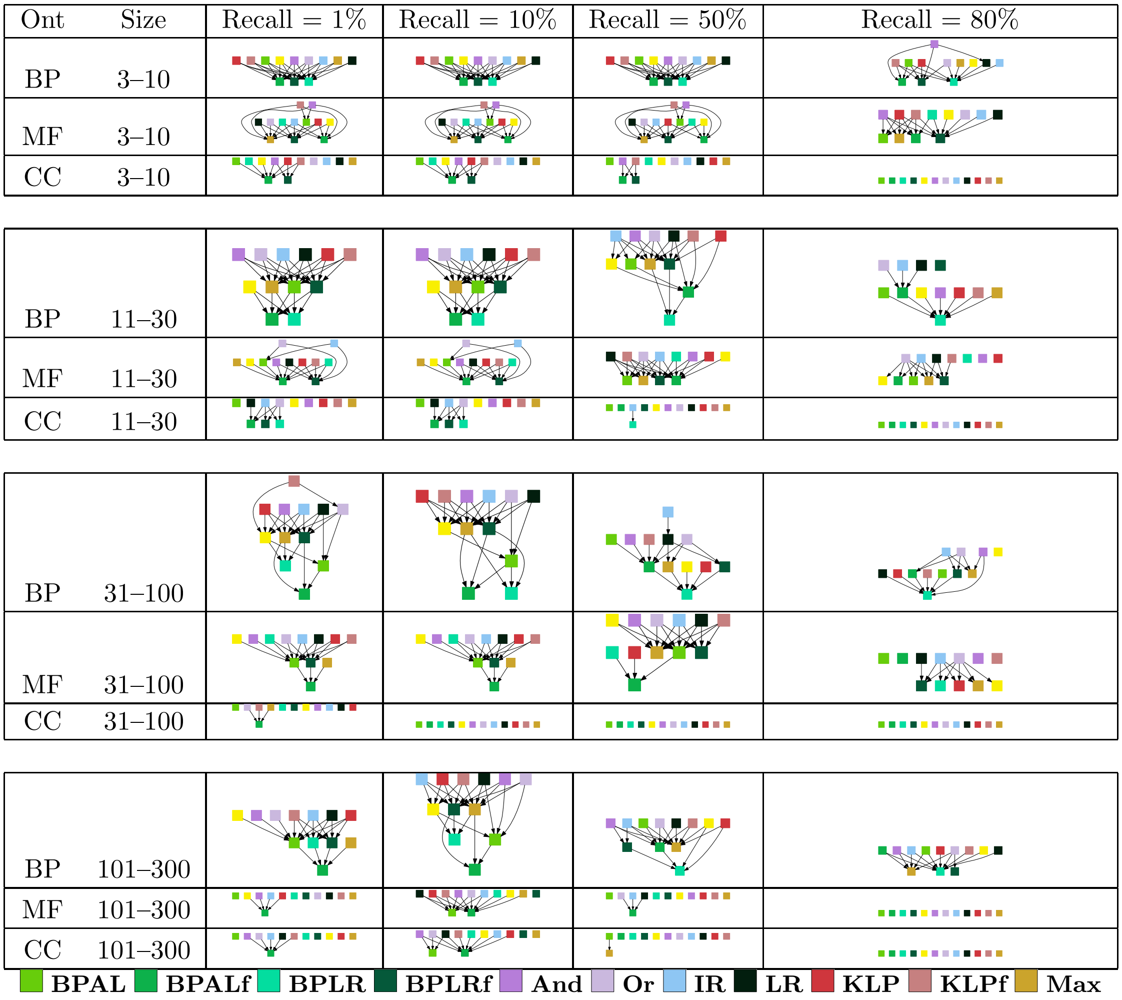

Figure S.15: **Statistical significance testing of per term evaluation** Each panel shows a directed graph in which nodes are methods and a directed edge from node A to node B indicates that method A performs significantly better than method B according to the Z-test.

1.4 Directed graphs by ontology and term size

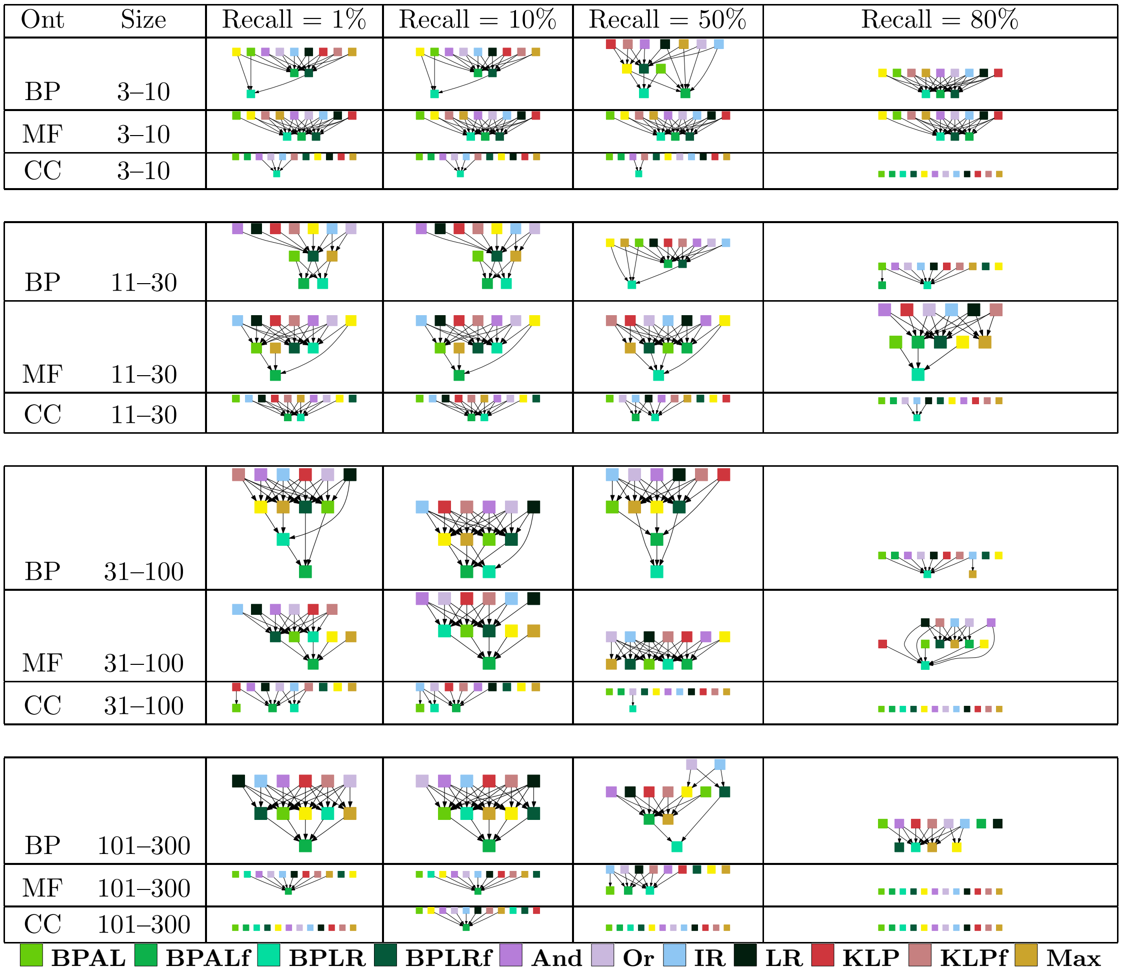

Figure S.16: **Statistical significance testing of per term evaluation** Each panel shows a directed graph in which nodes are methods and a directed edge from node A to node B indicates that method A performs significantly better than method B according to the Z-test.

2 Per protein evaluation

2.1 Bar plots by ontology

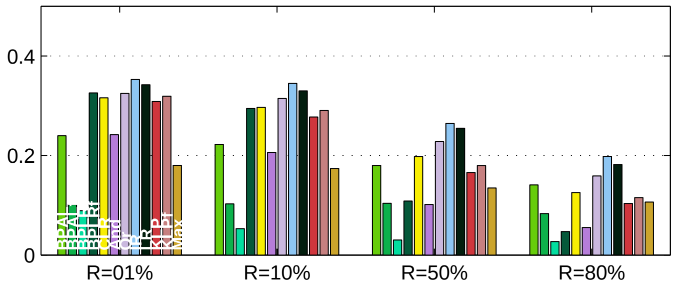

Figure S.17: **GO terms correctly found for a given protein for the Biological Process ontology (hold-out set)** Average over proteins of the precisions obtained by different methods for values of the recall respectively fixed at $R = 1\%$, $R = 10\%$, $R = 50\%$, $R = 80\%$.

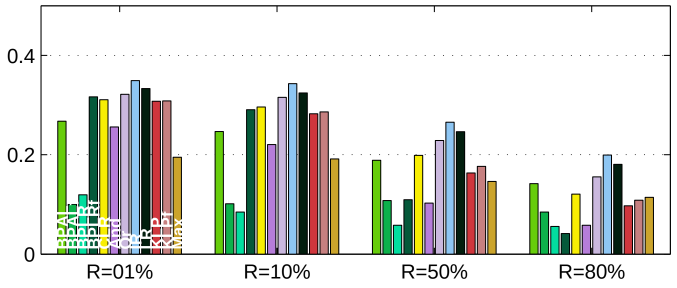

Figure S.18: **GO terms correctly found for a given protein for the Biological Process ontology (test set)** Average over proteins of the precisions obtained by different methods for values of the recall respectively fixed at $R = 1\%$, $R = 10\%$, $R = 50\%$, $R = 80\%$.

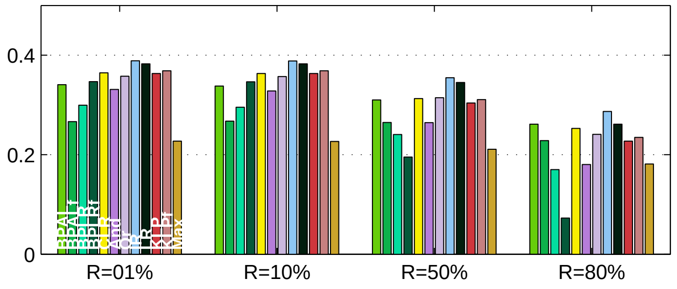

Figure S.19: **GO terms correctly found for a given protein for the Molecular Function ontology (hold-out set)** Average over proteins of the precisions obtained by different methods for values of the recall respectively fixed at $R = 1\%$, $R = 10\%$, $R = 50\%$, $R = 80\%$.

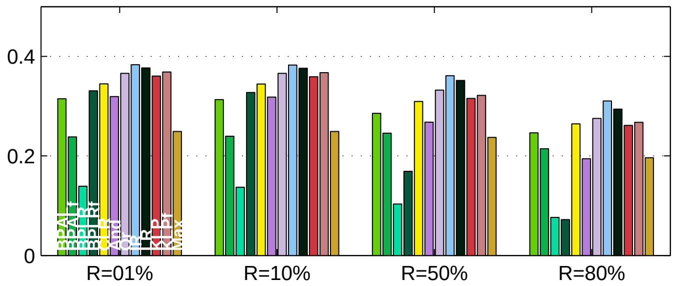

Figure S.20: **GO terms correctly found for a given protein for the Molecular Function ontology (test set)** Average over proteins of the precisions obtained by different methods for values of the recall respectively fixed at $R = 1\%$, $R = 10\%$, $R = 50\%$, $R = 80\%$.

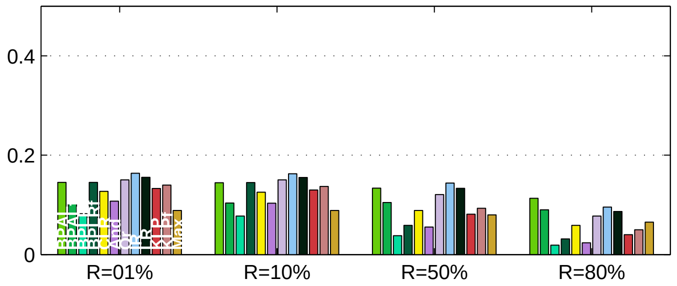

Figure S.21: **GO terms correctly found for a given protein for the Cellular Component ontology (hold-out set)** Average over proteins of the precisions obtained by different methods for values of the recall respectively fixed at $R = 1\%$, $R = 10\%$, $R = 50\%$, $R = 80\%$.

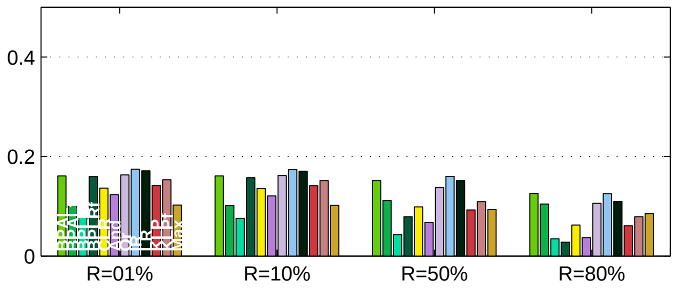

Figure S.22: **GO terms correctly found for a given protein for the Cellular Component ontology (test set)** Average over proteins of the precisions obtained by different methods for values of the recall respectively fixed at $R = 1\%$, $R = 10\%$, $R = 50\%$, $R = 80\%$.

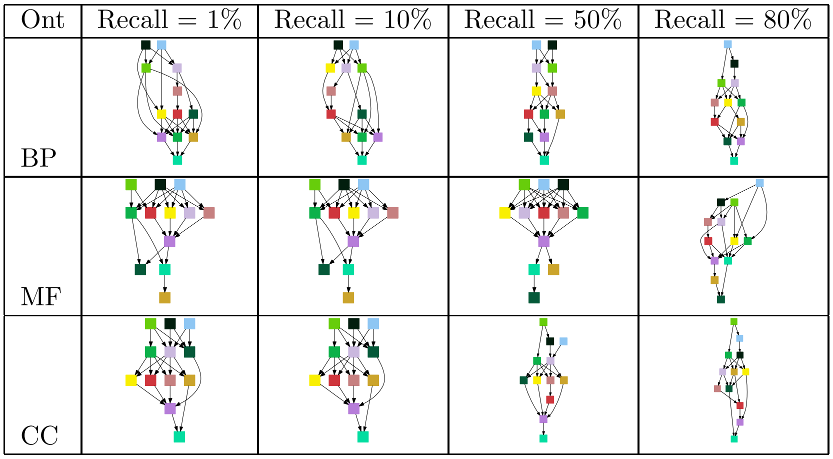

Figure S.23: **Statistical significance testing of per protein evaluation, irrespective of term size (hold-out set).** Each panel shows a directed graph in which nodes are methods and a directed edge from node A to node B indicates that method A performs significantly better than method B according to the Z-test.

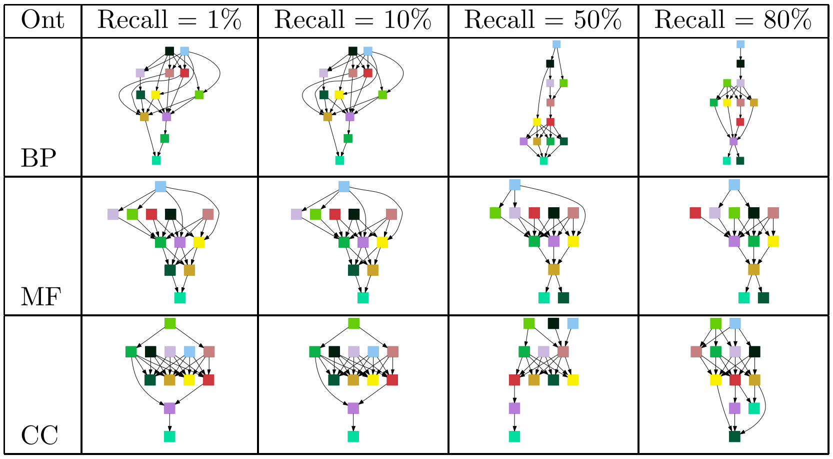

Figure S.24: **Statistical significance testing of per protein evaluation, irrespective of term size (test set).** Each panel shows a directed graph in which nodes are methods and a directed edge from node A to node B indicates that method A performs significantly better than method B according to the Z-test.

2.2 Directed graphs by ontology

2.3 Bar plots by ontology and term size

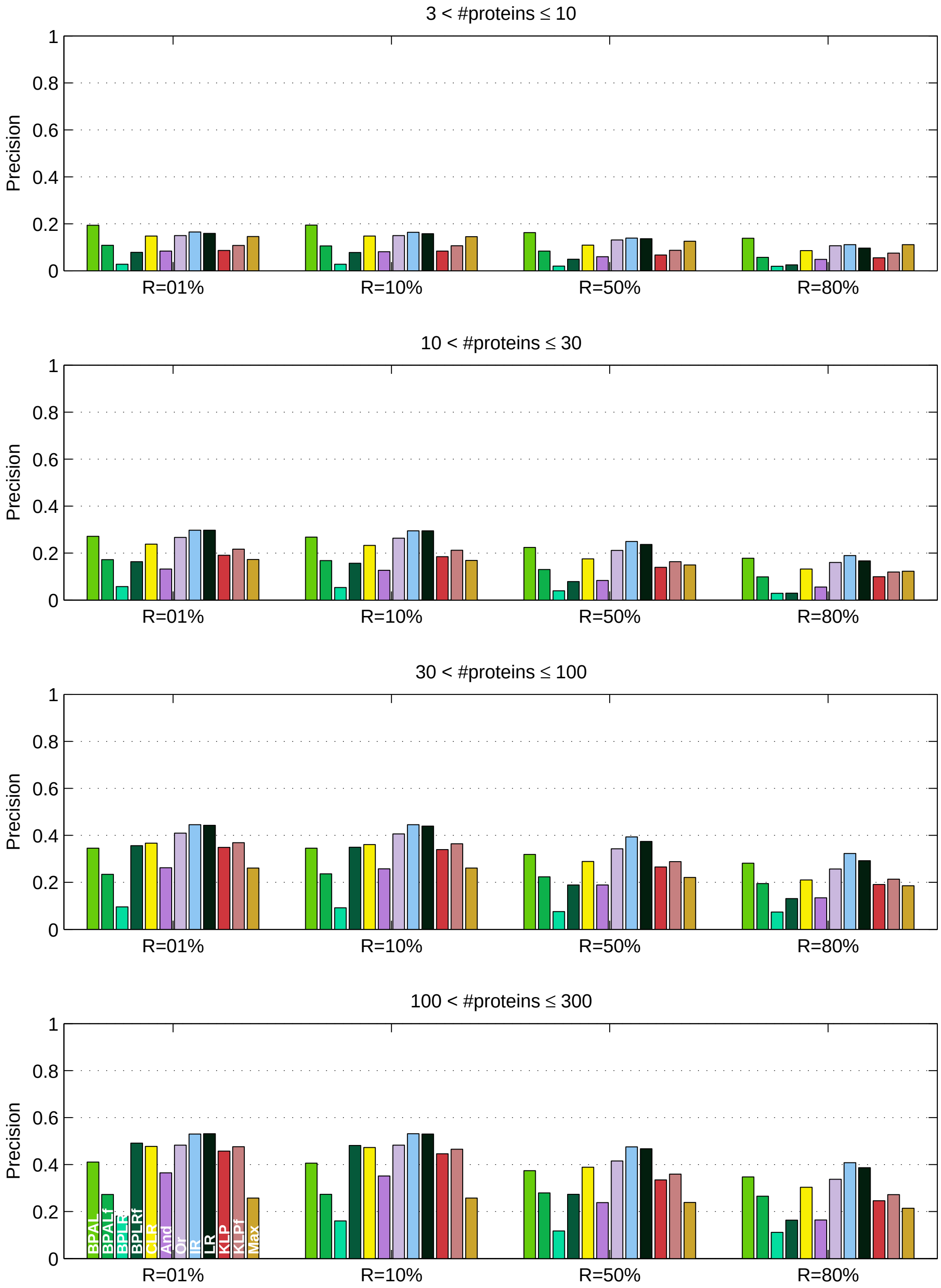

Figure S.25: **GO terms correctly found for a given protein for the Biological Process ontology (hold-out set)** Average over proteins of the precisions obtained by different methods for values of the recall respectively fixed at $R = 1\%$, $R = 10\%$, $R = 50\%$, $R = 80\%$. Proteins are grouped according to how many terms are known for these proteins.

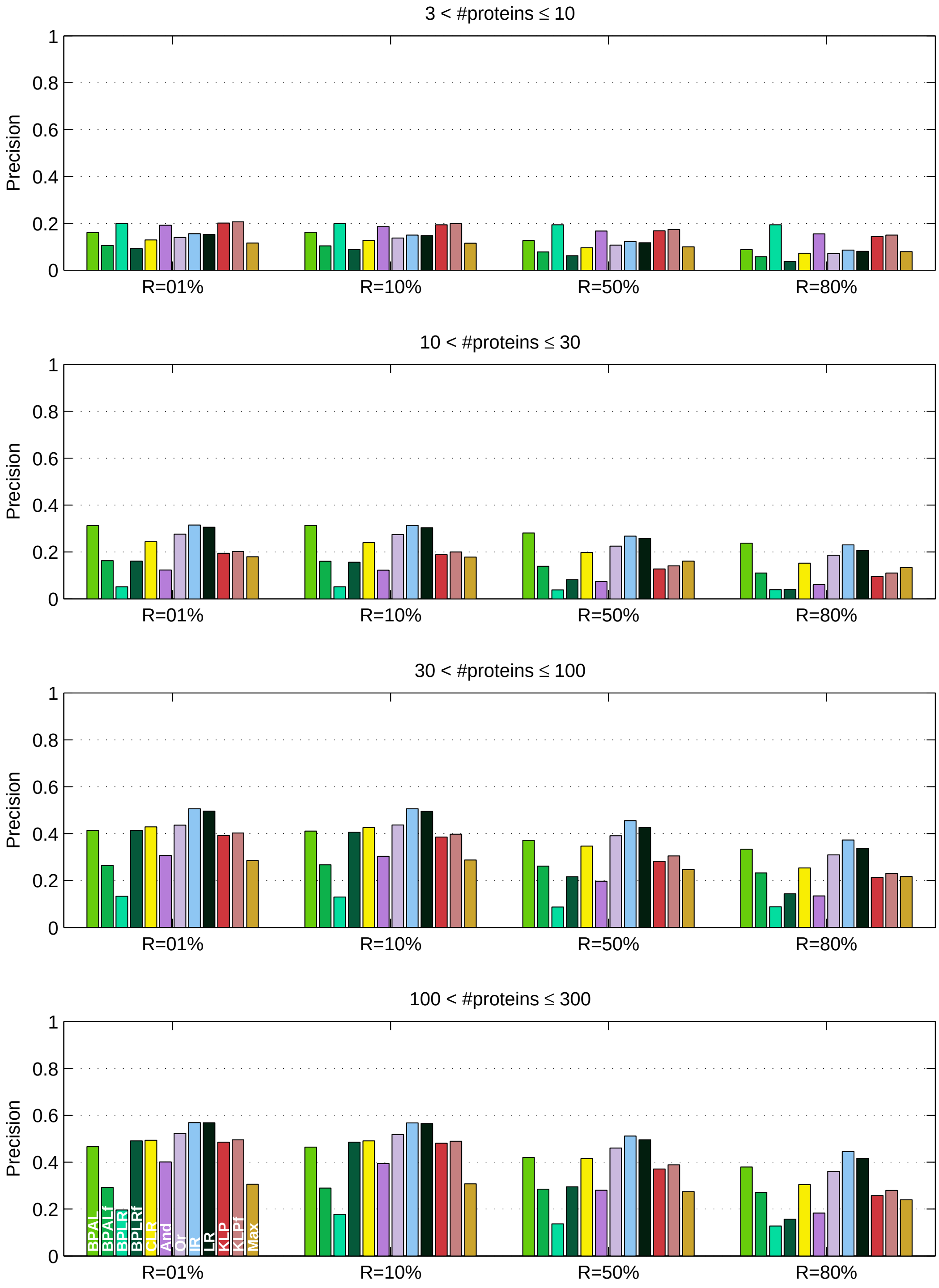

Figure S.26: **GO terms correctly found for a given protein for the Biological Process ontology (test set)** Average over proteins of the precisions obtained by different methods for values of the recall respectively fixed at $R = 1\%$, $R = 10\%$, $R = 50\%$, $R = 80\%$. Proteins are grouped according to how many terms are known for these proteins.

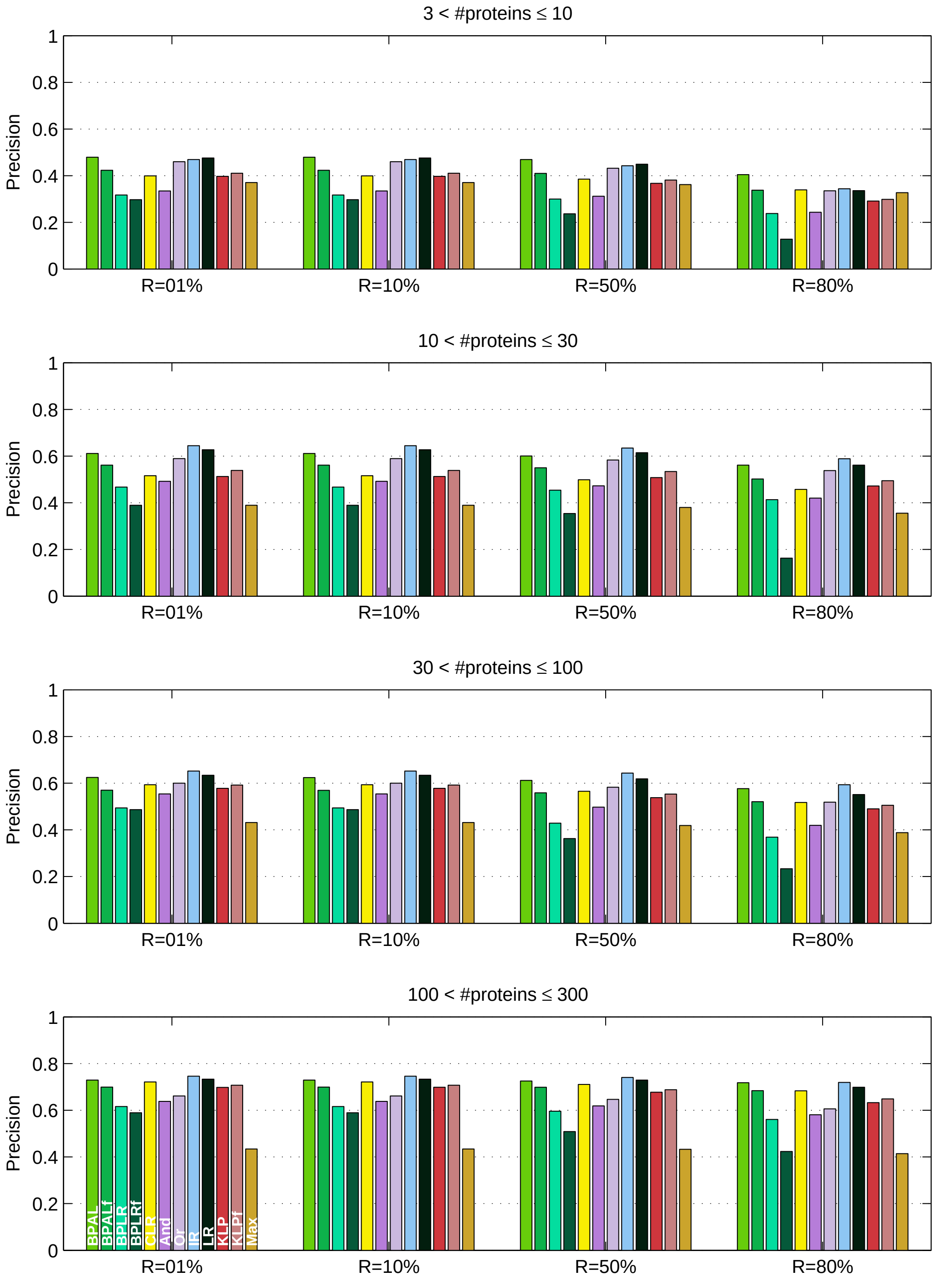

Figure S.27: **GO terms correctly found for a given protein for the Molecular Function ontology (hold-out set)** Average over proteins of the precisions obtained by different methods for values of the recall respectively fixed at $R = 1\%$, $R = 10\%$, $R = 50\%$, $R = 80\%$. Proteins are grouped according to how many terms are known for these proteins.

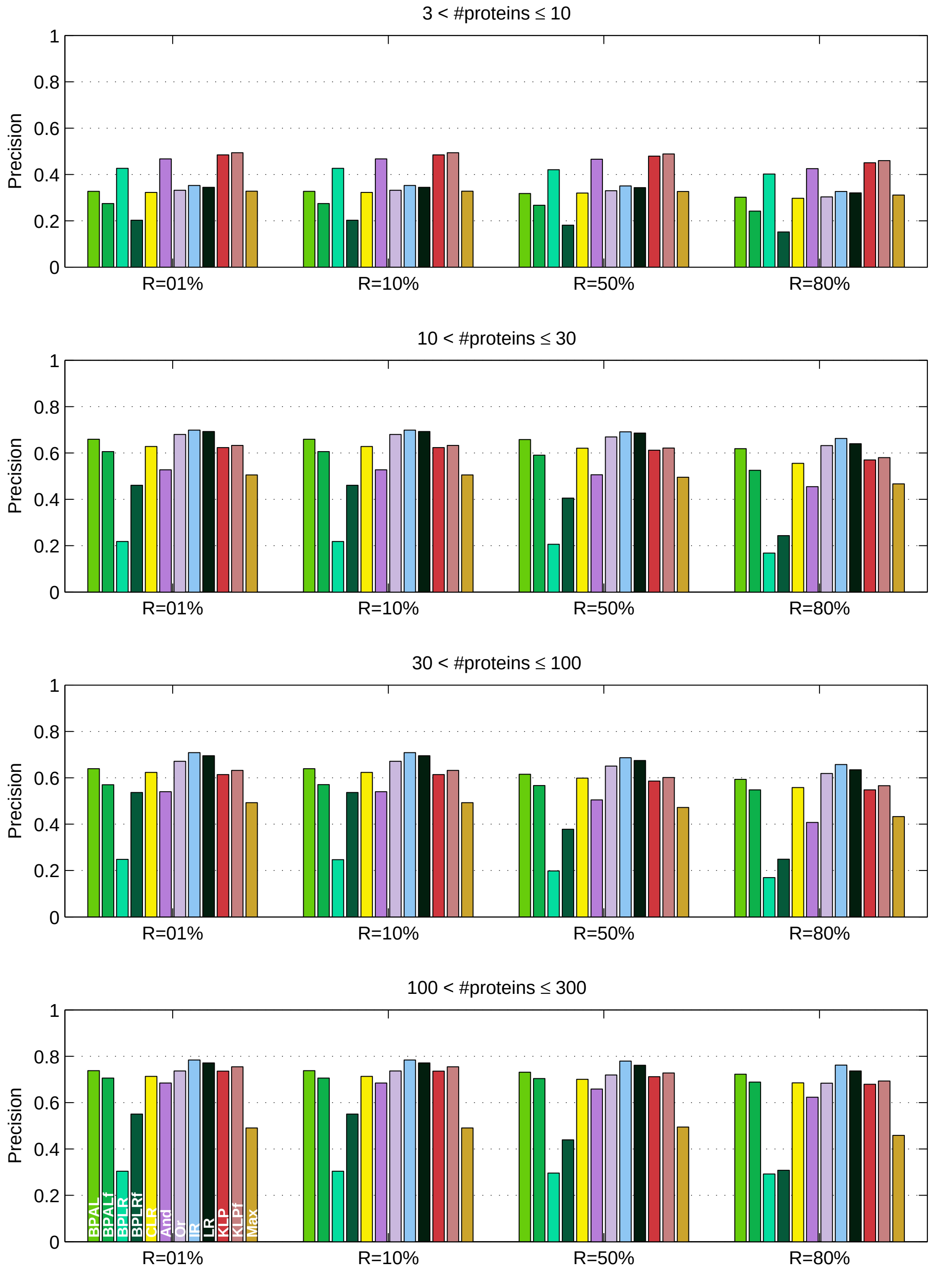

Figure S.28: **GO terms correctly found for a given protein for the Molecular Function ontology (test set)** Average over proteins of the precisions obtained by different methods for values of the recall respectively fixed at $R = 1\%$, $R = 10\%$, $R = 50\%$, $R = 80\%$. Proteins are grouped according to how many terms are known for these proteins.

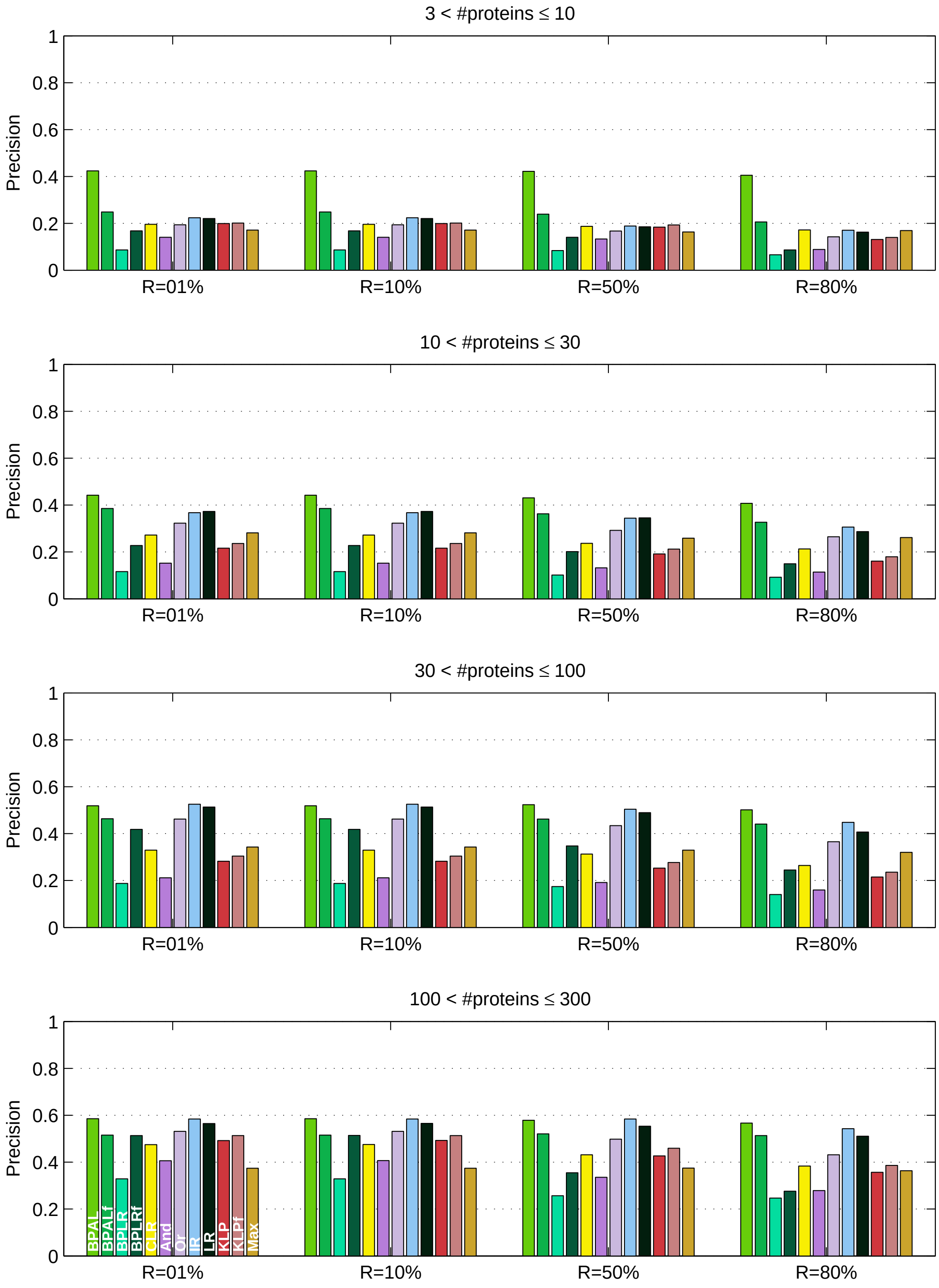

Figure S.29: **GO terms correctly found for a given protein for the Cellular Component ontology (hold-out set)** Average over proteins of the precisions obtained by different methods for values of the recall respectively fixed at $R = 1\%$, $R = 10\%$, $R = 50\%$, $R = 80\%$. Proteins are grouped according to how many terms are known for these proteins.

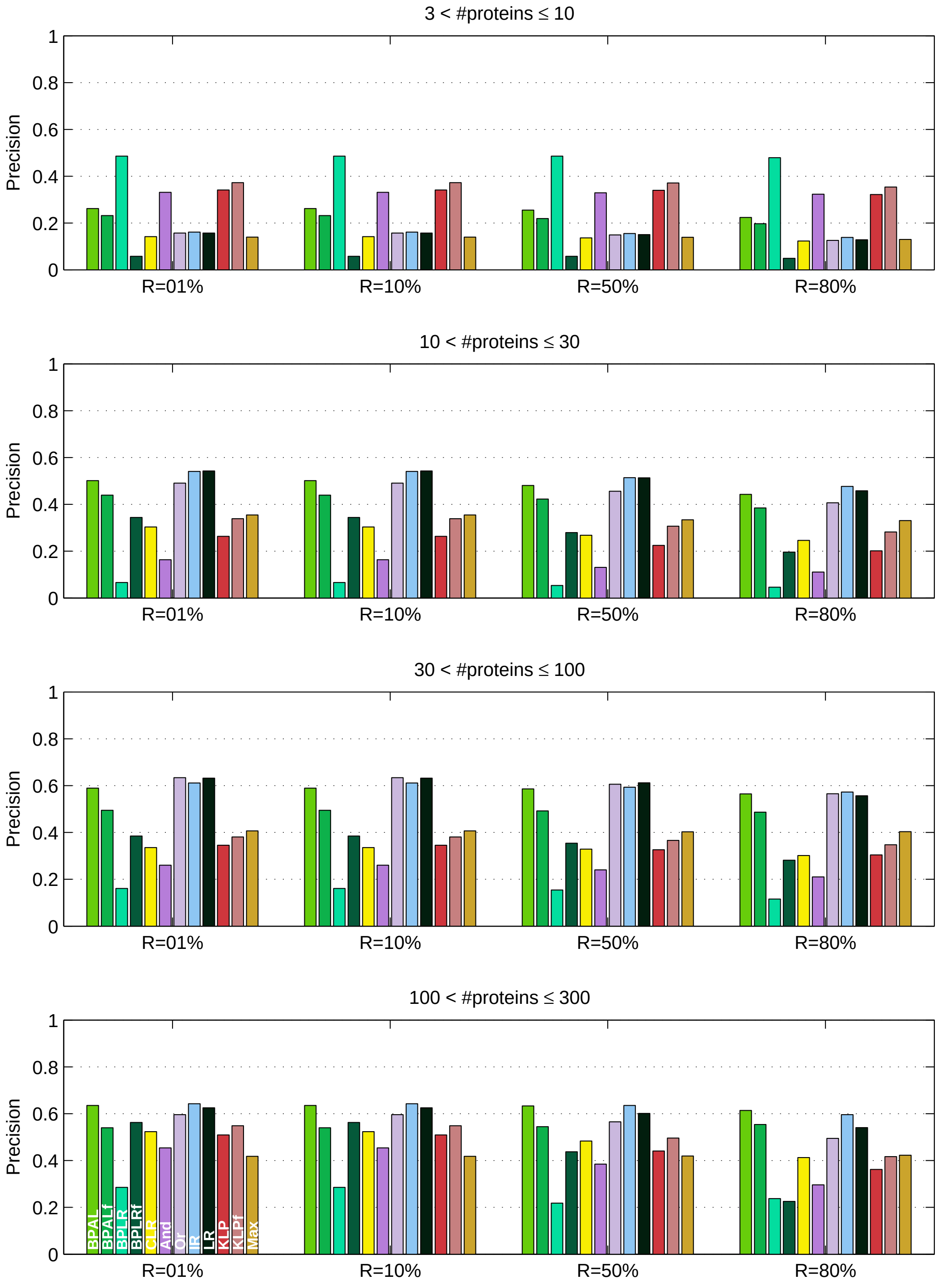

Figure S.30: **GO terms correctly found for a given protein for the Cellular Component ontology (test set)** Average over proteins of the precisions obtained by different methods for values of the recall respectively fixed at $R = 1\%$, $R = 10\%$, $R = 50\%$, $R = 80\%$. Proteins are grouped according to how many terms are known for these proteins.

2.4 Directed graphs by ontology and term size

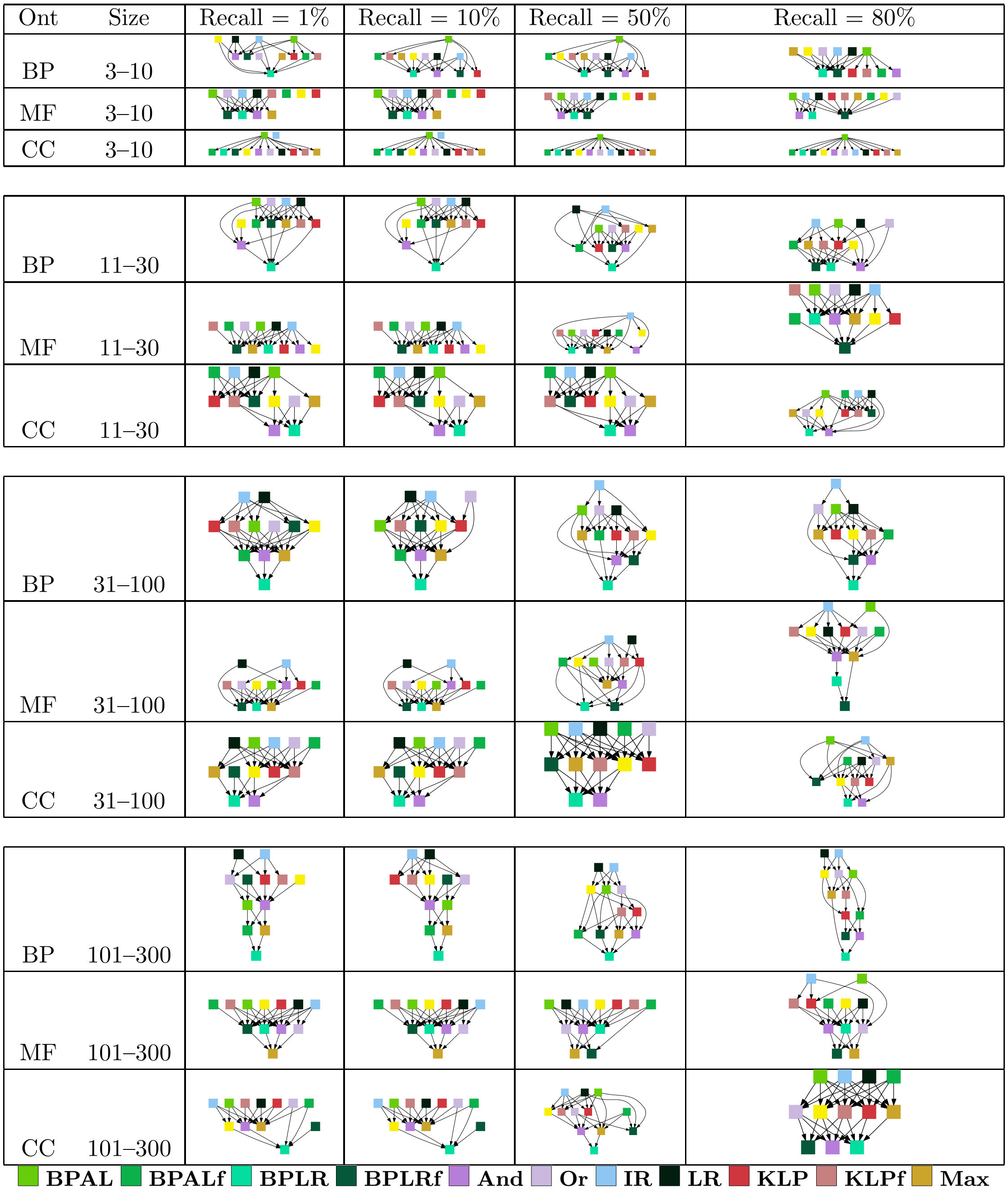

Figure S.31: **Statistical significance testing of per protein evaluation (hold-out set)** Each panel shows a directed graph in which nodes are methods and a directed edge from node A to node B indicates that method A performs significantly better than method B according to the Z-test.

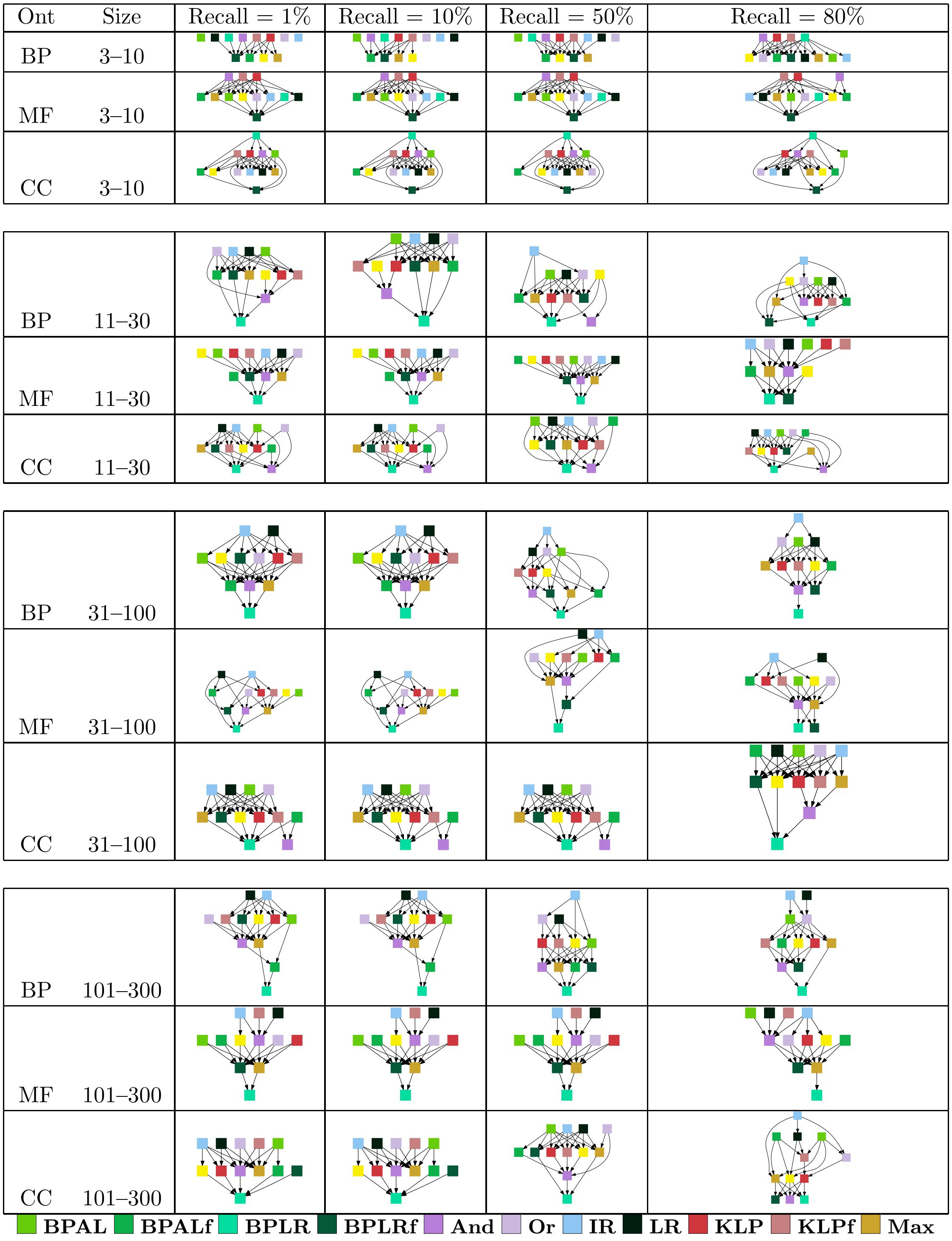

Figure S.32: **Statistical significance testing of per protein evaluation (test set)** Each panel shows a directed graph in which nodes are methods and a directed edge from node A to node B indicates that method A performs significantly better than method B according to the Z-test.

3 Joint annotation evaluation

3.1 Precision-recall curves by ontology

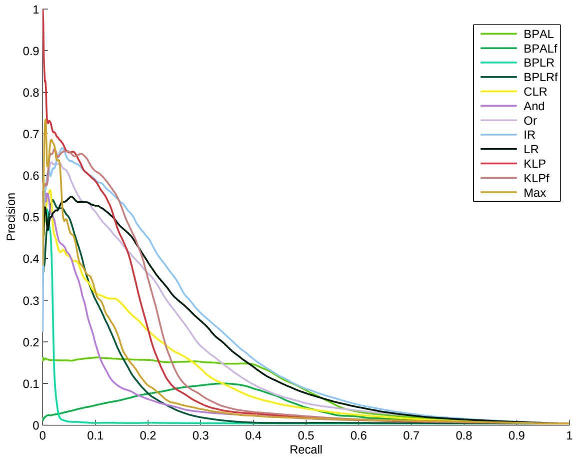

Figure S.33: **Precision-recall curve for joint annotation in the Biological Process ontology (hold-out set)** Precision-recall curve for the retrieval of valid (protein,term) pairs.

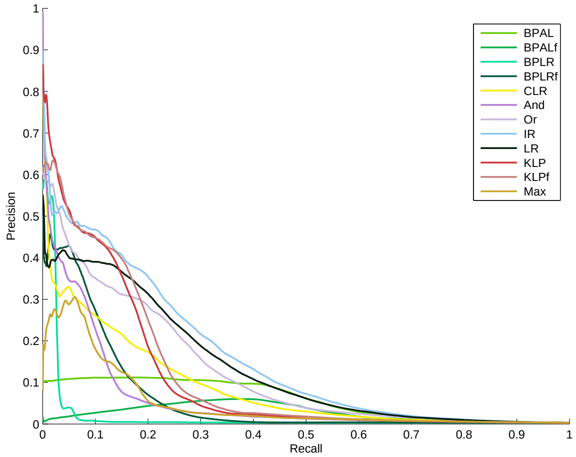

Figure S.34: **Precision-recall curve for joint annotation in the Biological Process ontology (test set)** Precision-recall curve for the retrieval of valid (protein,term) pairs.

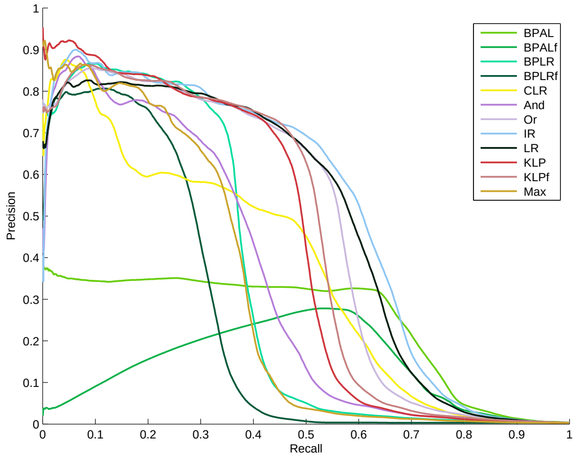

Figure S.35: **Precision-recall curve for joint annotation in the Molecular Function ontology (hold-out set)** Precision-recall curve for the retrieval of valid (protein,term) pairs.

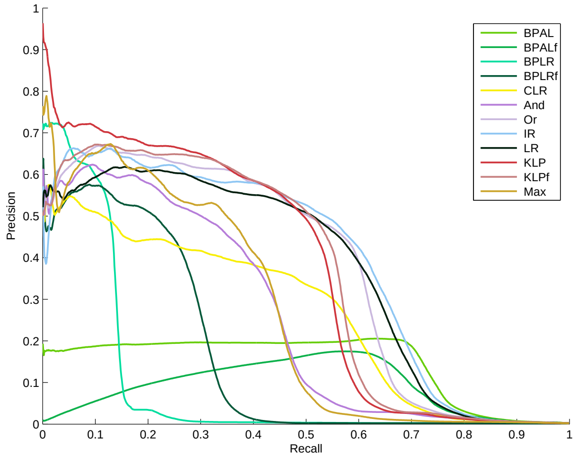

Figure S.36: **Precision-recall curve for joint annotation in the Molecular Function ontology (test set)** Precision-recall curve for the retrieval of valid (protein,term) pairs.

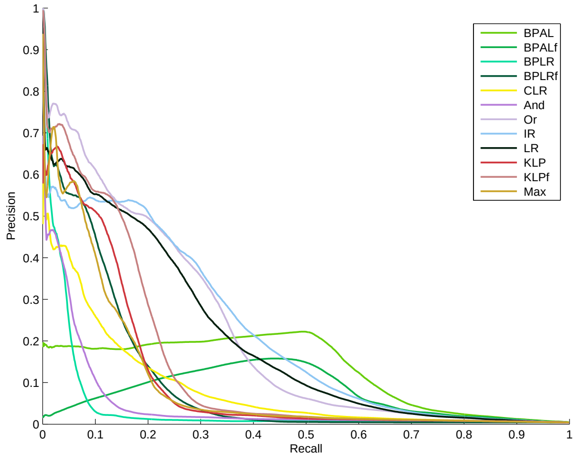

Figure S.37: **Precision-recall curve for joint annotation in the Cellular Component ontology (hold-out set)** Precision-recall curve for the retrieval of valid (protein,term) pairs.

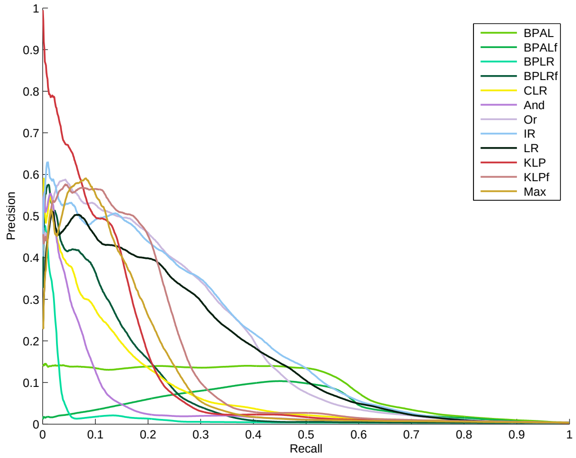

Figure S.38: **Precision-recall curve for joint annotation in the Cellular Component ontology (test set)** Precision-recall curve for the retrieval of valid (protein,term) pairs.

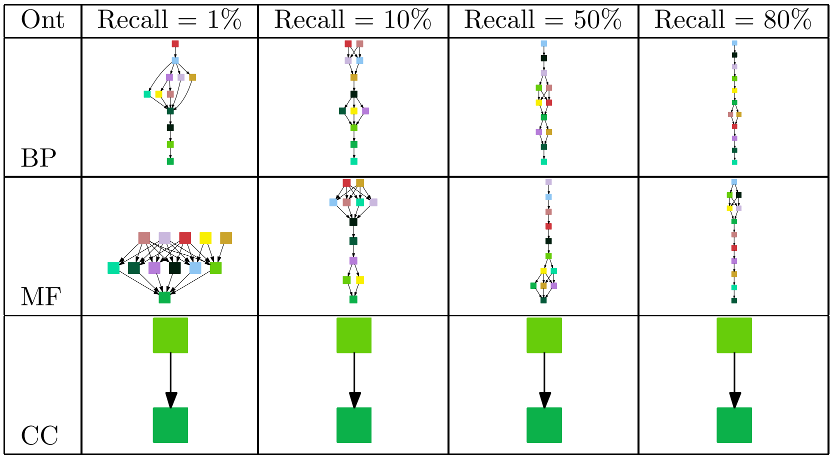

Figure S.39: **Statistical significance testing of joint evaluation, irrespective of term size (hold-out set).** Each panel shows a directed graph in which nodes are methods and a directed edge from node A to node B indicates that method A performs significantly better than method B according to the bootstrap test.

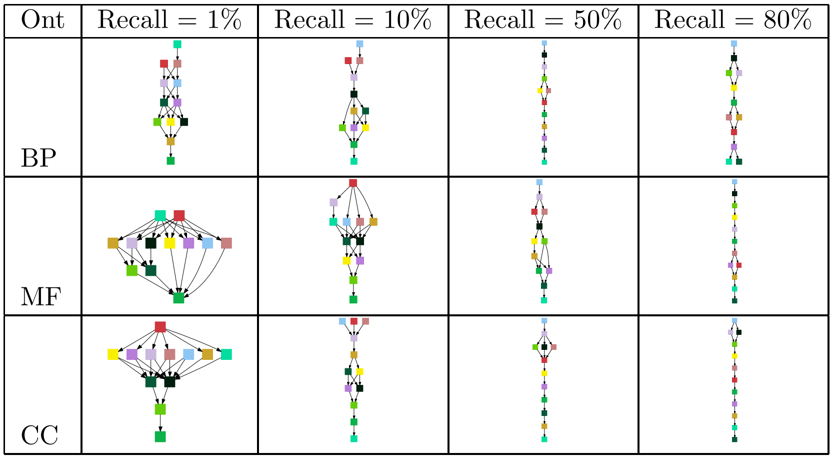

Figure S.40: **Statistical significance testing of joint evaluation, irrespective of term size (test set).** Each panel shows a directed graph in which nodes are methods and a directed edge from node A to node B indicates that method A performs significantly better than method B according to the bootstrap.

3.2 Directed graphs by ontology

3.3 Precision-recall curves by ontology and term size

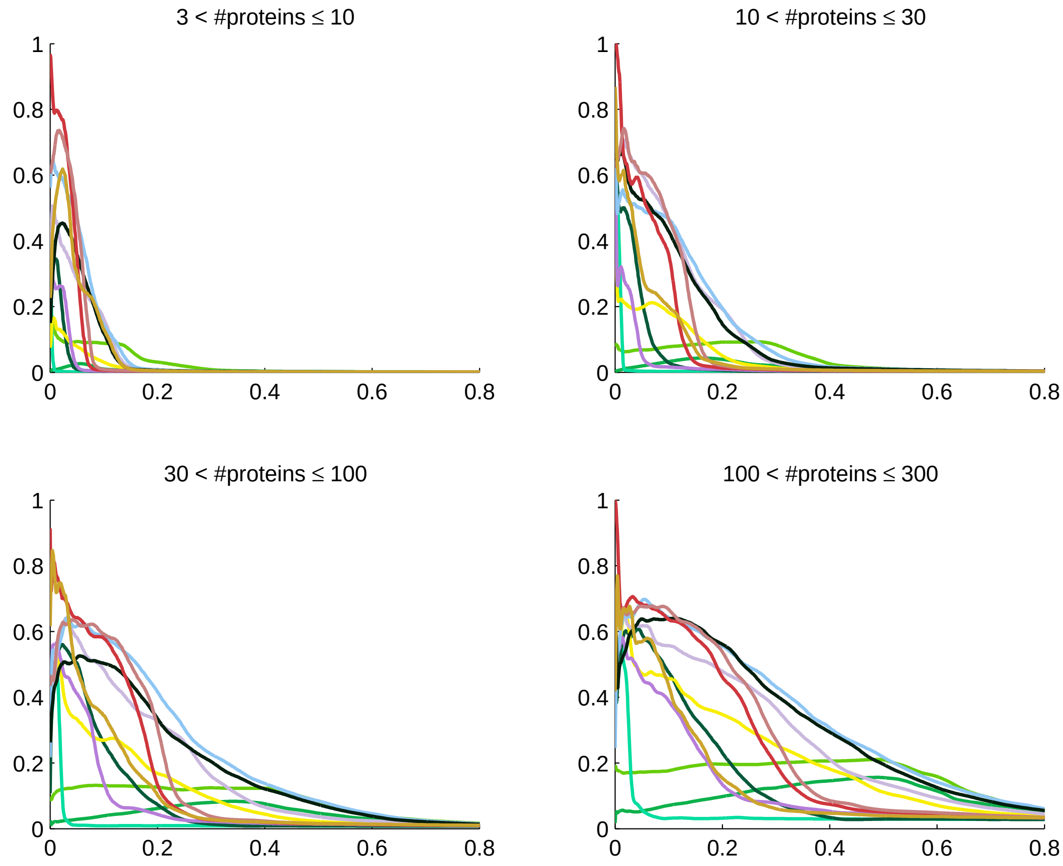

Figure S.41: **Precision-recall curves per size for joint annotation in the Biological Process ontology (hold-out set)** Precision-recall curve for 4 groups of terms with different levels of specificity in the ontology.

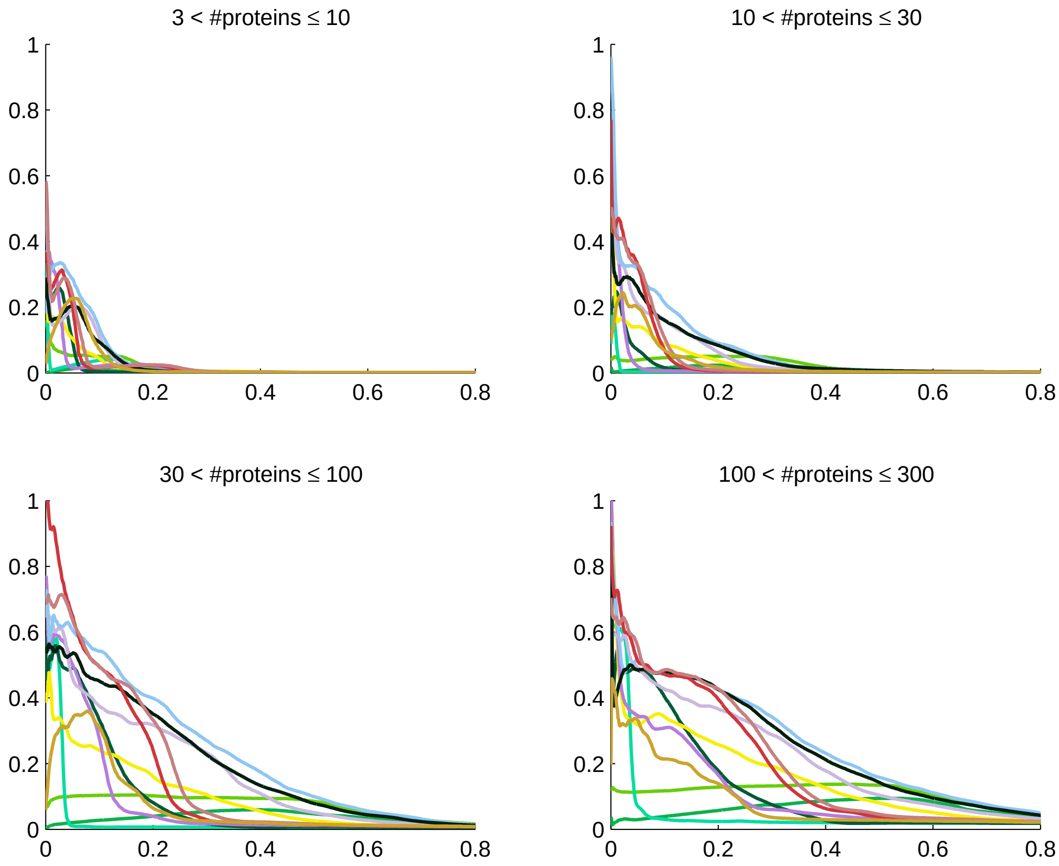

Figure S.42: **Precision-recall curves per size for joint annotation in the Biological Process ontology (test set)** Precision-recall curve for 4 groups of terms with different levels of specificity in the ontology.

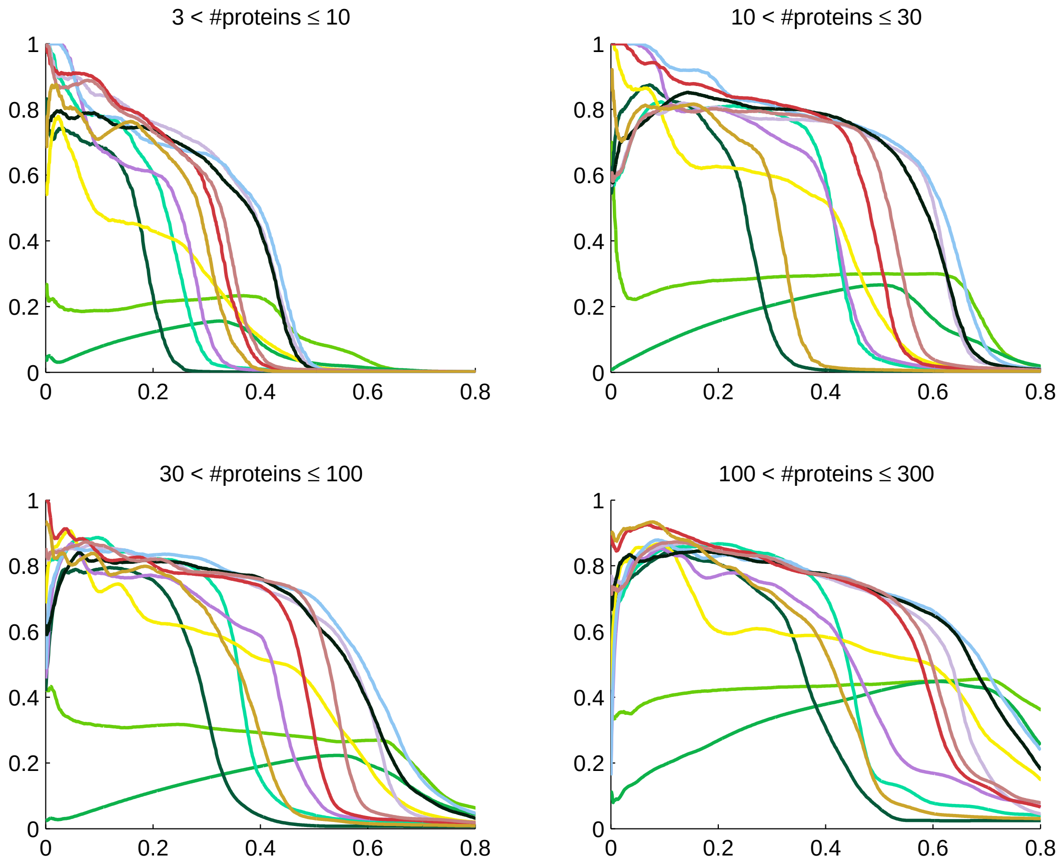

Figure S.43: **Precision-recall curves per size for joint annotation in the Molecular Function ontology (hold-out set)** Precision-recall curve for 4 groups of terms with different levels of specificity in the ontology.

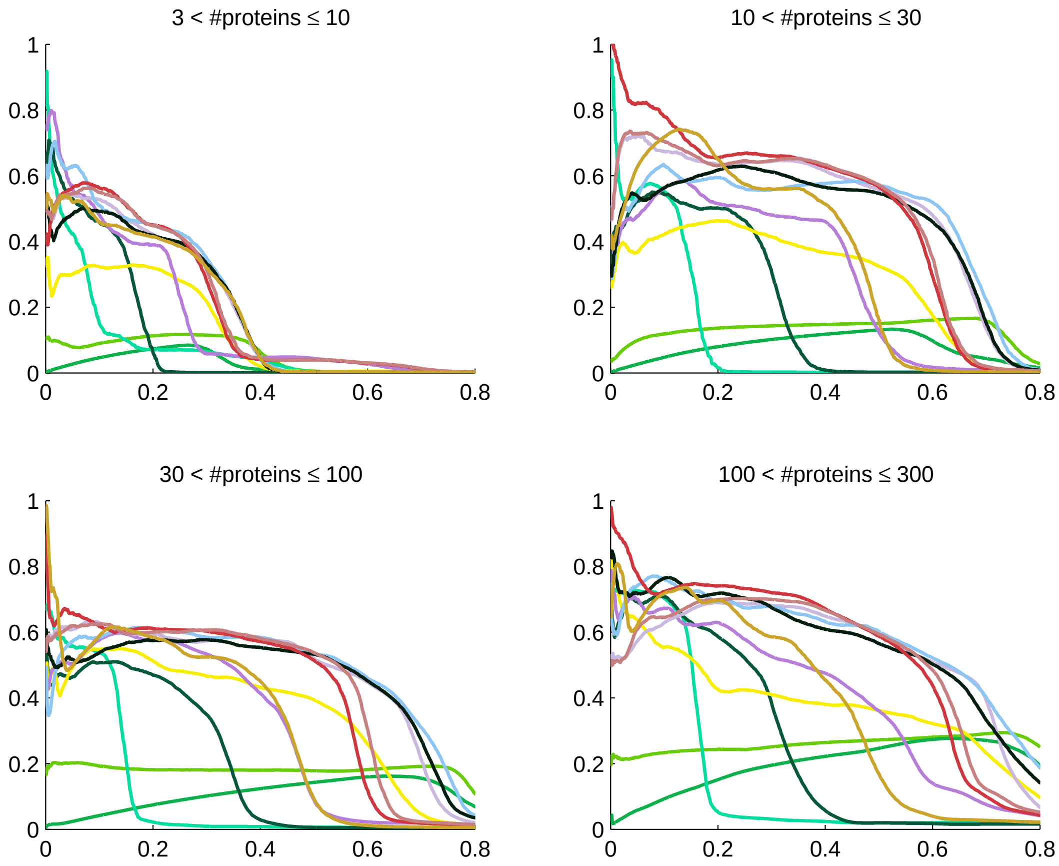

Figure S.44: **Precision-recall curves per size for joint annotation in the Molecular Function ontology (test set)** Precision-recall curve for 4 groups of terms with different levels of specificity in the ontology.

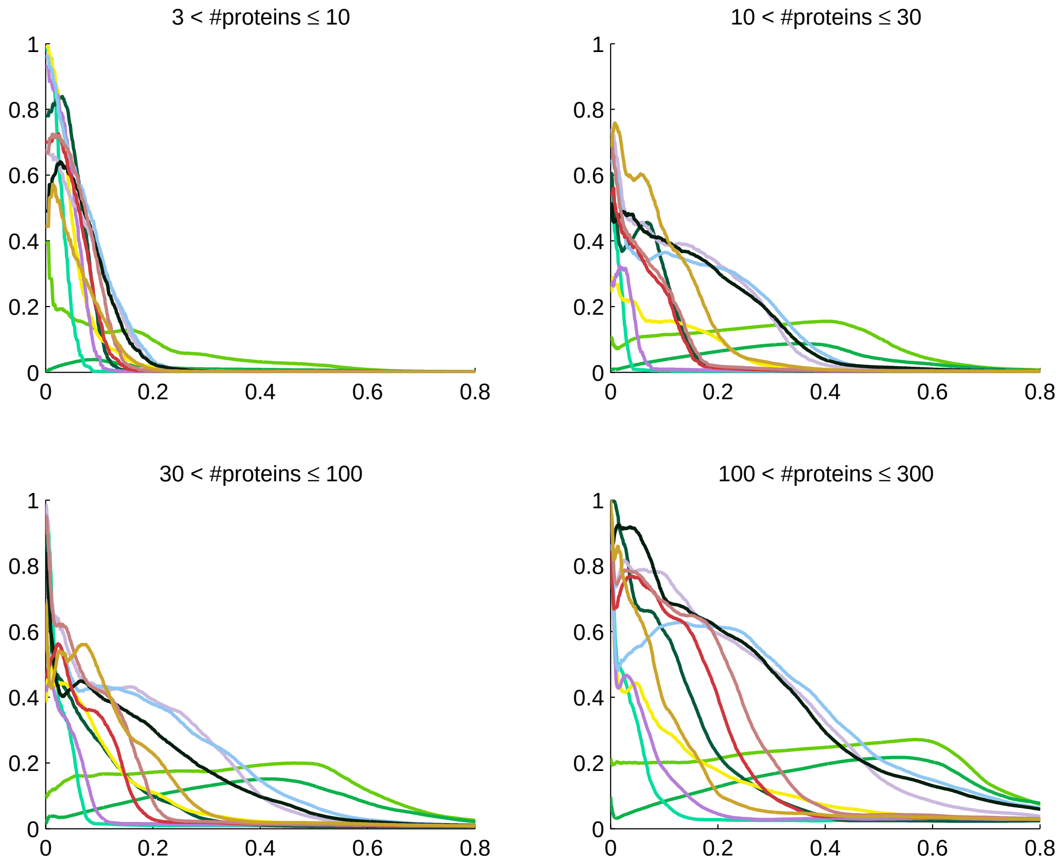

Figure S.45: **Precision-recall curves per size for joint annotation in the Cellular Component ontology (hold-out set)** Precision-recall curve for 4 groups of terms with different levels of specificity in the ontology.

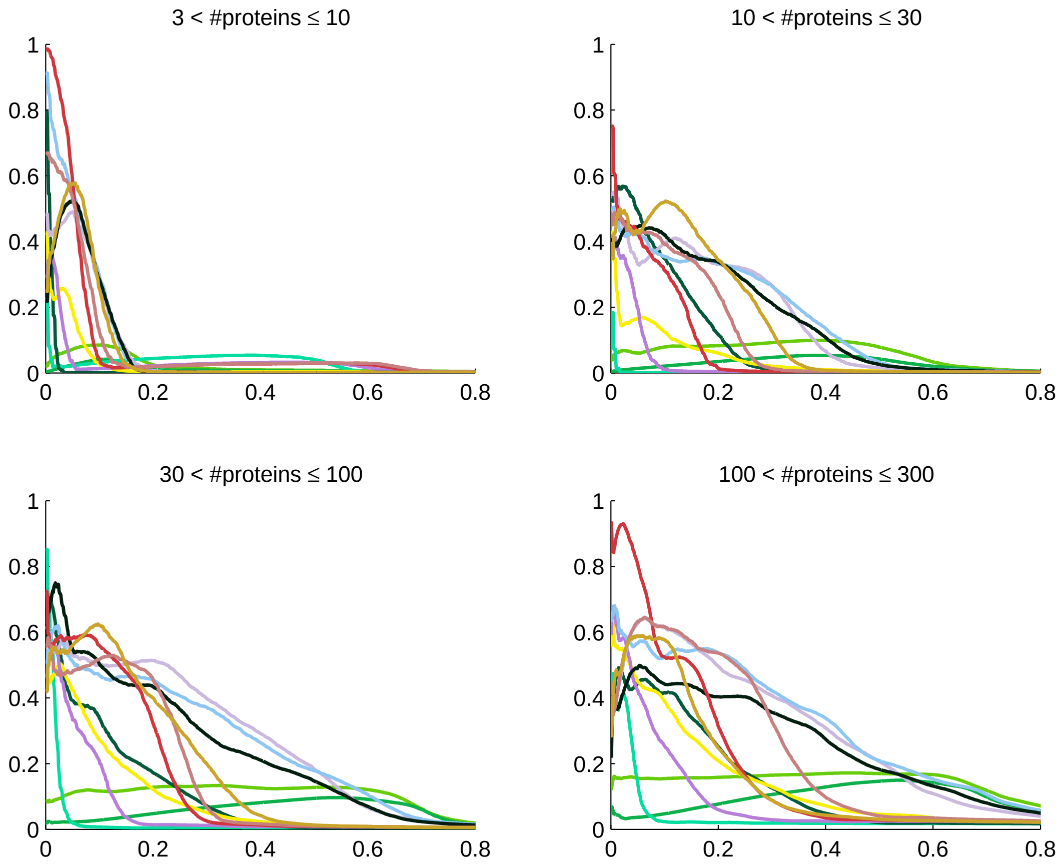

Figure S.46: **Precision-recall curves per size for joint annotation in the Cellular Component ontology (test set)** Precision-recall curve for 4 groups of terms with different levels of specificity in the ontology.

3.4 Zoomed precision-recall curves (recall < 10%) by ontology and term size

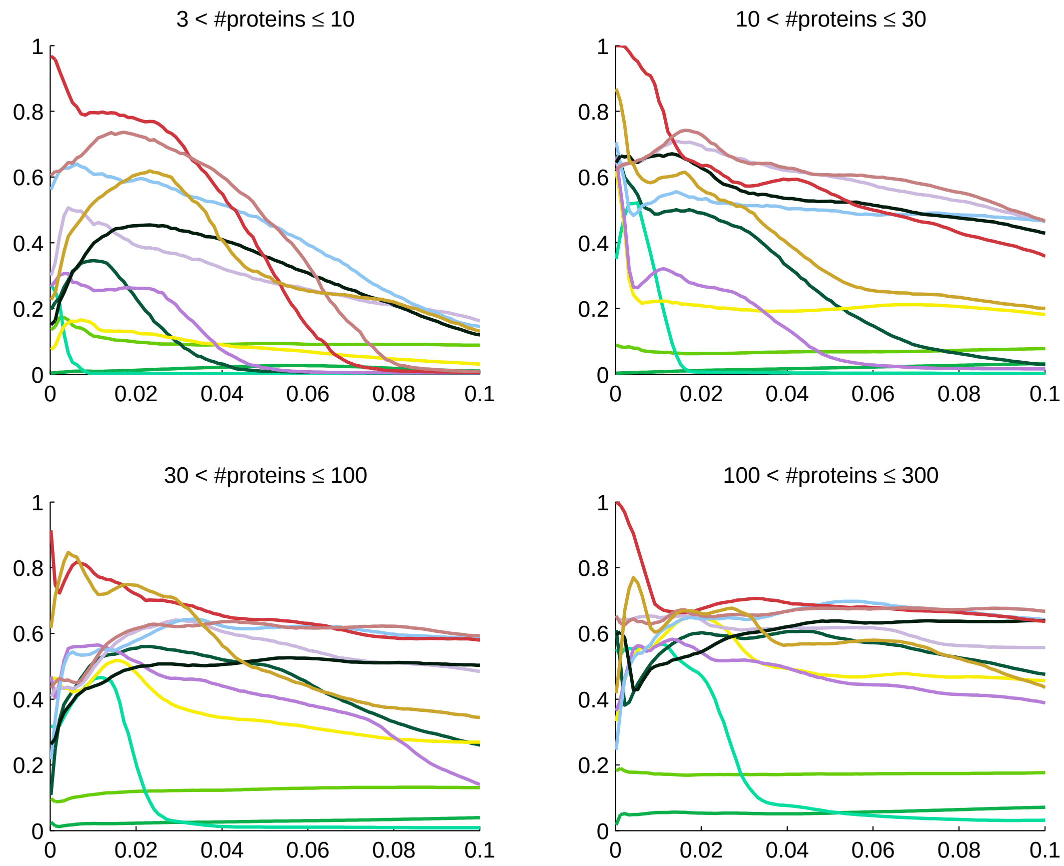

Figure S.47: **Truncated precision-recall curves for joint annotation in the Biological Process ontology (hold-out set)** A zoom-in of the high precision regime of the previous plot.

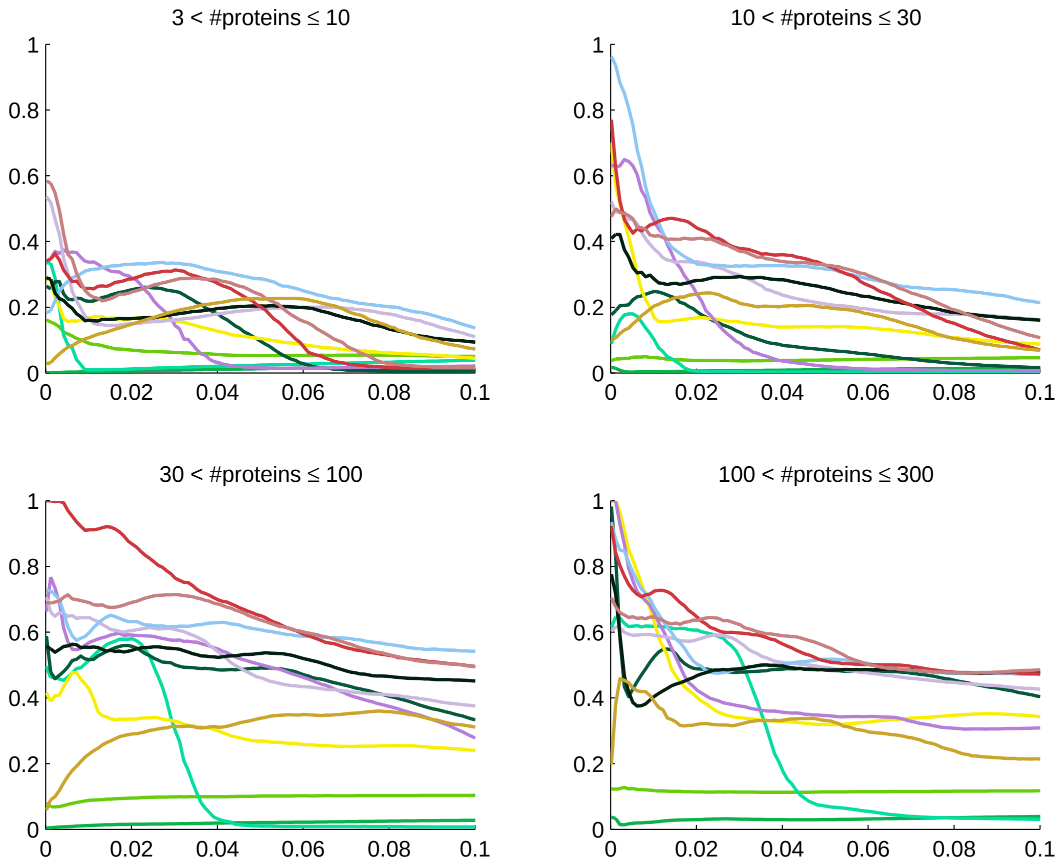

Figure S.48: **Truncated precision-recall curves for joint annotation in the Biological Process ontology (test set)** A zoom-in of the high precision regime of the previous plot.

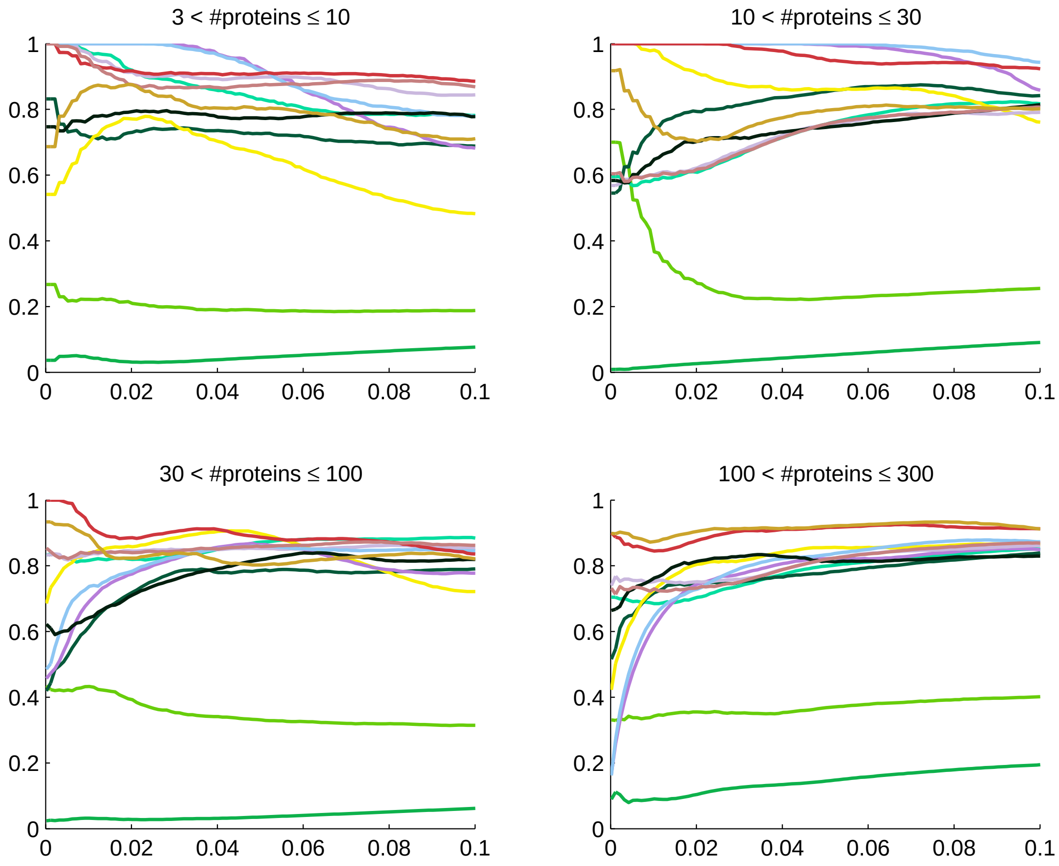

Figure S.49: **Truncated precision-recall curves for joint annotation in the Molecular Function ontology (hold-out set)** A zoom-in of the high precision regime of the previous plot.

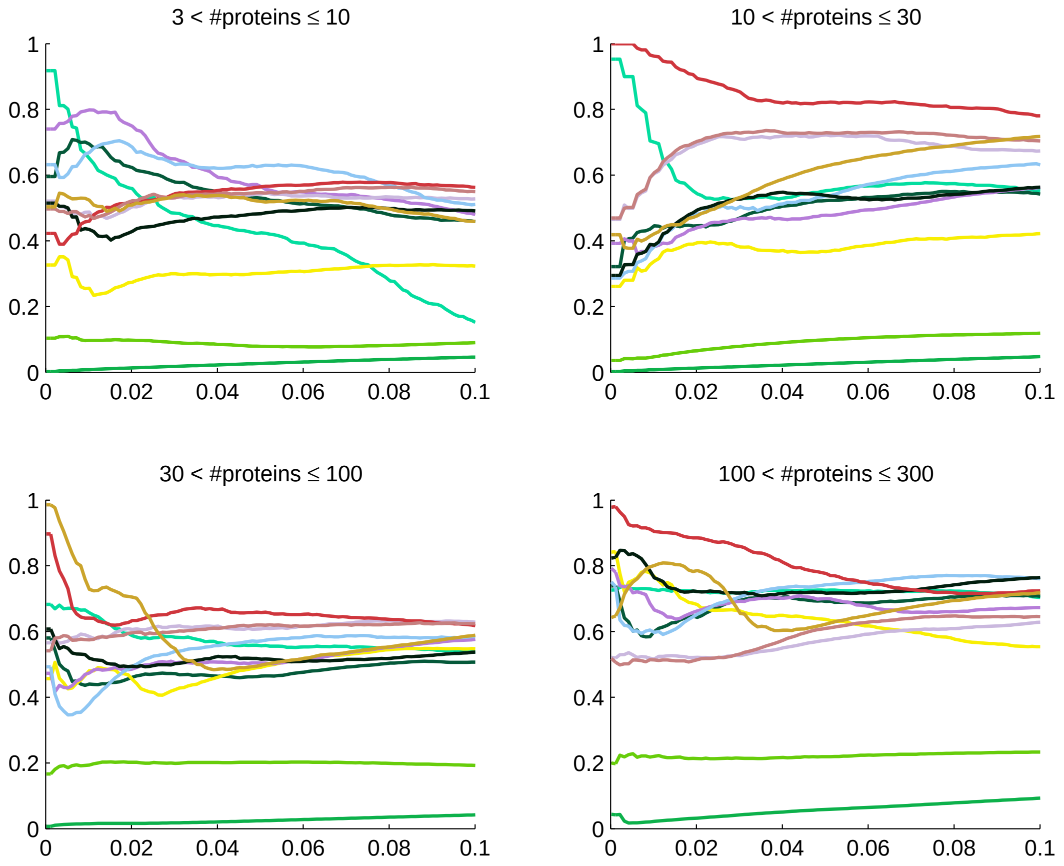

Figure S.50: **Truncated precision-recall curves for joint annotation in the Molecular Function ontology (test set)** A zoom-in of the high precision regime of the previous plot.

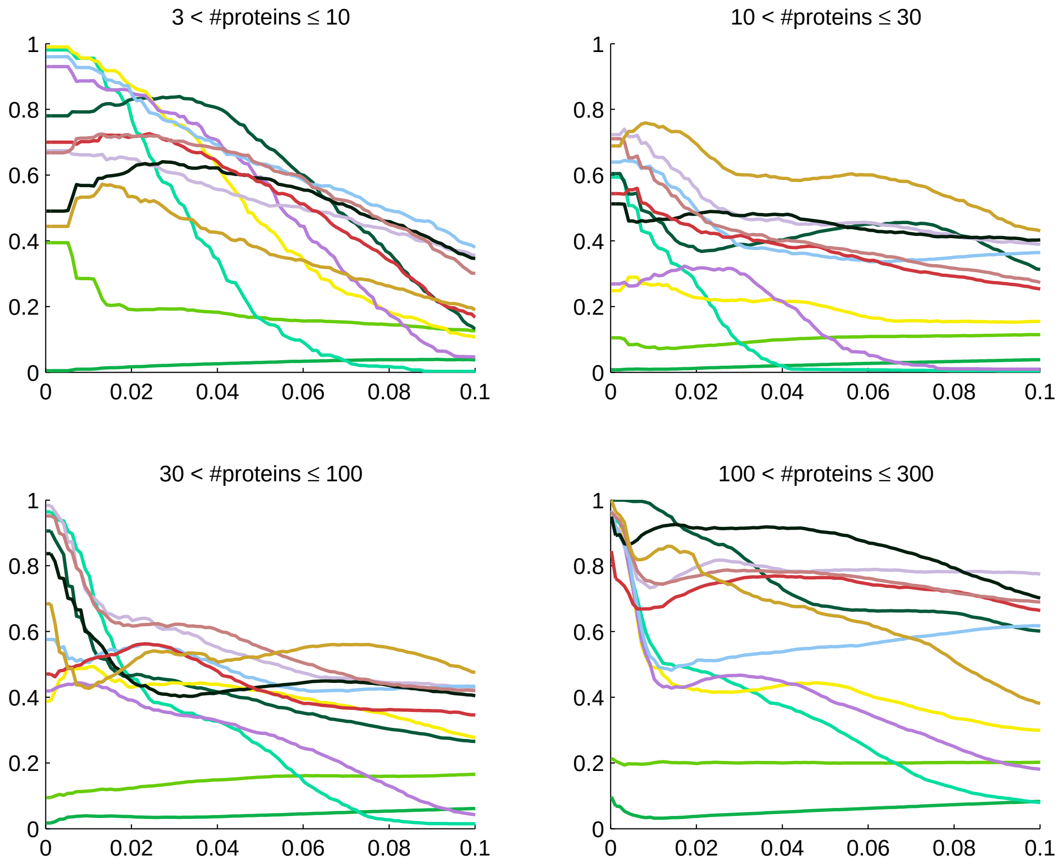

Figure S.51: **Truncated precision-recall curves for joint annotation in the Cellular Component ontology (hold-out set)** A zoom-in of the high precision regime of the previous plot.

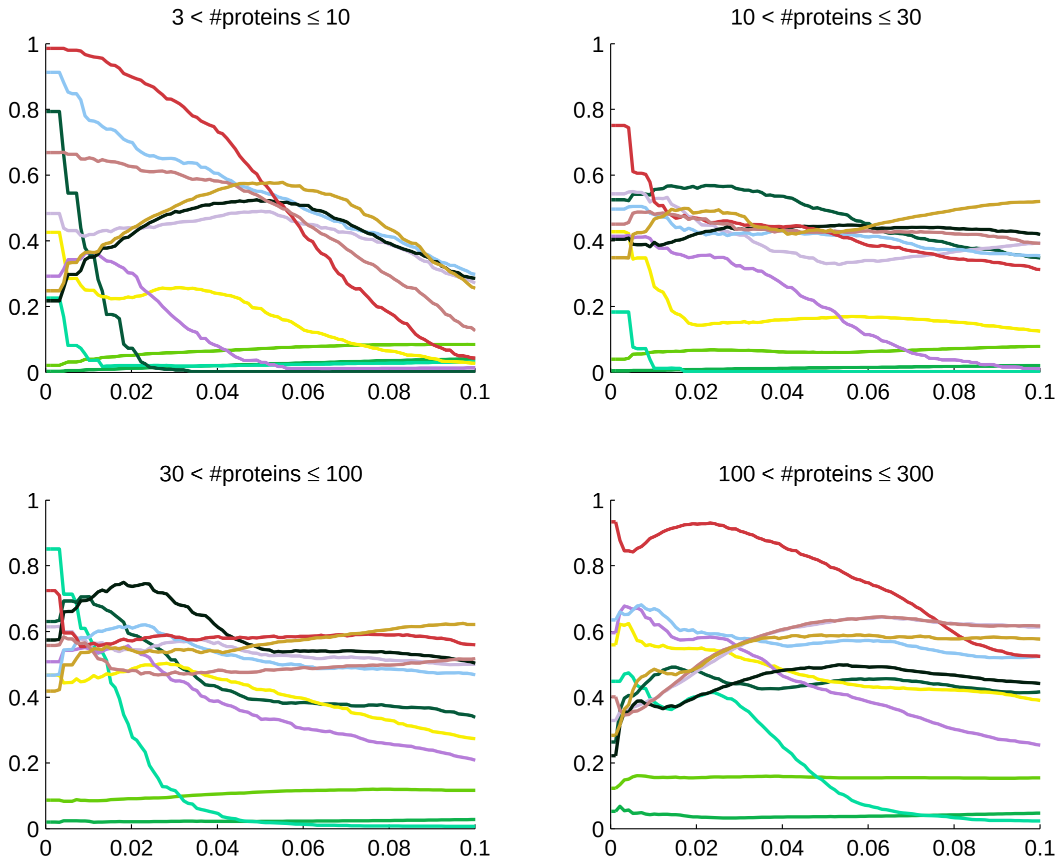

Figure S.52: **Truncated precision-recall curves for joint annotation in the Cellular Component ontology (test set)** A zoom-in of the high precision regime of the previous plot.

3.5 Directed graphs by ontology and term size

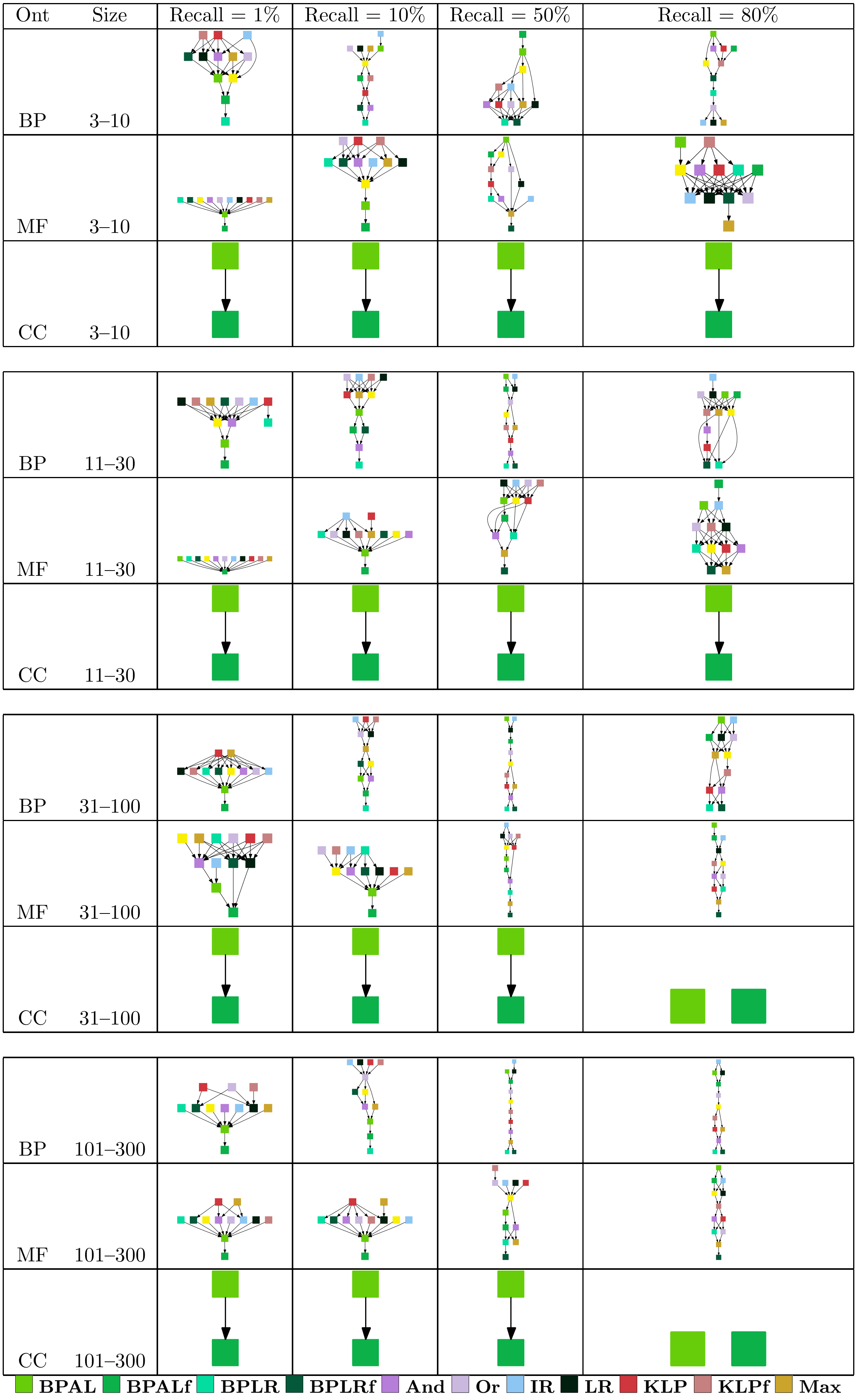

Figure S.53: **Statistical significance testing of joint evaluation (hold-out set)** Each panel shows a directed graph in which nodes are methods and a directed edge from node A to node B indicates that method A performs significantly better than method B according to the bootstrap test.

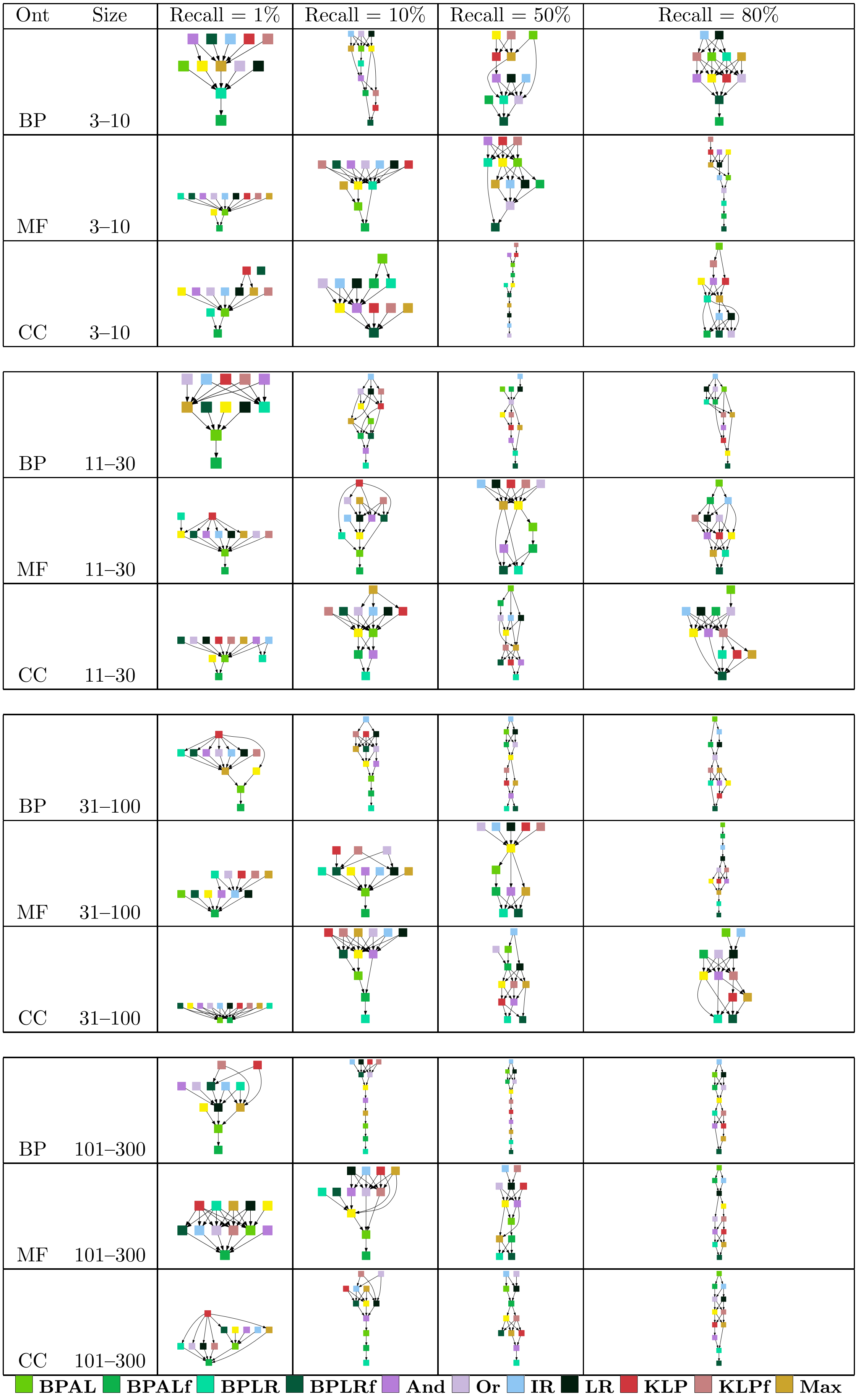

Figure S.54: **Statistical significance testing of joint evaluation (test set)** Each panel shows a directed graph in which nodes are methods and a directed edge from node A to node B indicates that method A performs significantly better than method B according to the bootstrap test.
